# Proteny: discovering and visualizing statistically significant syntenic clusters at the proteome level

**DOI:** 10.1093/bioinformatics/btv389

**Published:** 2015-06-23

**Authors:** Thies Gehrmann, Marcel J.T. Reinders

**Affiliations:** Delft Bioinformatics Lab, Delft University of Technology, Mekelweg 4, 2628 CD Delft, The Netherlands

## Abstract

**Background:** With more and more genomes being sequenced, detecting synteny between genomes becomes more and more important. However, for microorganisms the genomic divergence quickly becomes large, resulting in different codon usage and shuffling of gene order and gene elements such as exons.

**Results:** We present Proteny, a methodology to detect synteny between diverged genomes. It operates on the amino acid sequence level to be insensitive to codon usage adaptations and clusters groups of exons disregarding order to handle diversity in genomic ordering between genomes. Furthermore, Proteny assigns significance levels to the syntenic clusters such that they can be selected on statistical grounds. Finally, Proteny provides novel ways to visualize results at different scales, facilitating the exploration and interpretation of syntenic regions. We test the performance of Proteny on a standard ground truth dataset, and we illustrate the use of Proteny on two closely related genomes (two different strains of *Aspergillus niger*) and on two distant genomes (two species of Basidiomycota). In comparison to other tools, we find that Proteny finds clusters with more true homologies in fewer clusters that contain more genes, i.e. Proteny is able to identify a more consistent synteny. Further, we show how genome rearrangements, assembly errors, gene duplications and the conservation of specific genes can be easily studied with Proteny.

**Availability and implementation:** Proteny is freely available at the Delft Bioinformatics Lab website http://bioinformatics.tudelft.nl/dbl/software.

**Contact:** t.gehrmann@tudelft.nl

**Supplementary information**: Supplementary data are available at *Bioinformatics* online.

## 1 Introduction

A synteny analysis is a useful way to compare organisms that allows us to study the evolution between genomes, make claims about functional conservation (McClean *et al.*, 2010; Overbeek *et* *al.*, 1999), identify genome rearrangements (Sinha and Meller, 2007), aide genome annotation (Vallenet *et* *al.*, 2006) and even predict genome assembly errors.

Numerous tools are already available to detect synteny. Tools like Mugsy (Angiuoli and Salzberg, 2011), Mauve (Darling *et* *al.*, 2004), Multiz (Blanchette *et* *al.*, 2004) and Sibelia (Minkin *et* *al.*, 2013), focus only on highly related genomes. OrthoCluster (Zeng *et* *al.*, 2008) and SyMAP (Soderlund *et* *al.*, 2011) operate at the DNA level and discover groups of genes with their gene order being conserved. These assumptions are too strict when considering more distant genomes (see Supplementary Material S1).

i-ADHoRe (Proost *et al.*, 2012; Simillion *et al.*, 2008; Vandepoele *et* *al.*, 2002) works at the protein level and builds a homologous gene matrix based on protein–protein alignments, detecting clusters of genes by identifying diagonal groups of genes, allowing for a maximum gap size. However, for more distant genomes, exons may be inserted and removed from genes, while splice variants remain conserved (Long *et* *al.*, 2003). Consequently, it seems more reasonable to detect synteny between more distant genomes by considering the protein level at the resolution of exons, rather than the genes.

We introduce a method, called Proteny, which can discover statistically significant syntenic clusters between diverged genomes that may have different codon usages. Proteny analyses synteny at the exon level, so that more distant homologies can be revealed. As Proteny assigns a significance level to the detected syntenic clusters, it only requires setting a *P*-value cutoff and an intuitive parameter balancing the conservation ratio of the detected clusters.

Traditionally, synteny is visualized using dot-matrix plots such as those in R2Cat (Husemann and Stoye, 2010) and SyMAP (Soderlund *et* *al.*, 2011), which is useful to visualize the synteny between entire genomes but not when closely inspecting specific regions. Novel techniques such as (Shaw, 2008) can visualize synteny between many genomes at a lower level but quickly produces complicated figures when looking at very large regions or sufficiently different organisms. Easyfig (Sullivan *et* *al.*, 2011) can look at different levels and can be used to annotate interesting regions; however, it must be done manually. Cinteny (Sinha and Meller, 2007) provides multi-level visualizations to display synteny between multiple organisms, but it cannot visualize exons. With Proteny, we also introduce a user-friendly visualization for examining the discovered syntenic regions, which are especially useful when genomes are more distant.

Proteny is quantitatively benchmarked against a dataset from the Yeast Gene Order Browser that includes a gold standard of orthology relationships (Byrne and Wolfe, 2005), and it is compared to i-ADHoRe. We demonstrate the utility of Proteny on two fungal datasets: (i) two *Aspergillus niger* genomes that are known to be highly related, illustrating how Proteny can be used to explore the similarities and differences between two genomes and (ii) two mushroom forming fungi (of the phylum basidiomycota) *Schizopyllum* *commune* and *Agaricus bisporus*, demonstrating the power of Proteny to detect syntenic regions between more distant genomes which also differ in their codon usage (see Supplementary Material S1). As there is no gold standard for these datasets, we qualitatively analyze the discovered clusters.

## 2 Methods

### 2.1 General overview

**Fig. 1.**
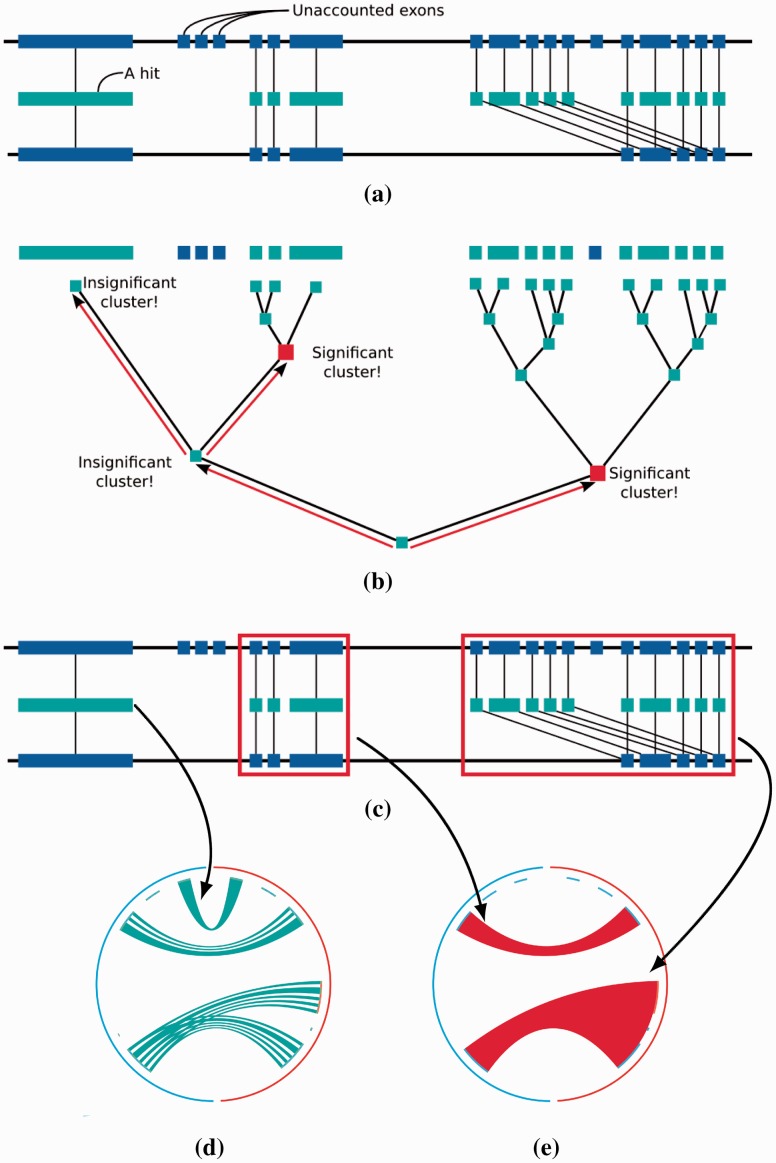
An illustration of how Proteny works. (**a**) First, BLASTp is used to produce a set of hits, which are used to build (**b**) a dendrogram which is traversed to find (**c**) significant clusters (red boxes). (**d**) Individual hits are displayed (here in turquoise) in a region visualization, while (**e**) significant clusters are displayed (here in red) in a chromosome visualization

Proteny detects syntenic clusters by translating all exon regions into protein sequences and producing a set of BLASTp hits (Fig. 1a). Proteny then calculates a distance between all hits based on genomic distance, resulting in a distance matrix. From this distance matrix, Proteny builds a dendrogram where each node represents a cluster of hits (Fig. 1b). The dendrogram is traversed in a depth first procedure, searching for clusters with significant scores based on a statistical test. Each cluster is scored depending on the hits which are found within the cluster and the number of unaccounted exons (exons without hits) that lie within the genomic regions that the cluster covers. When a significant cluster is found (and its child is not *more* significant), the branch is cut (i.e. no smaller clusters are evaluated in that branch). Proteny terminates when no more significant clusters can be found, culminating in a set of significant clusters of hits (Fig. 1c). These clusters can then be visualized by looking at the individual hits (Fig. 1d) or at a higher level (Fig. 1e).

### 2.2 Obtaining a mapping

A mapping from organism β to organism γ is a set of pairs, whereby a locus in organism β is linked to a locus in organism γ. Proteny links loci on their translated sequence similarity. For that, all exons in each organism are translated to construct two BLAST databases (Altschul *et* *al.*, 1997) and two sequence sets for each genome. A bi-directional BLASTp (using default parameters) then produces a mapping, i.e. a set of bi-directional hits $h_{i}\in H$, between sequences from the two organisms describing a similarity between two sequences. Consequently, a hit represents two regions, $h_{i}=(r_{i}^{\beta },r_{i}^{\gamma })$, which correspond to the genomic location of subsequences of exons in the genomes of organisms β and γ, respectively. A region has a start and an end, i.e. $r_{i}^{\chi }=(s_{i}^{\chi },e_{i}^{\chi })$, where χ corresponds to an organism. All these variables are clarified in Supplementary Figure S3a.

### 2.3 Distances between hits

To cluster hits, we need a definition of similarity between them, which we base on the distance between their associated regions. The distance between two regions on the same genome is given by Equation (1).

(1) $$\text{dist}(r_{i}^{\chi },r_{j}^{\chi })=\max\left\{0,\max(s_{i}^{\chi },s_{j}^{\chi })-\min(e_{i}^{\chi },e_{j}^{\chi })\right\}$$

The distance between two hits is then defined as the sum of the distance between the two regions on one genome and the distance between the two regions on the other genome.

(2) $$d(h_{i},h_{j})=\text{dist}(r_{i}^{\beta },r_{j}^{\beta })+\text{dist}(r_{i}^{\gamma },r_{j}^{\gamma })$$

Note that when two regions overlap (i.e. their distance is zero), they do not contribute to the distance between two hits. Supplementary Figure S3b illustrates the distance between two hits as the sum of the distances between the regions they involve. In Supplementary Figure S3c, we see an example of an exon duplication and two hits referring to the same exon.

### 2.4 Constructing a dendrogram

Using this distance measure between hits, we construct a dendrogram through a single linkage hierarchical clustering. An example is shown in Figure 1b. We first group all hits by the chromosomes on which the hits fall. For any pair of chromosomes (each on a different genome), Proteny builds a dendrogram, in which each node represents a cluster of hits. It is important to note that we cluster not exons but hits. This strategy may result in an exon being present in multiple clusters (and multiple dendrograms), allowing us to handle duplications.

However, the height of the tree reflects only the density of hits not the quality of conservation within. Cutting the tree to produce clusters based on height alone will therefore not be sufficient. Instead, we define a cluster score which reflects our expectations of syntenic clusters.

### 2.5 A cluster score

As in Ghiurcuta and Moret (2014), we consider a syntenic region good if it maximizes the similarity within the cluster and minimizes the similarity between them. We characterize each cluster with a cluster score, which describes the similarity within the cluster but is punished by the similarity to other regions. The similarity within the cluster is described by the quality of the hits which lie within the region, and the similarity to other regions is described by the quality of hits which fall within the genomic region defined by the cluster but have no hits within the cluster (unaccounted exons). The quality of a hit should reflect the coverage of the hit over the exons it covers and the significance of this hit. We therefore define a quality score $K(h_{i})$ for a hit $h_{i}\in H$ between two exon sequences:

(3) $$K(h_{i})=\left\{1-\min(1,E(h_{i}))\right\}\cdot \frac{\left||r_{i}^{\beta }||+||r_{i}^{\gamma }|\right|}{\left||x_{i}^{\beta }||+||x_{i}^{\gamma }|\right|}\;,$$

where $x_{i}^{\chi }$ is the exon the hit *h_i_* refers to on genome χ, ||·|| is the length of a given sequence or region and $E(h_{i})$ is the e-value of the hit. The ratio represents the fraction of the size of the exons which are covered by the hits, favoring hits which cover the whole exon. This ratio is multiplied by $1-E(h_{i})$ to factor in the significance of the hit, so that insignificant hits will deteriorate the score. Note that K(·)∈[0,1] where 1 is the perfect score.

Then, the cluster score, *s*(*C*), accumulates the hit scores for the hits within the cluster but is penalized by exons within the cluster which do not have a hit in the cluster *C*:

(4) $$s(C)=2\cdot \sum_{h_{i}\in C}K(h_{i})-\sum_{e\in U_{C}^{\beta }\cup \;U_{C}^{\gamma }}\max_{h_{j}\in H_{e}}K(h_{j})\;,$$

where $U_{C}^{\chi }$ is the set of exons on genome χ which are located within cluster *C* but are unaccounted for within the cluster, and *H_e_* are all the bi-directional BLASTp hits to exon *e* (for *e* from organism β or γ). If *H_e_* is empty (i.e. the unaccounted exon has no hit to the other genome), then the cluster is not penalized (see Supplementary Fig. S3d).

Note that the penalization for unaccounted exons is based on the maximum hit score. The main motivation for this is that if an unaccounted exon has a better hit somewhere else then it should not be in the current cluster. However, if the unaccounted exon does not have a hit anywhere on the other genome (*H_e_* being empty), then, without knowing anything more about it, it should not affect the cluster score.

### 2.6 A dynamic cutting algorithm

Proteny cuts the dendrogram at a given node depending upon the significance of the cluster score assigned to that node (see next section). However, some clusters contain so many good hits that they may contain many large gaps (unaccounted exons), while still being significant. To counter that, we restrict ourselves to clusters which satisfy a minimum ‘conservation ratio’, given by the user-specified parameter τ. The conservation ratio τ*_C_* of a cluster *C*, is defined as $\frac{n_{C}+1}{n_{C}^{\beta }+n_{C}^{\gamma }+1}$, where $n_{C}=|C|$, the number of hits in the cluster, $n_{C}^{\beta }=|U_{C}^{\beta }\cap H|$, the number of unaccounted exons on genome β which have a hit elsewhere and $n_{C}^{\gamma }=|U_{C}^{\gamma }\cap H|$, the number of unaccounted exons on genome γ, that have a hit elsewhere.

The dendrogram will therefore not be cut at a single height but at different heights depending on the significance and the conservation ratio. For such an approach, a ‘dynamic tree cut’, other methods exist (Langfelder *et* *al.*, 2008; Mason *et* *al.*, 2009), but those do not rely upon a statistical significance to cut. We use a greedy cutting algorithm, given in SE-8. Starting at the root node, check if the current node satisfies the conservation ratio and has a lower *P*-value than its child nodes. If both are true, and the node is significant, then we cut at this node and we do not descend further into the tree. Alternatively, if the current node is not significant or either of the child nodes have a lower *P* value and satisfy the conservation ratio, we descend instead to the children.

### 2.7 Testing the significance of a cluster

To calculate the significance of a cluster, we must build a null distribution of cluster scores. Other methods which calculate the statistical significance of a cluster such as Jahn *et* *al.* (2013) do not take into account the similarity between clusters that our cluster score does. Therefore, we must build our own null distribution of cluster scores for each particular size of cluster (i.e. combination of *n_C_*, $n_{C}^{\beta }$ and $n_{C}^{\gamma }$). Although a null distribution constructed from hits with random scores that are randomly distributed along the genome would be ideal, it is computationally infeasible as we would need to re-cluster at every iteration. Instead, we permute hit scores after the clustering, thereby assuming no fixed structure in successive hits, as would be the case if the hits were randomly distributed. Hence, the cluster score for one permutation becomes:

(5) $$s_{p}(C)=2\cdot \sum_{k=1}^{n_{C}}P_{k}-\left(\sum_{k=1}^{n_{C}^{\beta }}P_{k}^{\beta *}+\sum_{k=1}^{n_{C}^{\gamma }}P_{k}^{\gamma *}\right)$$

where *P_k_* is the *k*th element of a randomly shuffled list of all bi-directional BLASTp all bi-directional BLASTp hit scores *H* (created by random reordering), and $P_{k}^{\chi *}$ is the *k*th element of a randomly permuted list of only best bi-directional BLASTp hits for each exon in organism χ (by taking only the best hit for each exon).

*P* values can now easily be obtained by comparing the actual cluster score to the permuted scores. However, since many nodes in the dendrograms are tested, we need to correct for multiple testing. For a pair of organisms with |H| hits between them, we would in the worst case perform 2|H| tests, calling for a Bonferroni correction of 2|H|. With such a correction, and a *P*-value threshold of α, at least $\left\lceil \frac{2|H|}{\alpha }\right\rceil$ permutations would be required just to achieve the resolution required to detect a cluster. This correction factor, and thereby the number of permutations can become very high, and we therefore wish to limit the number of permutations when possible.

Unfortunately, the inheritance procedure of Goeman and Finos (2012), which controls the family-wise error rate for hierarchical tests does not apply, since our problem does not fulfill the condition that significant tests must have significant parent tests. Similarly, the same condition for the false-discovery-rate correction for trees of Yekutieli (2008) is not met. We integrate four approaches which help us making the number of permutations more tractable.

#### 2.7.1 Not considering all clusters

As we are interested in synteny (beyond homology statements between genes), we are not interested in clusters which are smaller than two genes, nodes in the dendrogram which contain fewer than two genes are not tested.

#### 2.7.2 Early stopping

We can apply the method of Knijnenburg *et* *al.* (2009), to limit further permutations when the number of exceedences is already sufficient. The cluster which is then not significant, will never be significant with more permutations. As we only wish to detect significant clusters, we can apply this strategy.

#### 2.7.3 Analytical solution

If a cluster is large enough (see Supplementary Material S4.1), we can make use of an analytical description of the null distribution, based on the central limit theorem (CLT) described in Supplementary Equation S6. The cluster score is a sum of three different distributions, each component being a summation over random variables. Consequently, where possible, we use the CLT approximation for the null distribution. We revert to the permutation method if the cluster size is too small.

#### 2.7.4 Dynamic correction

Rather than using a worst-case multiple testing scenario to determine the number of tests to correct for, we determine the number of tests dynamically. That is, we start out by setting the initial number of tests to 1, and, following the dynamic cutting algorithm (SE-8), we increase the correction factor only when we descend to a child node in the tree. Alternatively, if a node is called significant, we do not need to increase the correction factor.

For insignificant nodes, this is always allowed since it will only be *more* insignificant at higher correction factors. However, significant nodes will have to be revisited (since with the larger correction factor, they may become insignificant). The advantage here is that when we need to revisit a node, we only need to do the additional permutations; i.e. we can still make use of the earlier permutations. This procedure is performed iteratively until no further tests are performed.

### 2.8 Visualization

Proteny provides two different types of visualization: (i) a *chromosome*-level visualization and (ii) a *region*-level visualization. Chromosome-level visualizations allow us to have an overview of the relationships between two genomes. In this visualization (e.g. Fig. 3), the outer ring is the genome, the inner ring represents the genes (blue and orange representing genes on the forward and reverse strand, respectively) and the ribbons between two loci represent a conserved cluster. The query chromosome is shown first, in a clockwise-fashion from 12 O’clock onward.

The region-level visualizations show only a few loci from both genomes (e.g. Fig. 4e). Again, the outer ring represents the regions on the genome and the inner ring represents genes. Now, additional green boxes within the genes represent exons. The intensity of each link represents the quality score K(·) of the hit. The ribbons no longer represent clusters, rather, they are the original BLASTp hits between exons.

### 2.9 Implementation details

For data handling, we use the Ibidas (Hulsman *et* *al.*, 2013) data query and manipulation suite, and the Circos (Krzywinski *et* *al.*, 2009) utility is used to visualize the discovered clusters. For more information, see Supplementary Material S6.

## 3 Results

### 3.1 Yeast gene order browser dataset

The YGOB (Byrne and Wolfe, 2005) provides a ground truth through a large set of ortholog relationships between 20 yeast genomes. We use the data and scores described in Ghiurcuta and Moret (2014) to compare Proteny to i-ADHoRe. Since Proteny performs a pairwise synteny discovery analysis, the two scores are equivalent. We use the same parameters for i-ADHoRe and fasta36 (Pearson, 1998) as given in Ghiurcuta and Moret (2014). For Proteny, a *P*-value threshold of 0.05 and a conservation threshold of 1. We score the clusters that i-adhore and Proteny find using the relaxed score in (Ghiurcuta and Moret, 2014).

**Fig. 2.**
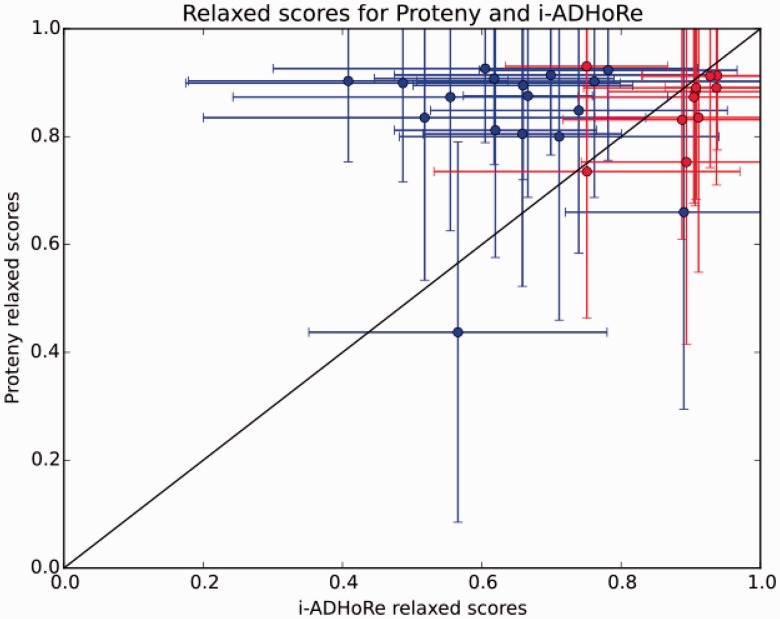
The means and their standard deviations of relaxed scores across all syntenic clusters for Proteny (*y*-axis) and i-ADHoRe (*x*-axis) clusters, for each pairwise test. Red points are cases where the relaxed score distributions are not significantly different between Proteny and i-ADHoRe (*q* < 0.5 Kolmogorov–Smirnov test with Bonferroni multiple testing correction)

Figure 2 shows the means of the relaxed scores for all clusters in each of the pairwise tests for both Proteny and i-ADHoRe. Proteny had a higher average cluster score in 16 out of 28 experiments. In 15 of these, the relaxed cluster score distributions were significantly different (by a Bonferroni corrected Kolmogorov–Smirnov test), see also Supplementary Figure S12c. Although i-ADHoRe had higher average relaxed scores in 12 experiments, in 10 of these cases, the distributions of relaxed scores are not significantly different. Based on this, Proteny either performs comparably to or outperforms i-ADHoRe on this dataset.

### 3.2 Aspergillus niger

We study two strains of *A.niger*, which have been separated by 50 years of evolution, n402 and CBS513.88. CBS513.88 is an industrial strain, which is used as a cell factory for enzyme and metabolite production, while n402 is a laboratory strain used in research. We use this to demonstrate the performance of the method. Since we know that the two strains must be highly related, we expect to find large similarities between the two genomes. The *A.niger* CBS513.88 genome (Pel *et* *al.*, 2007) and annotation were retrieved from the Aspergillus genome database (Arnaud *et* *al.*, 2010). The *de novo* genome sequence of laboratory strain *A.niger* n402 was unpublished at time of writing (see Acknowledgements). The n402 strain has 13 612 genes, whereas the industrial strain CBS513.88 has 14 067 genes. Because of incomplete genome assemblies, we deal with scaffolds rather than chromosomes. The n402 and CBS513.88 strains have 24 and 19 scaffolds, respectively. For this dataset, we set τ = 2, because we assume that the two strains are quite similar.

#### 3.2.1 General synteny

Proteny finds high conservation between the two strains. In total, Proteny finds 119 syntenic clusters, covering 10 880 n402 genes and 10 956 genes in CBS513.88 (see Supplementary Table S2). We compare Proteny’s results to those of i-ADHoRe, as it is the only tool that also works on the protein level and is not specifically designed for similar genomes. i-ADHoRe finds 189 syntenic clusters, covering 9667 (9310 in common with Proteny) and 9728 genes (9343 in common) from the n402 and CBS513.88 strains, respectively. We find that 66.5% of the area covered by the clusters discovered by Proteny and i-ADHoRe is found by both algorithms.

By calculating the score for each i-ADHoRe cluster using our scoring function, we find that only 93 (49.2%) of the clusters that i-ADHoRe finds are significant (see Supplementary Table S6), and most have a very small conservation ratio (see Supplementary Fig. S10b). Furthermore, we see in Figure 4f, that Proteny generally has more genes in i-ADHoRe clusters of the same size. From this we conclude that Proteny finds more genes in fewer clusters. Apparently, Proteny discovers informative clusters that tightly describe the syntenic genes.

#### 3.2.2 Identifying a genome rearrangement

**Fig. 3.**
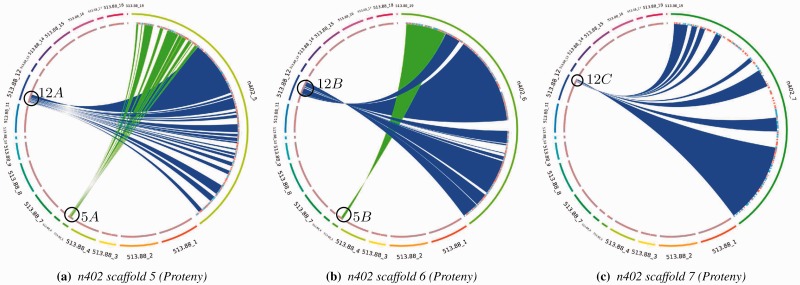
Syntenic clusters found for *S.commune* on (**a**) scaffold 5, (**b**) scaffold 6 and (**c**) scaffold 7 in n402. For scaffolds 5 and 6, one can see that n402 has undergone a rearrangement

Figure 3a and b shows the syntenic clusters Proteny discovers for n402 scaffolds 5 and 6, respectively. These figures show that *A.niger*n402 was formed by a rearrangement: parts 5*A* and 12*A* from CBS513.88 (Fig. 3a) have fused together to form scaffold 5 in the n402 strain. Likewise, scaffold 6 from n402 was formed by the fusion of parts 5*B* and 12*B* (Fig. 3b). From these detected syntenic regions, one can conclude that scaffolds 5 and 12 of CBS513.88 have split in two and fused together over time to form two scaffolds in n402. When comparing with i-ADHoRe (Supplementary Fig. S5a–c), we see that Proteny gives a clearer synteny (i.e. i-ADHoRe is cluttered with other supposed syntenies), and at the same time, Proteny gives more detail on the fused or separated syntenic regions.

**Fig. 4.**
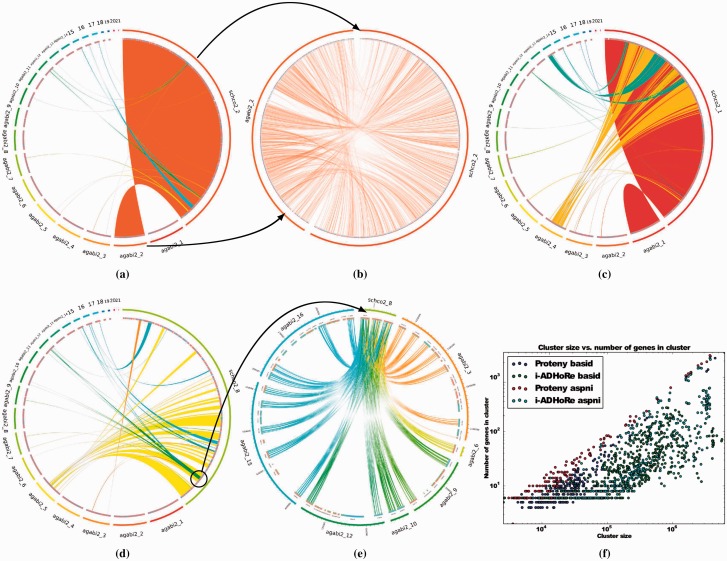
(**a**) The synteny between scaffold 2 in *S.commune* and the scaffolds in *A.bisporus.* (**b**) The hits between scaffold 2 in *S.commune* and scaffold 2 in *A.bisporus* show a scattered synteny. (**c**) The syntenic relationships between scaffold 1 in *S.commune* and the scaffolds in *A.bisporus.* The three scaffolds 1, 4 and 14 share a lot in common with scaffold 1 in *S.commune*, and it follows that, at some point the two species diverged when the scaffold split in the branch of *A.bisporus* but not in the branch of *S.commune.* (**d**) There is an interesting region on scaffold 8 of *S.commune.* (**e**) A repeated gene elucidates a divergent trait between *A.bisporus* and *S.commune.* (**f**) A Proteny cluster of a given size generally has more genes than an i-ADHoRe cluster of the same size. In this figure, ‘*basid’* refers to the basidiomycete analysis, and ‘*aspni’* refers to the *A.niger* analysis. It can be seen in this figure that Proteny cluster gene densities are higher than those of i-ADHoRe and a *t*-test with unequal variance assumptions states that the distributions of cluster scores are separated with a *P* value of $9.1\times 10^{-75}$

#### 3.2.3 Assisting genome assembly

Proteny can also assist in genome assembly. By studying the visualizations, we can quickly inform ourselves about the results of an assembly. Figure 3c shows that scaffold 7 of n402 maps to part 5*C* from CBS513.88, whereas according to Figure 3b, scaffold 6 of n402 maps to the connecting part 5*B* in CBS513.88. This indicates that scaffolds 6 and 7 in the n402 assembly could be joined together.

The effect is even more pronounced in Supplementary Figure S7a–c. We see that three chromosomes in n402 map to a single chromosome in the CBS513.88 genome. Proteny can guide an assembly and suggest that they be joined together in the n402 genome, as in the CBS513.88 genome.

### 3.3 Basidiomycota

Next, we applied Proteny to *S.commune *(Ohm *et* *al.*, 2010), a model organism for mushroom formation, and *A.bisporus *(Baker *et* *al.*, 2013), which is a commercially valuable mushroom but has a relatively large evolutionary distance to *S.commune.* We retrieved the genomes and genome annotation files for *S.commune*v2.0 and *A.bisporus*v2.0 from the JGI genome portal (Grigoriev *et* *al.*, 2012). *S.commune* has 14 652 genes, and *A.bisporus* has 10 438 genes. As before, these organisms have incomplete genome assemblies, with 36 and 31 scaffolds, respectively. For this experiment, we set τ = 1, because while we assume a lot of divergence, we are interested in conserved clusters.

#### 3.3.1 General synteny

Proteny finds 345 significant clusters, covering 5828 *S.commune* genes lying within conserved regions, and 4572 *A.bisporus* genes (see Supplementary Table S2). Many exons do not have a bi-directional BLASTp hit, resulting in many smaller clusters. i-ADHoRe discovers 377 clusters which cover 2588 *S.commune* genes (2889 in common with Proteny) and 4090 *A.bisporus* genes (2662 in common). The area (41.4%) covered by the clusters discovered by Proteny and i-ADHoRe is found by both algorithms. From these clusters (see Supplementary Table S7), we find that, using our scoring function, only 327 (57.1%) i-ADHoRe clusters are significant. The reason we find so many more genes than i-ADHoRe stems from the orderless detection of the clusters. The results from Proteny show that although both mushrooms are evolutionarily distant, a large portion of the genes remain conserved.

Figure 4f shows the genomic size of a Proteny cluster is smaller than that of an i-ADHoRe cluster containing the same number of genes. The figure also shows that a Proteny cluster of a given size generally has more genes than an i-ADHoRe cluster of the same size. i-ADHoRe clusters contain more unaccounted exons (gaps), confirmed by Supplementary Figure S10b and e. Again, we conclude that Proteny finds fewer clusters which harbor more genes.

#### 3.3.2 Large similarities

We even observe large similarities between the diverged genomes, as shown in Figure 4a between scaffold 2 in *S.commune* and scaffold 2 in *A.bisporus.* When we look at this cluster more closely in Figure 4b, we see that the hits are very dense. Figure 4a also shows that Proteny results in a much clearer synteny between the scaffolds than i-ADHoRe (Supplementary Fig. S6b), which includes many other clusters which occlude the results. This can be attributed to the result of the orderless synteny detection of Proteny. i-ADHoRe discovers more clusters, which contain large stretches of gaps between genes. For example, the additional cluster between chromosome 2 in *S.commune* and chromosome 7 in *A.bisporus* seen in Supplementary Figure S6b, which is not found by Proteny in Figure 4a, contains only a few spurious hits between a few genes with many unaccounted exons (Supplementary Table S6, no. 141).

#### 3.3.3 Scattered synteny

Figure 4c shows that scaffold 1 of the *S.commune* assembly consists primarily of three scaffolds in the *A.bisporus* assembly, over a number of syntenic blocks. Clearly, the three *A.bisporus* scaffolds 1, 4 and 14 have a lot in common with scaffold 1 in *S.commune*, and it follows that at some point the two species diverged when the scaffold split in the branch of *A.bisporus* but not in the branch of *S.commune.* Alternatively, the ‘scattered’ effect may be the result of an incorrect assembly of scaffolds 1, 4 and 14 in *A.bisporus.* Note that despite the syntenic regions between the two fungi being highly scattered, Proteny is able to detect the syntenic relationships and the visualizations allow us to explore the discovered clusters. For i-ADHoRe, the results are harder to examine, see Supplementary Figure S6a.

#### 3.3.4 Gene duplication

Figure 4d shows the syntenic clusters detected in scaffold 8 of *S.commune.* We see an interesting phenomenon here: there is a region in *S.commune,* which is repeated several times in *A.bisporus.* Figure 4e zooms in on this region. Here it becomes clear that there are three genes which are duplicated many times in *A.bisporus.* These genes are cytochrome P450 monooxygenases, which are involved in metabolism detoxification (Crešnar and Petrič, 2011), and are expected to be involved in the detoxification of byproducts from lignin degradation. The fact that *S.commune* has fewer copies of the P450 compared with *A.bisporus* highlights the fact that *S.commune* does not have the ability to degrade lignin, while *A.bisporus* does. The speciation event which separated *S.commune* and *A.bisporus* came before *A.bisporus* was able to degrade lignin and can be derived from the number of P450 copies in *A.bisporus.* Figure 4e reveals that many of these duplications are not entirely conserved, often exons are missing or new ones are there instead, exemplifying the benefit of the exon-level analysis. This again shows the capabilities of Proteny (i-ADHoRe does not detect this region, Supplementary Fig. S6c).

#### 3.3.5 Developmental proteins are conserved

We are particularly interested in eight transcription factors and a light sensing protein which have been linked to mushroom formation in *S.commune *(Ohm *et* *al.*, 2012). To increase the confidence that these transcription factors are functionally similar in both *S.commune* and *A.bisporus*, we wish to find that these genes lie in syntenic regions. Proteny reveals that six of these nine developmental proteins lie within conserved clusters. Supplementary Figure S8c shows the region-level plot for the cluster which contains the transcription factor *gat1.* The figure clearly shows that the transcription factor lies in a well-conserved region, i.e. neighboring genes in *A.bisporus* match to neighboring genes in *S.commune.* Supplementary Figure S8 shows the region-level plots for the clusters of the other developmental proteins found in syntenic clusters, and Supplementary Figure S9 shows the developmental proteins which were *not* found.

## 4 Discussion

We presented Proteny, a methodology which identifies significant conserved syntenic clusters of exons between two genomes through a novel method for cutting dendrograms and a new dynamic multiple testing correction algorithm. Knowledge of the discovered clusters allow us to uncover genome rearrangement events (as shown for both the *A.niger* strains and the Basidiomycota), make more motivated statements about functional conservation (as for *S.commune*), identify possible errors in the assembly of related genomes (like in *A.niger*) and study the evolution between species (as in looking at the cytochrome P450 monooxygenases in *A.bisporus* and *S.commune*).

When comparing with i-ADHoRe, the most competitive tool, on a ground truth dataset, we find Proteny outperforms i-ADHoRe. Qualitatively, we observe that i-ADHoRe finds more clusters, covering fewer genes than Proteny clusters. Proteny finds gene-dense clusters of high quality, as verified by the cluster scores achieved by Proteny on the YGOB dataset. This can be attributed to the statistical testing procedure and the conservation ratio we enforce in Proteny.

One practical advantage of Proteny over other synteny tools is that, besides the BLASTp settings (for which we used default values in our experiments), it only requires specifying a significance threshold (which can be set by statistical reasoning) and a conservation ratio parameter τ. It should be noted that Proteny could work with other aligners also and that BLASTp could be replaced by other protein sequence aligner.

On the other hand, i-ADHoRe and many other tools are able to perform an analysis on more than two genomes at a time. Proteny could be generalized towards any number of species by a progressive heuristic similar to the star multiple alignment heuristic (Altschul and Lipman, 1989), which uses a central sequence with pairwise sequence alignments to guide the multiple alignment.

It is important to note that the cluster score of Proteny does not account for the conservation of the order of the exons within the cluster. This can most prominently be seen in the synteny between scaffold 2 of *S.commune* and scaffold 2 of *A.bisporus* (Fig. 4a and b), where the order of the hits is scrambled. Although the ordering of the exons does play a role when constructing the dendrogram (nearby hits are merged first), we chose that the ordering should not play a role when scoring the clusters. This was a deliberate choice since Proteny was designed to find synteny between relatively divergent organisms in a microbiology context where evolution is fast; insertions, inversions, strand changes and gene shuffling occur frequently. Clearly, in other problem settings, the order may be important, in which case the cluster score in Proteny should be adjusted. However, one should be careful when designing a corresponding permutation scheme, e.g. a circular permutation of hit scores to preserve the order of hits in that set, as this might result in computational difficulties, as (e.g.) the CLT approximation will not hold anymore.

By searching for synteny at the exon level, we exclude the influence of noncoding regions of the genome, which are typically not well conserved between divergent genomes. While an analysis at the gene level is interesting, we reasoned that it makes more sense to look at the conservation of individual exons within the gene. The region-level visualizations indeed show that conservation is higher at the exon level than at the gene level, i.e. some exons may be missing, making the gene less conserved, while individual exons are conserved.

The ability to give each cluster a *P* value is an important contribution. However, the null distribution assumes that there is a completely random relationship between the organisms, which is not true. Currently, the τ parameter, representing a lower bound on their conservation ratio (in terms of the ratio of conserved and non-conserved exons) is used to regulate the clusters. Future contributions could develop a null model which takes into account evolution between two species. For example, a better permutation may shuffle groups of exons (genes), rather than individual exons. Yet as indicated earlier, this will give rise to computational difficulties.

Another important consideration is that exons which do not have a hit with the other organism increase the distance between hits when constructing the dendrogram, but they do not penalize the cluster score. Again, this was a deliberate choice to be less sensitive to evolutionary insertions and deletions but could be changed by using a different distance measure.

Altogether, Proteny is a powerful tool which can detect synteny between relatively divergent genomes at the amino acid sequence level. It detects clusters of exons based on a significance test and provides a rich visualization which supports the interpretation of the detected syntenic regions.

## Supplementary Material

Supplementary Data

## References

[btv389-B1] Altschul S.F. (1997) Gapped BLAST and PSI-BLAST: a new generation of protein database search programs. Nucleic Acids Res., 25, 3389–3402. 9254694 10.1093/nar/25.17.3389 PMC146917

[btv389-B2] Altschul S.F. Lipman D.J. (1989) Trees, stars, and multiple biological sequence alignment. SIAM J. Appl. Math., 49, 197–209.

[btv389-B3] Angiuoli S.V. Salzberg S.L. (2011) Mugsy: fast multiple alignment of closely related whole genomes. Bioinformatics, 27, 334–342. 21148543 10.1093/bioinformatics/btq665 PMC3031037

[btv389-B4] Arnaud M.B. (2010) The Aspergillus Genome Database, a curated comparative genomics resource for gene, protein and sequence information for the *Aspergillus* research community. Nucleic Acids Res., 38(Database Issue), D420–D427. 19773420 10.1093/nar/gkp751 PMC2808984

[btv389-B5] Baker A.R. (2013) Genome sequence of the button mushroom *Agaricus bisporus* reveals mechanisms governing adaptation to a humic-rich ecological niche. Proc. Natl. Acad. Sci. USA, 110, 4146. 10.1073/pnas.1206847109 PMC3491501 23045686

[btv389-B6] Blanchette M. (2004) Aligning multiple genomic sequences with the threaded blockset aligner. Genome Res., 14, 708–715. 15060014 10.1101/gr.1933104 PMC383317

[btv389-B7] Byrne K.P. Wolfe K.H. (2005) The Yeast Gene Order Browser: combining curated homology and syntenic context reveals gene fate in polyploid species. Genome Res., 15, 1456–1461. 16169922 10.1101/gr.3672305 PMC1240090

[btv389-B8] Crešnar B. Petrič S. (2011) Cytochrome P450 enzymes in the fungal kingdom. Biochim. Biophys. Acta, 1814, 29–35. 20619366 10.1016/j.bbapap.2010.06.020

[btv389-B9] Darling A.C.E. (2004) Mauve: multiple alignment of conserved genomic sequence with rearrangements. Genome Res., 14, 1394–1403. 15231754 10.1101/gr.2289704 PMC442156

[btv389-B10] Ghiurcuta C.G. Moret B.M.E. (2014) Evaluating synteny for improved comparative studies. Bioinformatics, 30, i9–i18. 24932010 10.1093/bioinformatics/btu259 PMC4058928

[btv389-B11] Goeman J.J. Finos L. (2012) The inheritance procedure: multiple testing of tree-structured hypotheses. Stat. Appl. Genet. Mol. Biol., 11, Article 11. 22499687 10.1515/1544-6115.1554

[btv389-B12] Grigoriev I.V. (2012) The genome portal of the Department of Energy Joint Genome Institute. Nucleic Acids Res., 40(Database issue), D26–D32. 22110030 10.1093/nar/gkr947 PMC3245080

[btv389-B13] Hulsman M. (2013) Ibidas: querying flexible data structures to explore heterogeneous bioinformatics data. In: Baker C.J.O. (eds.) Data Integration in the Life Sciences, Springer, Berlin Heidelberg, pp. 23–37.

[btv389-B14] Husemann P. Stoye J. (2010) R2Cat: synteny plots and comparative assembly. Bioinformatics, 26, 570–571. 20015948 10.1093/bioinformatics/btp690 PMC2820676

[btv389-B15] Jahn K. (2013) Statistics for approximate gene clusters. BMC Bioinformatics, 14(Suppl. 15), S14. 24564620 10.1186/1471-2105-14-S15-S14 PMC3908651

[btv389-B16] Knijnenburg T.A. (2009) Fewer permutations, more accurate P-values. Bioinformatics, 25, i161–i168. 19477983 10.1093/bioinformatics/btp211 PMC2687965

[btv389-B17] Krzywinski M. (2009) Circos: an information aesthetic for comparative genomics. Genome Res., 19, 1639–1645. 19541911 10.1101/gr.092759.109 PMC2752132

[btv389-B18] Langfelder P. (2008) Defining clusters from a hierarchical cluster tree: the Dynamic Tree Cut package for R. Bioinformatics, 24, 719–720. 18024473 10.1093/bioinformatics/btm563

[btv389-B19] Long M. (2003) The origin of new genes: glimpses from the young and old. Nat. Rev. Genet., 4, 865–875. 14634634 10.1038/nrg1204

[btv389-B20] Mason M.J. (2009) Signed weighted gene co-expression network analysis of transcriptional regulation in murine embryonic stem cells. BMC Genomics, 10, 327. 19619308 10.1186/1471-2164-10-327 PMC2727539

[btv389-B21] McClean P.E. (2010) Synteny mapping between common bean and soybean reveals extensive blocks of shared loci. BMC Genomics, 11, 184. 20298570 10.1186/1471-2164-11-184 PMC2851600

[btv389-B22] Minkin I. (2013) Sibelia: a scalable and comprehensive synteny block generation tool for closely related microbial genomes. In: Aaron D. Jens S. (eds.) Proceedings of the 13th Workshop Algorithms in Bioinformatics (WABI'13), Vol. 8126 of Lecture Notes in Computer Science pp. 215–229. Springer Verlag, Berlin.

[btv389-B23] Ohm R.A. (2010) Genome sequence of the model mushroom *Schizophyllum commune*. Nat. Biotechnol., 28, 957–963. 20622885 10.1038/nbt.1643

[btv389-B24] Ohm R.A. (2012) The blue light receptor complex WC-1/2 of *Schizophyllum commune* is involved in mushroom formation and protection against phototoxicity. Environ. Microbiol., 15, 943–955. 22998561 10.1111/j.1462-2920.2012.02878.x

[btv389-B25] Overbeek R. (1999) Use of contiguity on the chromosome to predict functional coupling. In Silico Biol., 1, 93–108. 11471247

[btv389-B26] Pearson W.R. (1998) Empirical statistical estimates for sequence similarity searches. J. Mol. Biol., 276, 71–84. 9514730 10.1006/jmbi.1997.1525

[btv389-B27] Pel H.J. (2007) Genome sequencing and analysis of the versatile cell factory *Aspergillus niger* CBS 513.88. Nat. Biotechnol., 25, 221–231. 17259976 10.1038/nbt1282

[btv389-B28] Proost S. (2012) i-ADHoRe 3.0–fast and sensitive detection of genomic homology in extremely large data sets. Nucleic Acids Res., 40, e11. 22102584 10.1093/nar/gkr955 PMC3258164

[btv389-B29] Shaw C.D. (2008) Genomic spring-synteny visualization with IMAS. In: Moore C. (eds.) 2008 Fifth International Conference BioMedical Visualization: Information Visualization in Medical and Biomedical Informatics, IEEE Computer Society, pp. 3–8.

[btv389-B30] Simillion C. (2008) i-ADHoRe 2.0: an improved tool to detect degenerated genomic homology using genomic profiles. Bioinformatics, 24, 127–128. 17947255 10.1093/bioinformatics/btm449

[btv389-B31] Sinha A.U. Meller J. (2007) Cinteny: flexible analysis and visualization of synteny and genome rearrangements in multiple organisms. BMC Bioinformatics, 8, 82. 17343765 10.1186/1471-2105-8-82 PMC1821339

[btv389-B32] Soderlund C. (2011) SyMAP v3.4: a turnkey synteny system with application to plant genomes. Nucleic Acids Res., 39, e68. 21398631 10.1093/nar/gkr123 PMC3105427

[btv389-B33] Sullivan M.J. (2011) Easyfig: a genome comparison visualizer. Bioinformatics, 27, 1009–1010. 21278367 10.1093/bioinformatics/btr039 PMC3065679

[btv389-B34] Vallenet D. (2006) MaGe: a microbial genome annotation system supported by synteny results. Nucleic Acids Res., 34, 53–65. 16407324 10.1093/nar/gkj406 PMC1326237

[btv389-B35] Vandepoele K. (2002) The automatic detection of homologous regions (ADHoRe) and its application to microcolinearity between *Arabidopsis* and rice. Genome Res., 12, 1792–1801. 12421767 10.1101/gr.400202 PMC187543

[btv389-B36] Yekutieli D. (2008) Hierarchical false discovery rate controlling methodology. J. Am. Stat. Assoc., 103, 309–316.

[btv389-B37] Zeng X. (2008) OrthoCluster: a new tool for mining synteny blocks and applications in comparative genomics. In: Proceedings of the 11th International Conference on Extending Database Technology: Advances in Database Technology, ACM New York, NY, USA, pp. 656–667.

