# Supplementary material for: Proteny: discovering and visualizing statistically significant syntenic clusters at the proteome level
Source: Bioinformatics. 2015 Jun 23;31(21):3437–44. doi: 10.1093/bioinformatics/btv389 (PMC4612220; doi:10.1093/bioinformatics/btv389)
Supplement: Supplementary Data [file supp_31_21_3437__index.html]

Proteny: discovering and visualizing statistically significant syntenic clusters at the proteome level — Proteny: discovering and visualizing statistically significant syntenic clusters at the proteome level — Supplementary Data 

# Proteny: discovering and visualizing statistically significant syntenic clusters at the proteome level

## Supplementary Data

files

- Supplementary Data - pdf file
