# Supplementary material for: Proteny: discovering and visualizing statistically significant syntenic clusters at the proteome level
Source: Bioinformatics. 2015 Jun 23;31(21):3437–44. doi: 10.1093/bioinformatics/btv389 (PMC4612220; doi:10.1093/bioinformatics/btv389)
Supplement: Supplementary Data [file supp_btv389_supp.pdf]

Supplement

Proteny: Discovering and visualizing statistically significant syntenic clusters at the proteome level

Thies Gehrmann[1], Marcel J. T. Reinders[1]

[1]Delft Bioinformatics Lab, Delft University of Technology, Mekelweg 4, 2628 CD Delft, The Netherlands

S-1 DIVERGENCE OF CODON USAGE BETWEEN *S. COMMUNE* AND *A. BISPORUS*

Synteny analysis at the nucleotide sequence level will not always yield interpretable results. For example, codon usage in the amino acid translation depends on the GC content of an organism (Ikemura, 1985): i.e. for GC-rich organisms, the third nucleotide (the wobble position) of a codon is generally a *guanine* or In fungi, GC contents are highly variable and it is often the case that codon usage is indeed quite different. For example, figure SF-1 shows the frequencies of codons in two different fungi, *Schizopyllum commune* and *Agaricus bisporus*, showing indeed considerable codon usage differences. Although this results in differences at the nucleotide sequence level, the protein sequences themselves are conserved. When making a mapping between genes, biologists routinely use the amino acid sequences of the associated proteins, because the nucleotide sequence do not result in a hit. Querying the genes from the two genomes against one another using nucleotide BLAST returns only 125 hits, while protein BLAST gives almost 200000 hits. This clearly shows that in situations like these, synteny should be established on the amino acid sequence level.

Figure SF-1 shows that the codon usage between the two Basidiomycetes are substantially different. Aligning these genomes will not work, due to the divergence between the genomes. A BLAST search between nucleotide sequences will rarely return any results. At the amino acid sequence level however, with a BLASTp, the sequence similarity can be recovered.

S-2 DOT MATRIX VISUALIZATION

Figure SF-2 shows a dot matrix visualization for the alignment for *Schizopyllum commune* and *Agaricus bisporus*. It was generated at http://genome.jgi.doe.gov/vista_embed/?organism=Schco2. It is clear that this visualization is not particularly useful. While there is little to see (because the figure was made from genome alignment), it also is hard to see exactly how the chromosomes relate to each other. Furthermore, it is not particularly helpful when viewing smaller regions simultaneously.

S-3 DISTANCE CALCULATIONS AND THE CLUSTER SCORE

Figure SF-3a describes the variables which define a hit, which are in turn used to calculate distances between hits (figures SF-3b-c.

The score for a cluster is based upon the score of the hits within the cluster, and the unaccounted exons the cluster encompasses.

S-4 ANALYTICAL DESCRIPTION OF THE NULL DISTRIBUTION

The cluster score is a summation of three different distributions. Since each part of the cluster score is a summation over random variables, we can invoke the central limit theorem for each component. The *matching* component, M, which represents the sum of the hits in the cluster, and the components for the unaccounted exons on each organism: U_χ We estimate a normal distribution for each component:

$$M^C \sim \mathcal{N}(n_C\mu_M, n_C\sigma^2_M) \tag{SE-1}$$

$$U^C_\chi \sim \mathcal{N}(n_{C,\chi}\mu_{U,\chi}, n_{C,\chi}\sigma^2_{U,\chi}) \tag{SE-2}$$

where each μ and σ^2 was estimated from the list of hits, and the list of best hits for each exon. In turn, we can model the cluster null score by summing these distributions:

$$s^C_{clt} = 2M^C - U^C_\beta - U^C_\gamma \tag{SE-3}$$

$$\mu^C_{clt} = 2n_C\mu_M - n_{C,\beta}\mu_{U,\beta} - n_{U_\gamma}\mu_{U,\gamma} \tag{SE-4}$$

$$\sigma^{2,C}_{clt} = 2^2 n_C\sigma^2_M + n_{U_{C,\beta}}\sigma^2_{U,\beta} + n_{U_{C,\gamma}}\sigma^2_{U,\gamma} \tag{SE-5}$$

$$s^C_{clt} \sim \mathcal{N}(\mu_{clt}, \sigma^{2,C}_{clt}) \; , \tag{SE-6}$$

which can then be used to calculate a p-value:

$$p^C_{clt}(C) = 1 - P(s_{clt} < s(C)) \tag{SE-7}$$

© Oxford University Press 2013.

S-4.1 Quality of approximation

We compare the CLT approximation to the permutation tests to determine if the CLT p-value is insigificant when the permutation p-value is. To do this, we perform 100,000 permutations for a set of over 6700 clusters (A whole tree), and compare the CLT approximation to the p-value from the permutation. Figure SF-4 plots the permutation values against the CLT approximation values. We observe that they are highly correlated, as expected, with a pearson correlation coefficient of 0.969582. In the logspace plot in figure SF-4b, we see an artifact from the permutations, whereby the resultion of a permuted p-value is limited by the number of permutations. The vertical stripes towards the smaller p-values are where the p-values are no longer discernible from each other. This makes the log-space slightly less correlated, at 0.944636.

The separate band that is seen in figure SF-4a are the leaf nodes (clusters containing only a single hit), and due to our normal approximation, fall along the normal cumulative distribution function curve. As we increase the minimum size of cluster which we approximate with the CLT, we get a better approximation of the permutation test, but as a consequence, we have to perform more permutations. Table ST-1 shows this effect, and figures SF-4c and SF-4d show the individual points for clusters of at least 10 elements in each component. In all our experiments, we only calculated p-values for a cluster if the size of each component was less than 10 (i.e. $|C| > 10$, $|U_1| > 10$ and $|U_2| > 10$).

S-5 A DYNAMIC CUTTING ALGORITHM

We prefer a child node over its parent node if it is more significant than its parent and fulfills the conservation ratio, *or*, the parent does not fulfill the conservation ratio *or* if the parent is not significant. We express this in a recursive function, *cut*:

$$cut(n) := \begin{cases} cut(n_L) \;\cup\; cut(n_R) & \textbf{if} \begin{cases} [p(C_R) >= p(C_n) \wedge \tau_{C_R} > \tau] & \textbf{or} \\ [p(C_L) >= p(C_n) \wedge \tau_{C_L} > \tau] & \textbf{or} \\ [p(C_n) > \alpha \vee \tau_{C_n} > \tau] \end{cases} \\ \emptyset & \textbf{if}\, n_C = \emptyset \\ \{C_n\} & \textbf{if}\, p(C_n) > \alpha \wedge \tau_{C_n} > \tau \\ \{\} & \textbf{otherwise} \end{cases} \quad \text{(SE-8)}$$

where n is a node in the dendrogram, C_n is the cluster described by node n, n_L and n_R are the child nodes of node n, $p(\cdot)$ is the p-value for a given cluster, α is a threshold for the p-value, $\tau_x = \frac{n_x+1}{n_{x,\beta}+n_{x,\gamma}+1}$, the ratio of conservation for a cluster x, and τ is the conservation ratio threshold.

This is of course a greedy algorithm; There may be child nodes still *more* significant deeper in the tree. The most significant nodes can only be found by an exhaustive search, incurring a costly correction factor. The dynamic cut makes a compromise between the number of tests, and the minimality of the p-values.

S-6 IMPLEMENTATION DETAILS

Proteny is implemented in Python using the Ibidas (Hulsman *et al.*, 2013) data query and manipulation suite. The BLASTp utility from the blast+ tools suite serves as the local aligner. Finally, the Circos (Krzywinski *et al.*, 2009) utility is used to visualize the discovered clusters. Proteny requires as input a tab-separated file describing gene locations, and the genome in FASTA Format.

S-7 I-ADHORE CLUSTERS

In order to obtain i-ADHoRe clusters, we performed a BLASTp query between all proteins in each organism, and supplied these to i-ADHoRe. For this, we use the same BLASTp parameters that were used in Proteny. The parameter settings for the *A. niger* strains were based on the suggested parameters from the yeast dataset from the i-ADHoRe publication, and a correspondence with the authors. The settings used are given in table ST-3. In general, we found that the results for the *A. niger* dataset were not very sensitive to the parameters (probably due to high conservation). However, for the basidiomycota, they were more sensitive. Clusters became larger with more gaps, such that the relationships seen in figure SF-10 did not change.

By visualizing the i-ADHoRe clusters in the same way as Proteny, we have another way to easily compare the results from Proteny and i-ADHoRe. We find that i-adhore has many more clusters which are quite large, spanning large areas. This often makes the visualizations hard to interpret. However, many of the large structures we discover are also present in the results of i-ADHoRe. Presented here are the same chromosome-level visualizations presented in the paper, except instead the i-ADHoRe clusters are shown.

Additionally, study the differences between the Proteny and i-ADHoRe visualizations for an apparent scaffolding error in figure SF-7.

S-8 GUIDING A GENOME ASSEMBLY

We provide here figures which support the ability of Proteny to help identify errors in a genome assembly by comparing two assemblies. Figure SF-7 shows three chromosomes in n402 which can be fused together, but, probably due to an assembly limitation, were not.

S-9 DEVELOPMENTAL PROTEIN CLUSTERS

We are particularly interested in eight transcription factors which are known to be involved in mushroom formation, and a light sensing protein which has been linked to the initiation of mushroom formation in *S. commune*. To increase the confidence that these transcription factors are functionally similar in both *S. commune* and *A. bisporus*, we wish to find that these genes lie in syntenic regions.

Proteny reveals that six of these nine developmental proteins lie within conserved clusters. For example, figure SF-8c shows the region-level plot for the cluster which contains the transcription factor *gat1*. The figure clearly shows that the transcription factor lies in a well conserved region, i.e. neighboring genes in *A. bisporus* match to neighboring genes in *S. commune*. Despite the order between genes not being conserved, and not all exons being conserved within their homolog, Proteny still can still call a cluster here. Figure SF-8 shows the region-level plots for the clusters of the other developmental proteins found in syntenic clusters. The clusters found for proteins *bri1*, *fst4*, and *wc2* are exceedingly large, and rather than show the entire cluster, we show here only a sub-region of the larger cluster which shows only the 30,000bp up- and downstream regions of the developmental protein and it's homolog.

For the developmental proteins which are not found in clusters, we plot the blast hits in the 30,000bp up and downstream regions of the gene and its homolog in figure SF-9. In these figures it becomes clear why they were not found in syntenic clusters.

S-10 CLUSTERS DISCOVERED BY PROTENY AND I-ADHORE

We present here tables describing the discovered clusters. Table ST-4 shows the features of the clusters discovered Proteny for the *Aspergillus niger*, and table ST-5 lists the same for the basidiomycota. To compare, we also calculate the scores for clusters discovered by i-ADHoRe using our scoring metrics, and calculate p-values. The correction factor is defined by the number of clusters in the dataset, i.e. 189 and 377 for the *A. niger* and basidiomycota experiments respectively. These are shown in tables ST-6 and ST-7.

We compare these datasets with some graphs in figure SF-10.

The seemingly identical p-values from Proteny are an artifact of the permutation tests; since we do the number of permutations required by the correction factor, we will often arrive at the same p-value. Some p-values are zero. This comes from a floating point limitation from within the CLT approximation. To overcome this, we use the z-score internally to compare clusters.

S-10.1 Reconstruction of genome rearrangement between n402 and CBS513.88

To aid the understanding of the rearrangement seen in figure 3, figure SF-11 gives a schematic reconstruction. The clusters are drawn as shown in figure 3, but on a linear plane. Here we see that, while scaffolds 5,6 and 7 from n402 may not have been joined due to an error in the assembly. It becomes clear that scaffold 5 on CBS513.88 must have split in order to facilitate this alignment. In order to discount this rearrangement, we must assume 3 errors in the assembly of n402 and CBS513.88: 1. scaffold 12 in CBS513.88 incorrectly joins sections 12B and 12C, 2. scaffold 5 in n402 incorrectly joins sections 5A and 5B, and 3. scaffold 6 in n402 incorrectly joins sections 6A and 6B. The probability of these three errors at exactly these three locations all being true is undoubtedly low. Based on the syntenic clusters discovered, we trust that this rearrangement is a more reasonable explanation.

S-11 THE YEAST GENE ORDER BROWSER DATASET

Proteny and i-ADHoRe are run on the same dataset as in (Ghiurcuta and Moret, 2014), on the following genomes:

Genome	Number of genes	Genes with orthology statements in YGOB
C. glabrata	5461	5450
E. gossypii	4959	4945
K. lactis	5318	5291
L. kluyveri	5683	5663
L. thermotolerans	5425	5401
L. waltii	11,275	5522
S. cerevisiae	7016	5987
Z. rouxii	5338	5323

Most of these organisms do not contain very many exons, and most have single exon genes. This means that Proteny's strategy of using exon sequence similarities is not exploited in this dataset.

S-11.1 Processing the YGOB dataset

We downloaded the genomes and gene descriptions for the above mentioned genomes from the YGOB, together with the orthology statement (pillars) file. The sequence IDS in the fasta files did not correspond to the sequence IDS in the gene descriptions file, so these were manually corrected. We converted the YGOB formatted gene description file into GFF format and from here they were converted into the specific formats required by Proteny and i-ADHoRe.

Some genomes had two columns in the Pillars file, as ohnolog descriptions. For each other genome, if there was a gene which had two homology statements from the same genome, we counted this as two homologs for this gene.

S-11.2 Results

Table ST-8 shows the number of clusters found for each pairwise comparison by Proteny and i-ADHoRe, respectively. We find that in $18/28$ pairwise comparisons, that Proteny finds fewer clusters, and in total, Proteny finds 3668 clusters, while i-ADHoRe finds 5996 clusters. We find that Proteny and i-ADHoRe find these clusters covering comparable numbers of genes (given in table ST-9). Sometimes Proteny covers more genes, while sometimes i-ADHoRe covers more. For both Proteny and i-ADHoRe, these counts include genes which do not have curated homology statements.

Using the relaxed score from (Ghiurcuta and Moret, 2014), we study the distributions of these scores from Proteny and i-ADHoRe in figure SF-12. We find that the clusters of Proteny have a relaxed score distribution which is higher than that of i-ADHoRe. Figure SF-12a shows that, for each pairwise test, the average relaxed cluster score is generally higher for Proteny than for i-ADHoRe. This is a general trend; We see in figure SF-12b, that when looking at all scores from all tests simultaneously, the distribution of scores is higher in Proteny than in i-ADHoRe. It is not a rule, as seen in figure SF-12c, there are cases for which i-ADHoRe does perform better, and achieves better cluster scores, this can also be seen in figure SF-12a, where some points lie below the red line, indicating that the means of Proteny are lower than the means of i-ADHoRe. However, in these cases, the distributions are usually not statistically significantly different ($q > 0.5$) by a 2 sample Kolmogorov-Smirnov test, with bonferroni multiple testing correction. This indicates that Proteny performs better than i-ADHoRe on this test set.

S-12 RUNNING TIME

We provide an example of the running time of Proteny. We neglect the time for BLASTp, which is a necessary step for almost all synteny discovery algorithms. A complete run of the example provided together with the implementation, between the yeast genomes *C. glabrata* and *Z. rouxii* takes about 10 minutes. The construction of the distance matrix takes 105 seconds. Building the dendrogram takes 0.7 seconds. Cutting the dendrogram takes 533 seconds. However, Proteny is not optimized beyond some parallelization during permutation calculations, allowing for the possibility of significant speed-up.

REFERENCES

Ghiurcuta, C. G. and Moret, B. M. E. (2014). Evaluating synteny for improved comparative studies. *Bioinformatics (Oxford, England)*, **30**(12), i9–18.

Hulsman, M., Bot, J. J., de Vries, A. P., and Reinders, M. J. T. (2013). Ibidas: Querying Flexible Data Structures to Explore Heterogeneous Bioinformatics Data. *Data Integration in the Life Sciences*, pages 23–37.

Ikemura, T. (1985). Codon usage and tRNA content in unicellular and multicellular organisms. *Molecular biology and evolution*, **2**(1), 13–34.

Krzywinski, M., Schein, J., Birol, I., Connors, J., Gascoyne, R., Horsman, D., Jones, S. J., and Marra, M. a. (2009). Circos: an information aesthetic for comparative genomics. *Genome research*, **19**(9), 1639–45.

FIGURES

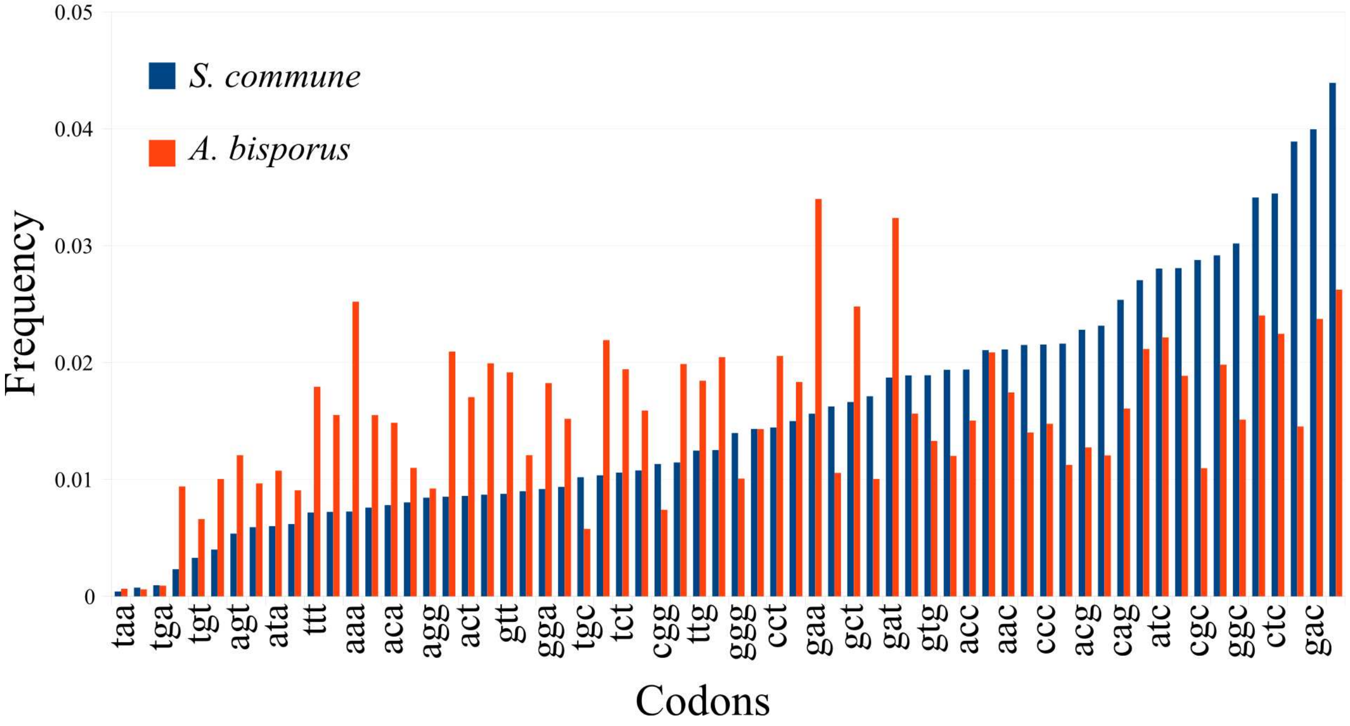

Fig. SF-1: *Comparing codon usage in Schizopyllum commune and Agaricus bisporus. The plot relates the frequencies of each codon in S. commune and A. bisporus. We observe that the codon usage frequencies are largly different. They are not correlated ($r = 0.454$). An analysis on the nucleotide sequence level is not likely to result in interpretable results, due to the differences in codon usages. Their protein sequences are conserved, however, and form the basis for Proteny.*

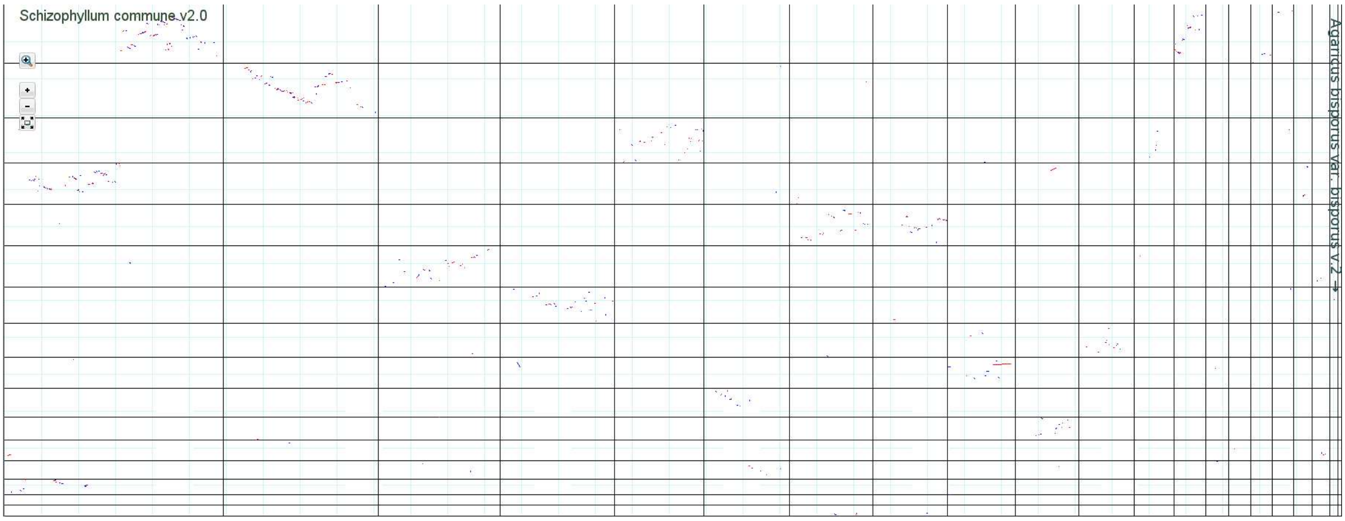

Fig. SF-2: *The Dot Matrix visualization for A. bisporus and S. commune is difficult to examine, and provides little information.*

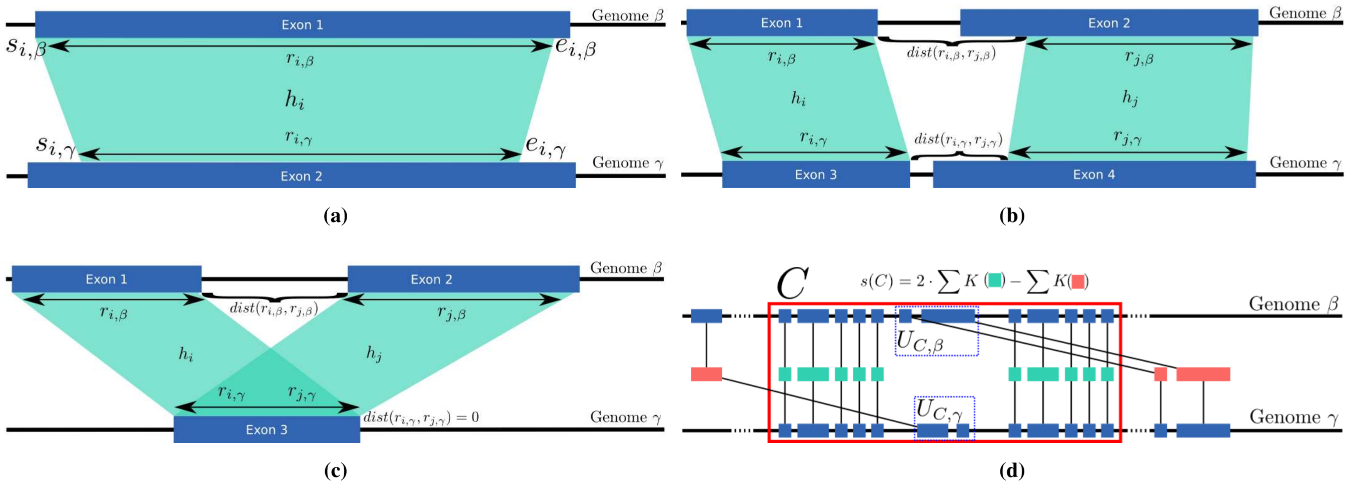

Fig. SF-3: ***(a)*** *A visual representation of the variables involved in the distance calculations.* ***(b)*** *The distances between two hits. In this example, the distance between the two hits would be $dist(r_{i,\beta}, r_{j,\beta}) + dist(r_{i,\gamma}, r_{j,\gamma})$.* ***(c)*** *The distance between two hits sharing an exon. In this example, the distance between the two hits would simply be $dist(r_{i,\beta}, r_{j,\beta})$. Both hits fall on overlapping regions in genome γ.* ***(d)*** *Illustration of the calculation of the score. Unaccounted exons in $U_{C,\beta}$ and $U_{C,\gamma}$ penalize the score of the cluster.*

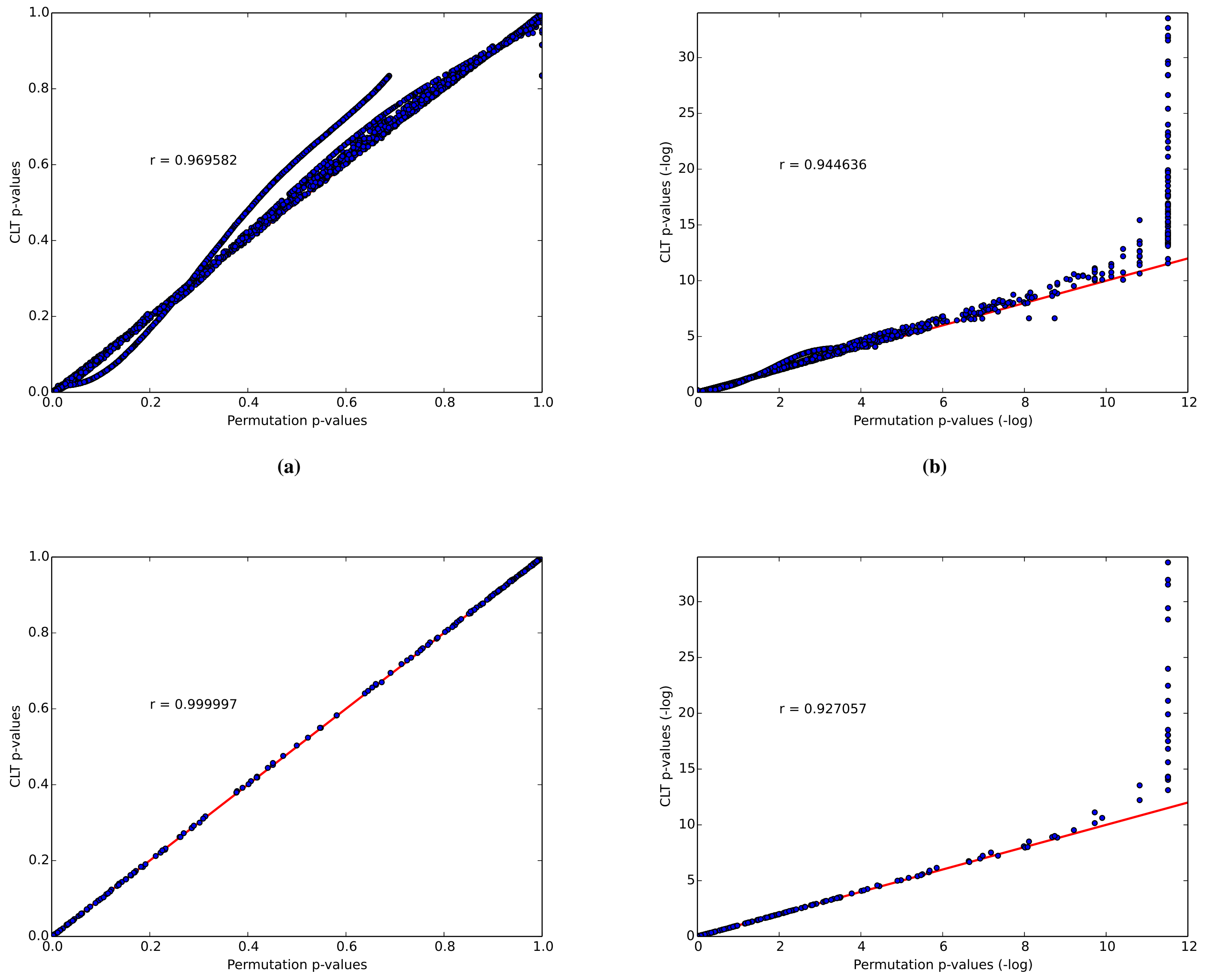

(a) **(b)** **(c)** **(d)**

Fig. SF-4: *(**a**): Permutation p-values compared to CLT approximation p-values, for all clusters. (**b**): Permutation p-values compared to CLT approximation p-values in -log space, for all clusters. (**c**): Permutation p-values compared to CLT approximation p-values, only for clusters which have at least 10 hits in each component. (**d**): Permutation p-values compared to CLT approximation p-values in -log space, only for clusters which have at least 10 hits in each component.*

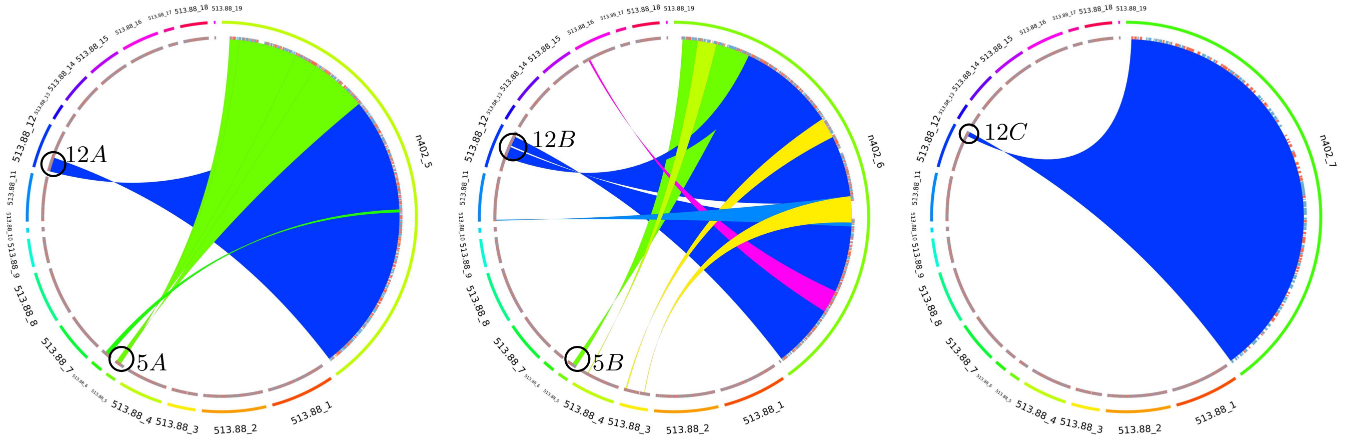

(a) (b) (c)

Fig. SF-5: ***(a)*** *Syntenic clusters found by i-ADHoRe for scaffold 5,* ***(b)*** *scaffold 6, and* ***(c)*** *scaffold 7 in n402.*

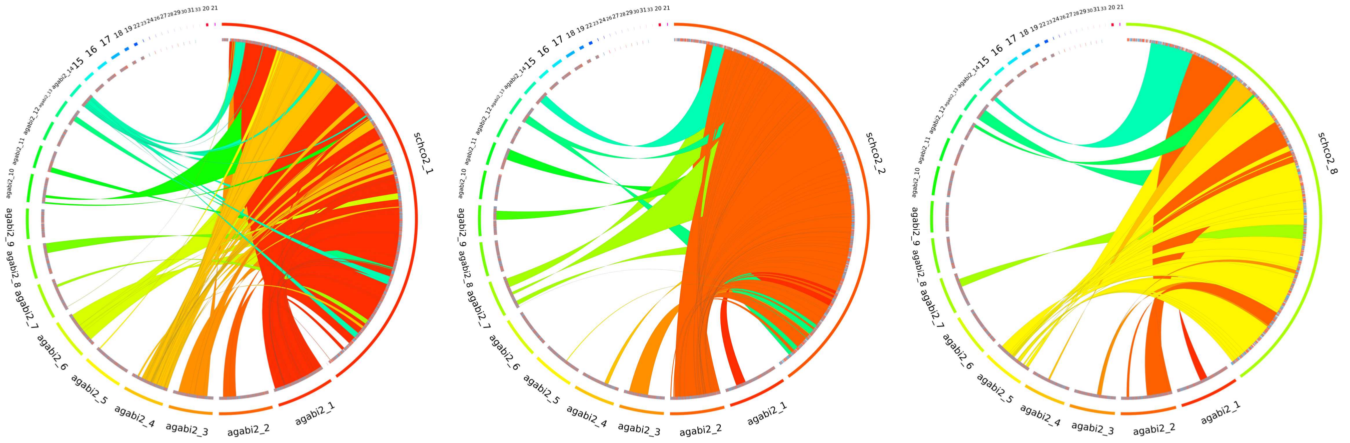

(a) (b) (c)

Fig. SF-6: ***(a)*** *Syntenic clusters found by i-ADHoRe for scaffold 1,* ***(b)*** *scaffold 2, and* ***(c)*** *scaffold 8 in S. commune.*

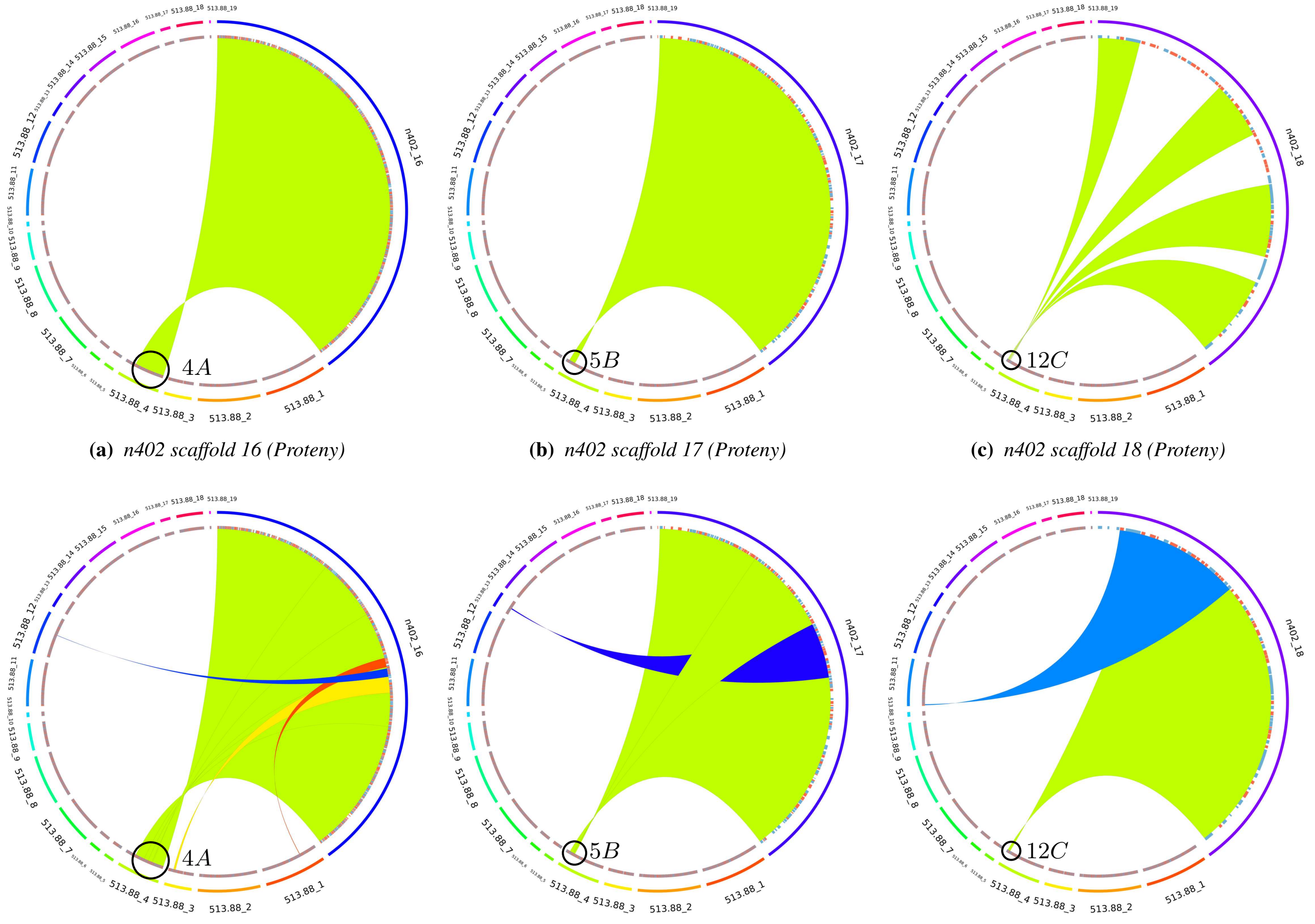

(a) *n402 scaffold 16 (Proteny)* **(b)** *n402 scaffold 17 (Proteny)* **(c)** *n402 scaffold 18 (Proteny)*

(d) *n402 scaffold 16 (i-ADHoRe)* **(e)** *n402 scaffold 17 (i-ADHoRe)* **(f)** *n402 scaffold 18 (i-ADHoRe)*

Fig. SF-7: ***(a)*** *Syntenic clusters found for scaffold 16,* ***(b)*** *scaffold 17, and* ***(c)*** *scaffold 18 in n402. From the clusters found, one can surmise that the chromosomes in n402 could be merged.* ***(d)*** *Syntenic clusters found by i-ADHoRe for scaffold 16,* ***(e)*** *scaffold 17, and* ***(f)*** *scaffold 18 in n402.*

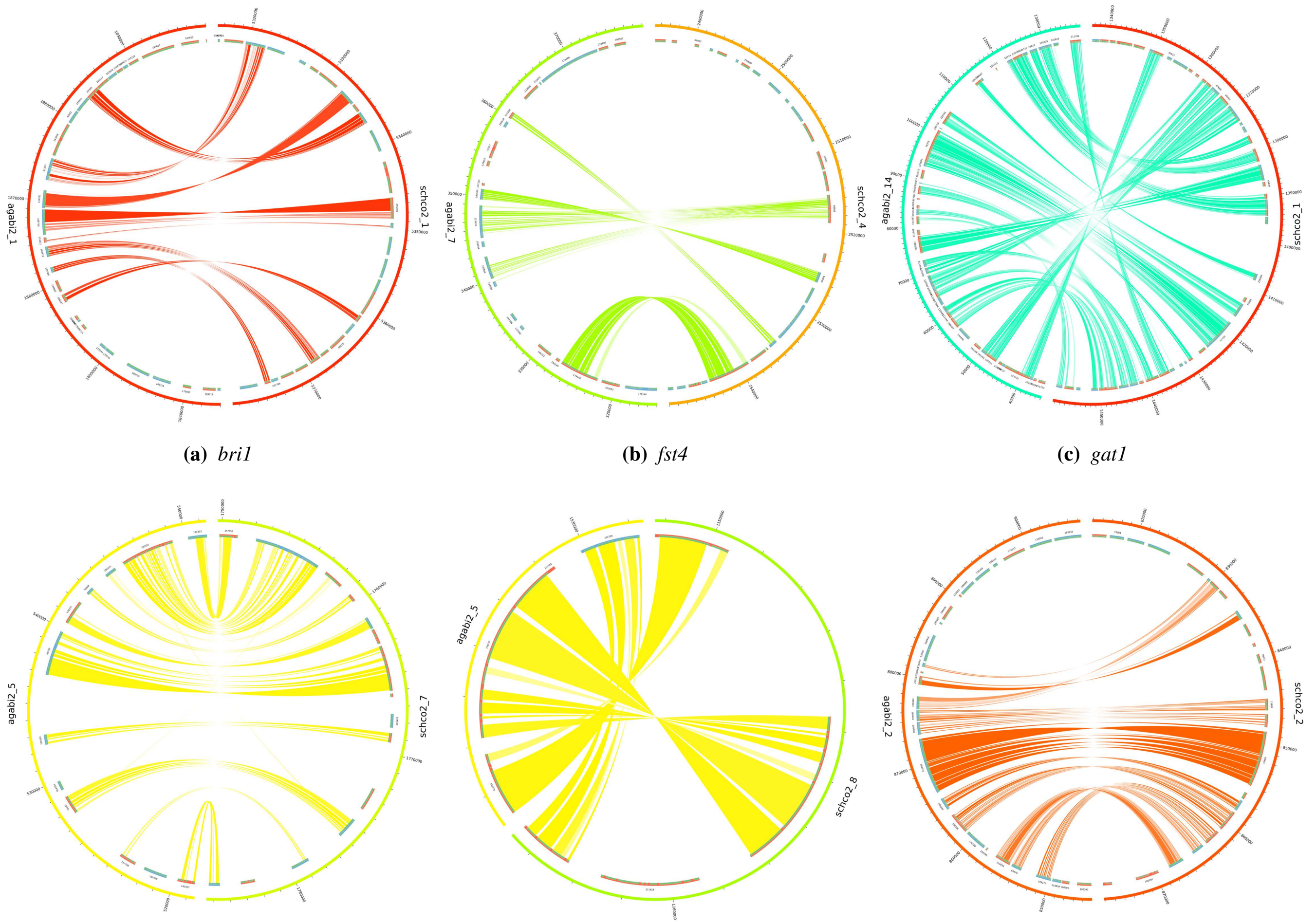

(a) *bri1*
(b) *fst4*
(c) *gat1*
(d) *hom1*
(e) *hom2*
(f) *wc2*

Fig. SF-8: *The clusters for the six developmental proteins which are found within clusters.*

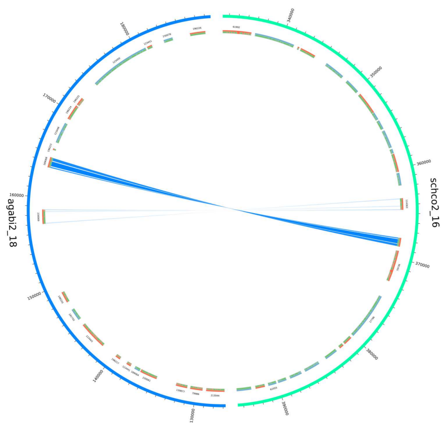

(a) *c2h2*

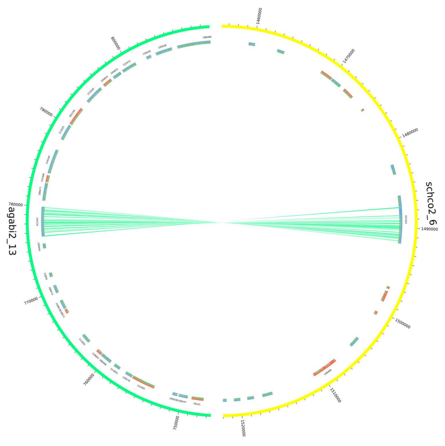

(b) *fst3*

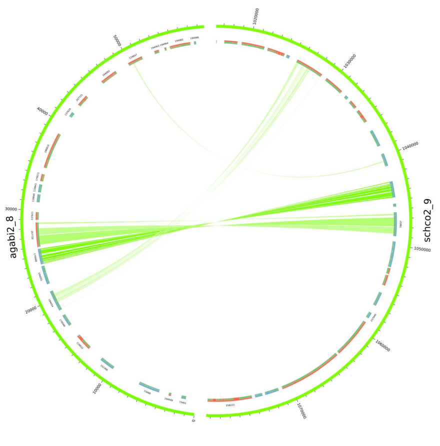

(c) *wc1*

Fig. SF-9: *The* $30{,}000bb$ *regions surrounding the developmental proteins not found in a cluster.*

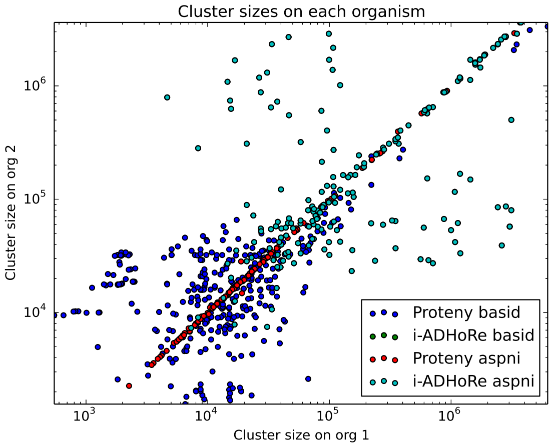

(a)

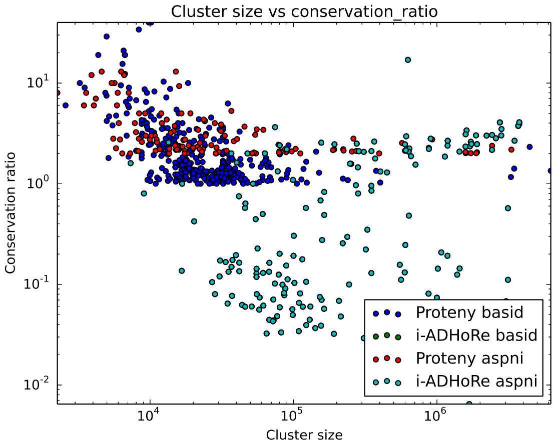

(b)

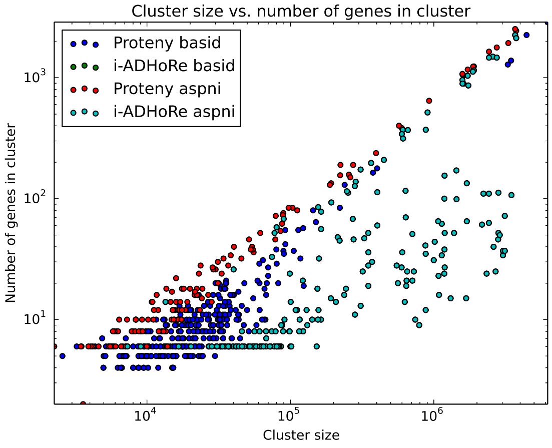

(c)

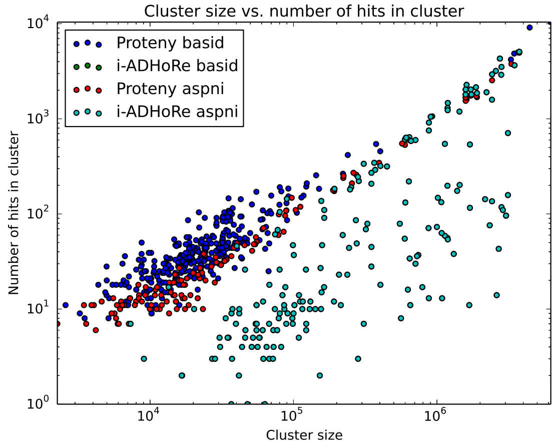

(d)

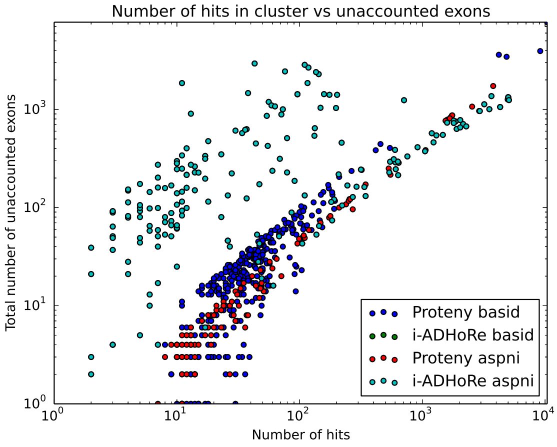

(e)

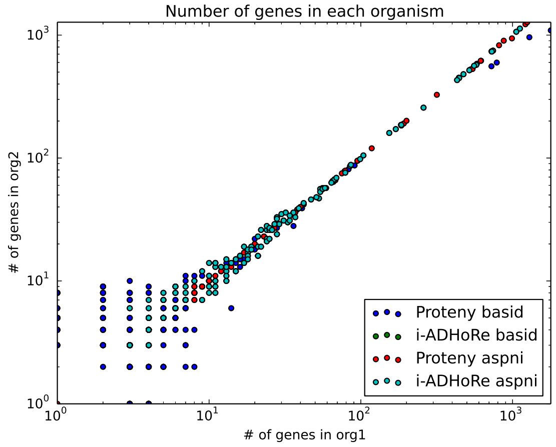

(f)

Fig. SF-10: ***Features of the datasets***: ***(a)***: *The cluster sizes on each end of the clusters.* ***(b)***: *The size of each cluster vs. its conservation ratio.* ***(c)***: *The size of each cluster vs. the number of genes in the cluster.* ***(d)***: *The size of each cluster vs. the number of hits in the cluster.* ***(e)***: *The number of hits vs. the number of unaccounted exons in the cluster.* ***(f)***: *The number of genes on each end of the cluster.*

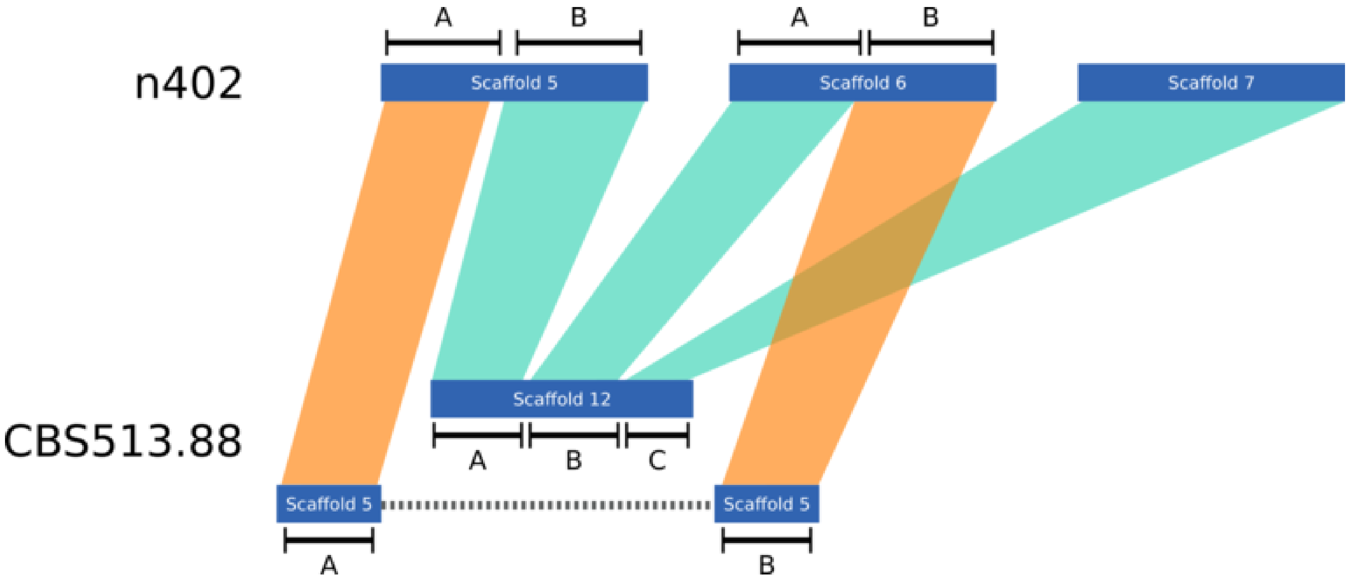

Fig. SF-11: *A reconstruction of the possible rearrangement that ocurred between n402 and CBS513.88.*

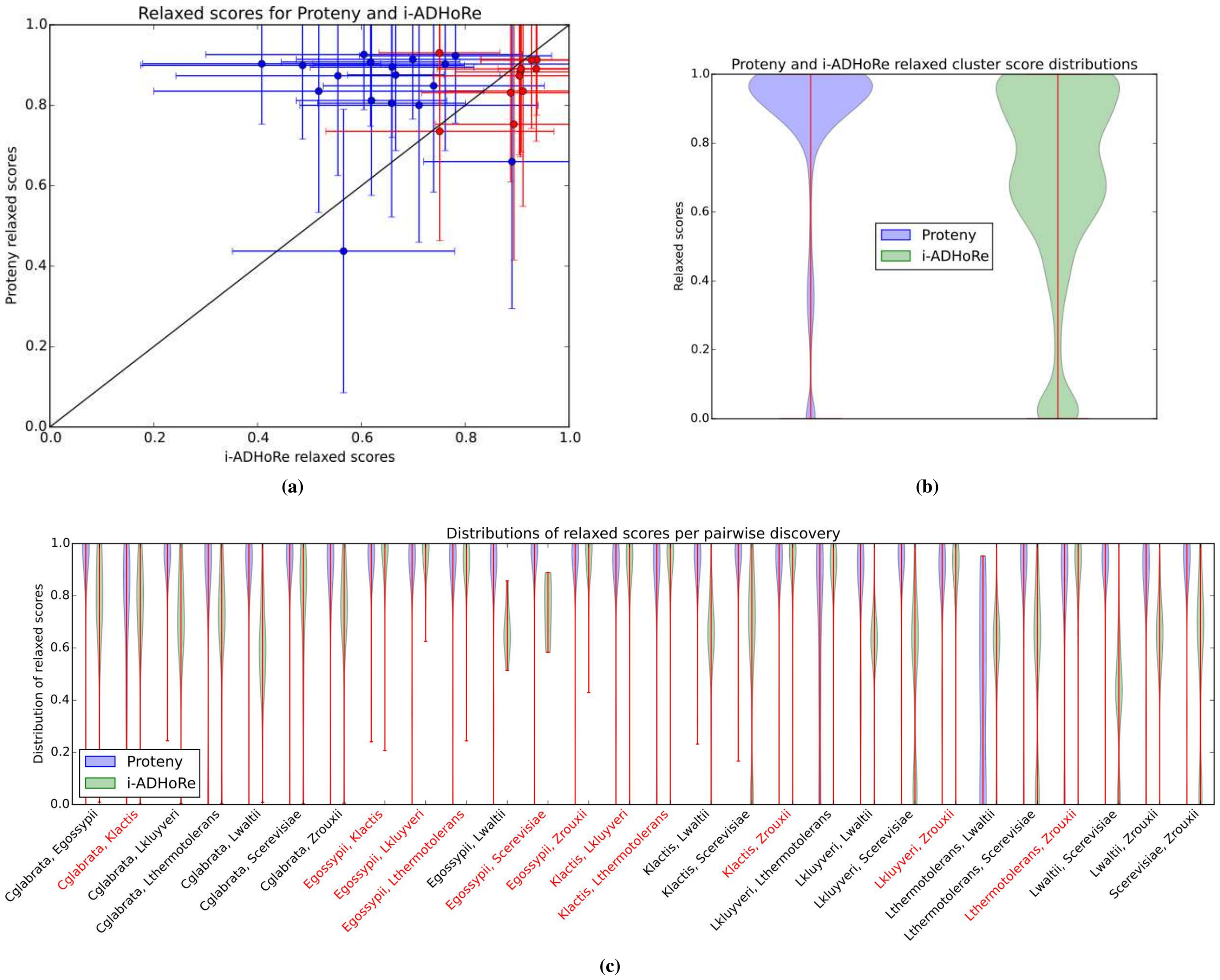

Fig. SF-12: ***Relaxed cluster scores****:* ***(a)*** *The means and their standard deviations of relaxed scores for Proteny and i-ADHoRe clusters for each pairwise test. Red points are cases where the relaxed score distributions are not significantly different between Proteny and i-ADHoRe (where significance requires* $q < 0.5$ *by a Kolmogorov-Smirnov test with bonferroni multiple testing correction).* ***(b)*** *The distributions of all relaxed scores for Proteny and i-ADHoRe.* ***(c)*** *The distributions of relaxed scores for Proteny and i-ADHoRe for each pairwise synteny discovery task. Tasks which do not have statistically significantly different score distributions are given a red title.*

TABLES

Minimum cluster size	Number of remaining clusters	Correlation	Correlation in $-\log$
0	6712	0.96958178800427153	0.944635871602162
1	1919	0.99994293156633374	0.93910797512522992
2	1813	0.99997089153420382	0.93969939620334619
3	1482	0.99998372142836534	0.94122390044238691
4	1275	0.99998890070405433	0.93883650832503363
5	1118	0.99999243272163818	0.9368089615041757
6	995	0.99999333088799836	0.93285516155764059
7	900	0.99999465306718771	0.93330324737035997
8	824	0.99999528695141415	0.9311538629486954
9	767	0.99999651760224839	0.93350933122001845
10	719	0.99999671365570508	0.92705664196612469

Table ST-1. Varying the minimum cluster size gives us different qualities of approximations.

	Total genes	# Scaffolds	Discovered genes<br>Proteny	 i-ADHoRe	 Overlap of Proteny and i-ADHoRe
A. niger			119 Clusters	189 Clusters (93 significant)	66.5%
n402	13,612	24	10,880	9667	9310
CBS513.88	14,067	19	10,959	9728	9343
Basidiomycota			345 Clusters	377 Clusters (250 significant)	41.4%
S. commune	14,652	36	5828	2588	2044
A. bisporus	10,438	31	4572	2552	1986

Table ST-2. An overview of the datasets and the results of the synteny analysis by Proteny and i-ADHoRe.

Setting	Value *A. niger*	Value *basidiomycota*
table_type	pairs	pairs
cluster_type	collinear	collinear
gap_size	30	30
cloud_gap_size	20	20
cloud_cluster_gap	25	25
cloud_filter_method	binomial	binomial
max_gaps_in_alignment	35	35
cluster_gap	35	35
tandem_gap	17	17
q_value	0.75	0.75
prob_cutoff	0.01	0.01
anchor_points	3	3
alignment_method	nw	n2
level_2_only	false	false
multiple_hypothesis_correction	FDR	FDR

Table ST-3. The options provided to i-ADHoRe.

Table ST-4: *Clusters discovered by Proteny for the aspergillus genomes*

	Scaffold		Cluster size (bp)			# Unaccounted exons		# Genes found			
**#**	**n402**	**CBS513.88**	**n402**	**CBS513.88**	**# Hits**	**n402**	**CBS513.88**	**n402**	**CBS513.88**	**score**	**p-value**
1	1	9	1575691	1585559	1546	304	463	533	528	2401.253	0.0
2	2	14	1722868	1722167	1753	385	486	582	585	2678.030	0.0
3	3	13	927223	904485	1063	156	229	317	327	1564.374	0.0
4	4	1	3752506	3625675	4967	585	679	1216	1226	6305.060	0.0
5	5	5	15178	15161	12	3	2	6	6	19.601	5.11205104692e-07

Continued on next page

Table ST-4 – continued from previous page

	Scaffold		Cluster size (bp)			# Unaccounted exons		# Genes found			
**#**	**n402**	**CBS513.88**	**n402**	**CBS513.88**	**# Hits**	**n402**	**CBS513.88**	**n402**	**CBS513.88**	**score**	**p-value**
6	5	5	18793	28257	19	2	2	7	7	28.390	5.11205104692e-07
7	5	5	15347	15360	10	2	2	5	5	18.877	5.11205104692e-07
8	5	5	3459	3450	11	0	1	3	3	21.743	5.11205104692e-07
9	5	5	3887	3907	11	0	0	3	3	21.350	5.11205104692e-07
10	5	5	8742	8774	12	0	4	4	4	22.022	5.11205104692e-07
11	5	5	40401	40001	49	6	11	20	20	91.729	5.11205104692e-07
12	5	5	14823	14947	23	3	6	8	9	41.476	5.11205104692e-07
13	5	5	15930	15959	27	0	2	11	11	52.150	5.11205104692e-07
14	5	5	52878	52944	48	7	16	19	19	82.015	5.11205104692e-07
15	5	12	18573	18568	11	1	3	6	6	19.301	5.11205104692e-07
16	5	12	17320	17514	10	0	3	6	6	19.314	5.11205104692e-07
17	5	12	45409	45530	47	7	5	16	16	86.112	5.11205104692e-07
18	5	12	26739	26742	28	4	7	8	8	54.291	5.11205104692e-07
19	5	12	51487	51292	41	5	15	23	23	75.852	5.11205104692e-07
20	5	12	10188	9708	11	1	2	5	5	20.854	5.11205104692e-07
21	5	12	6963	7262	13	3	3	5	5	21.557	5.11205104692e-07
22	5	12	13927	13849	15	0	4	3	3	24.309	5.11205104692e-07
23	5	12	17414	17411	13	2	4	5	5	20.337	5.11205104692e-07
24	5	12	11969	11969	14	0	2	6	6	27.083	5.11205104692e-07
25	5	12	9280	9331	17	2	3	4	4	32.480	5.11205104692e-07
26	5	12	36889	36864	36	1	5	14	14	65.023	5.11205104692e-07
27	5	12	6047	6053	7	1	0	3	3	13.810	5.11205104692e-07
28	5	12	6400	6337	11	2	3	5	5	17.648	5.11205104692e-07
29	5	12	15132	15130	12	0	0	6	6	23.165	5.11205104692e-07
30	5	12	11183	11186	17	4	2	6	6	32.622	5.11205104692e-07
31	5	12	261864	255195	271	31	65	75	75	411.003	0.0
32	6	5	255703	252385	210	43	57	79	79	365.588	0.0
33	6	12	79016	75994	99	25	23	36	36	177.846	0.0
34	6	12	103528	100752	110	12	37	42	42	184.415	0.0
35	6	12	222321	221609	239	53	55	78	78	437.708	0.0
36	6	12	78317	78510	60	7	17	23	23	100.607	5.11205104692e-07
37	6	12	7831	7754	11	1	1	4	4	21.106	5.11205104692e-07
38	6	12	4057	4057	11	0	1	3	3	21.492	5.11205104692e-07
39	6	12	22313	21647	27	3	7	8	8	47.388	5.11205104692e-07
40	6	12	571122	571422	550	68	148	200	201	931.390	0.0
41	6	12	97229	96700	148	26	44	42	42	212.158	0.0
42	7	12	6474	6597	11	0	0	3	3	21.129	5.11205104692e-07
43	7	12	14612	14218	19	3	2	7	7	33.223	5.11205104692e-07
44	7	12	5785	5790	8	2	1	4	4	13.914	5.11205104692e-07
45	7	12	5551	5549	7	1	1	4	4	12.507	5.11205104692e-07
46	7	12	4597	4597	12	0	0	3	3	22.338	5.11205104692e-07
47	7	12	19114	14817	14	1	1	7	7	25.257	5.11205104692e-07
48	7	12	14847	14604	14	2	1	5	5	26.056	5.11205104692e-07
49	7	12	87301	87414	96	16	27	31	31	170.790	0.0
50	8	15	1876560	1879506	1737	357	506	617	618	2862.724	0.0
51	9	7	2424368	2339996	2547	498	570	815	827	3930.327	0.0
52	10	2	3690347	3625569	5017	608	728	1250	1274	6519.327	0.0
53	11	19	89268	88478	109	16	33	38	38	182.224	0.0
54	12	10	7951	7980	12	2	1	4	4	21.277	5.11205104692e-07
55	12	10	31236	31234	30	3	4	10	10	51.020	5.11205104692e-07
56	12	10	12762	12620	11	0	0	6	6	19.516	5.11205104692e-07
57	12	10	17072	17039	18	2	4	7	7	24.827	5.11205104692e-07
58	12	10	13654	13554	19	1	7	9	9	31.983	5.11205104692e-07
59	12	17	598449	585393	532	104	146	192	191	941.678	0.0
60	13	16	23581	24128	37	3	5	8	7	65.771	5.11205104692e-07

Continued on next page

Table ST-4 – continued from previous page

#	Scaffold		Cluster size (bp)		# Hits	# Unaccounted exons		# Genes found		score	p-value
	n402	CBS513.88	n402	CBS513.88		n402	CBS513.88	n402	CBS513.88		
61	13	16	5267	5395	9	0	0	3	3	17.771	5.11205104692e-07
62	13	16	88784	88368	108	23	29	37	36	185.023	0.0
63	13	16	5674	5674	12	0	0	3	3	23.235	5.11205104692e-07
64	13	16	10795	10806	14	2	2	7	7	26.324	5.11205104692e-07
65	13	16	11680	11678	10	1	3	8	8	19.646	5.11205104692e-07
66	13	16	6172	6178	10	1	2	4	4	17.141	5.11205104692e-07
67	13	16	5877	5877	11	0	1	3	3	21.994	5.11205104692e-07
68	13	16	8368	8384	14	1	1	5	5	26.538	5.11205104692e-07
69	13	16	12068	12074	11	1	1	4	4	18.369	5.11205104692e-07
70	13	16	111629	107480	119	25	34	40	40	198.485	0.0
71	13	16	4357	4357	5	0	0	3	3	10.000	5.11205104692e-07
72	13	16	16113	16515	20	0	8	6	6	36.457	5.11205104692e-07
73	13	16	10441	11093	10	1	2	5	5	17.315	5.11205104692e-07
74	13	16	54102	53377	48	12	3	20	20	88.599	5.11205104692e-07
75	13	16	3084	3096	11	0	0	3	3	21.718	5.11205104692e-07
76	13	16	61448	61653	71	9	11	26	26	120.767	5.11205104692e-07
77	13	16	54706	54000	57	14	14	19	19	100.961	0.0
78	13	16	10924	10898	16	3	4	7	7	29.324	5.11205104692e-07
79	13	16	6230	6288	12	0	0	4	4	23.798	5.11205104692e-07
80	13	16	8352	8352	13	3	1	5	5	24.852	5.11205104692e-07
81	13	16	85337	85223	65	14	16	27	27	115.442	0.0
82	13	16	22217	22232	21	3	6	9	9	33.464	5.11205104692e-07
83	13	16	23651	23533	39	4	4	14	14	65.525	5.11205104692e-07
84	13	16	8896	9242	14	0	2	5	5	23.873	5.11205104692e-07
85	13	16	12956	13057	12	0	2	4	4	20.487	5.11205104692e-07
86	13	16	33984	34173	33	3	12	16	16	55.574	5.11205104692e-07
87	13	16	3586	3586	7	0	0	1	1	14.000	5.11205104692e-07
88	13	16	223416	222992	251	49	68	95	95	458.320	0.0
89	13	16	24335	24316	20	1	5	9	9	33.202	5.11205104692e-07
90	13	16	18050	18043	21	4	6	9	9	32.484	5.11205104692e-07
91	13	16	31230	30762	29	3	5	15	15	51.553	5.11205104692e-07
92	13	16	16851	16850	13	5	0	6	6	22.008	5.11205104692e-07
93	14	16	186744	188645	178	36	46	65	65	310.639	0.0
94	14	16	273789	264686	261	66	56	95	95	444.034	0.0
95	14	16	19945	19418	24	3	7	9	9	36.423	5.11205104692e-07
96	14	16	28683	27943	35	5	12	14	13	57.082	5.11205104692e-07
97	15	3	1582232	1609795	1598	330	456	523	522	2494.486	0.0
98	16	4	1902587	1889319	1671	357	478	619	616	2681.479	0.0
99	17	4	365539	395617	345	56	116	118	120	557.363	0.0
100	18	4	33153	33079	32	3	7	12	12	54.967	5.11205104692e-07
101	18	4	16542	16290	14	0	2	5	5	25.032	5.11205104692e-07
102	18	4	28950	29038	31	6	7	13	13	52.424	5.11205104692e-07
103	18	4	23005	23026	24	4	7	12	12	39.217	5.11205104692e-07
104	19	11	2747323	2719178	4322	463	595	875	901	4999.173	0.0
105	20	18	1582786	1540114	1681	344	467	545	530	2509.615	0.0
106	21	6	192148	192246	174	23	56	68	66	271.886	0.0
107	21	6	53494	55387	51	6	8	18	18	92.804	5.11205104692e-07
108	21	6	8828	8615	8	0	2	3	3	13.927	5.11205104692e-07
109	21	6	23362	23357	10	3	1	6	6	15.599	5.11205104692e-07
110	21	6	30110	29987	30	6	8	13	13	57.424	5.11205104692e-07
111	21	6	38372	38319	44	9	7	17	17	77.317	5.11205104692e-07
112	21	6	17537	17428	18	3	5	9	9	26.740	5.11205104692e-07
113	21	6	4182	4184	6	0	0	3	3	11.997	5.11205104692e-07
114	21	6	15799	15962	20	2	6	7	7	29.799	5.11205104692e-07
115	21	6	5657	5657	9	0	0	3	3	17.794	5.11205104692e-07

Continued on next page

Table ST-4 – continued from previous page

#	Scaffold n402	Scaffold CBS513.88	Cluster size (bp) n402	Cluster size (bp) CBS513.88	# Hits	# Unaccounted exons n402	# Unaccounted exons CBS513.88	# Genes found n402	# Genes found CBS513.88	score	p-value
116	21	6	9967	9927	10	3	0	3	3	17.986	5.11205104692e-07
117	21	8	3305576	2918630	3818	956	776	991	941	4314.013	0.0
118	22	4	21636	21225	24	2	4	8	9	39.342	5.11205104692e-07
119	24	4	12942	12943	17	1	5	7	7	32.321	5.11205104692e-07

Table ST-5: *Clusters discovered by Proteny for the basidiomycota*

#	Scaffold *S. comm.*	Scaffold *A. bisp.*	Cluster size (bp) *S. comm.*	Cluster size (bp) *A. bisp.*	# Hits	# Unaccounted exons *S. comm.*	# Unaccounted exons *A. bisp.*	# Genes found *S. comm.*	# Genes found *A. bisp.*	score	p-value
1	1	1	6222319	3338379	10420	5953	1790	1787	1093	4320.545	0.0
2	1	2	2117	33953	46	0	36	1	6	47.840	3.7790581231e-07
3	1	3	2370	11980	16	0	8	1	3	19.660	3.7790581231e-07
4	1	3	1928	17733	35	2	21	1	5	34.659	3.7790581231e-07
5	1	3	1901	17733	31	3	21	1	5	33.300	3.7790581231e-07
6	1	3	1934	17887	34	4	18	1	5	34.482	3.7790581231e-07
7	1	3	7900	13821	8	1	6	2	4	11.599	3.7790581231e-07
8	1	4	9512	8423	15	1	0	3	3	25.434	3.7790581231e-07
9	1	4	20078	19627	32	14	11	6	6	34.268	1.20126131264e-13
10	1	4	11398	22514	26	1	25	5	5	26.357	3.7790581231e-07
11	1	4	150704	81172	184	102	41	36	28	217.613	0.0
12	1	4	29757	11495	37	27	0	6	5	43.452	3.7790581231e-07
13	1	4	7146	9076	15	1	2	4	4	22.918	3.7790581231e-07
14	1	4	12649	11340	15	4	3	4	4	24.560	3.7790581231e-07
15	1	4	33887	15060	35	22	5	6	5	43.446	3.7790581231e-07
16	1	4	19618	10899	35	15	2	4	4	43.241	3.7790581231e-07
17	1	4	39465	28409	59	26	18	8	8	61.758	0.0
18	1	4	376649	229410	544	338	67	83	81	589.204	0.0
19	1	4	8243	31935	47	7	28	3	8	40.862	3.7790581231e-07
20	1	4	11444	17106	26	1	13	6	5	31.493	3.7790581231e-07
21	1	4	10676	7000	28	8	0	3	2	37.867	3.7790581231e-07
22	1	4	10182	8984	21	12	4	3	3	21.561	3.7790581231e-07
23	1	4	16598	16265	27	16	10	6	6	38.166	3.7790581231e-07
24	1	4	32629	19331	63	24	7	10	9	81.018	3.7790581231e-07
25	1	4	26468	31649	51	13	28	10	10	66.877	0.0
26	1	4	32318	16567	49	44	5	8	7	45.722	3.7790581231e-07
27	1	4	40391	29032	43	19	18	8	8	46.287	0.0
28	1	4	60739	38747	69	42	16	16	13	71.414	0.0
29	1	4	64342	34292	73	31	9	16	15	100.718	3.7790581231e-07
30	1	4	5469	3898	18	4	0	3	3	26.241	3.7790581231e-07
31	1	4	402519	273650	457	303	141	91	87	503.117	0.0
32	1	4	92077	84613	185	57	78	27	28	183.890	0.0
33	1	4	14722	8827	54	19	0	4	4	52.870	3.7790581231e-07
34	1	4	19417	18936	39	7	11	6	6	52.723	3.7790581231e-07
35	1	4	5132	3627	8	3	1	3	2	13.809	3.7790581231e-07
36	1	4	8347	8158	33	0	0	5	5	53.989	3.7790581231e-07
37	1	4	16443	9123	26	12	4	5	5	29.713	3.7790581231e-07
38	1	4	143844	92884	257	102	20	41	39	317.120	0.0
39	1	5	20733	38357	77	24	45	4	8	47.362	6.21114271127e-12
40	1	5	5683	38357	46	1	44	4	8	31.042	3.7790581231e-07
41	1	7	14056	51298	126	13	70	2	6	116.358	0.0
42	1	9	14032	7514	27	10	0	3	2	35.710	3.7790581231e-07
43	1	12	1961	31984	54	2	25	1	8	51.498	3.7790581231e-07
44	1	12	1919	31785	44	2	32	1	8	36.800	3.7790581231e-07

Continued on next page

Table ST-5 – continued from previous page

	Scaffold		Cluster size (bp)			# Unaccounted exons		# Genes found			
**#**	***S. comm.***	***A. bisp.***	***S. comm.***	***A. bisp.***	**# Hits**	***S. comm.***	***A. bisp.***	***S. comm.***	***A. bisp.***	**score**	**p-value**
45	1	12	1925	32017	50	1	23	1	8	58.863	3.7790581231e-07
46	1	14	30067	26158	44	13	21	10	10	47.728	0.0
47	1	14	90154	61800	86	56	12	18	17	104.152	0.0
48	1	14	122733	102750	194	65	52	28	29	248.352	0.0
49	1	14	109031	113538	149	84	51	28	27	159.346	0.0
50	1	14	13269	11235	34	7	10	5	5	39.771	3.7790581231e-07
51	1	14	24907	31508	38	20	12	8	8	48.624	0.0
52	1	14	18316	6455	14	8	1	3	3	18.143	3.7790581231e-07
53	1	14	8298	16712	25	0	16	4	4	23.682	3.7790581231e-07
54	1	14	69951	41013	101	52	17	13	14	113.885	0.0
55	1	14	21088	20797	41	4	7	4	4	61.196	3.7790581231e-07
56	1	15	1961	32669	49	1	30	1	5	40.327	3.7790581231e-07
57	1	15	1922	32672	38	1	36	1	5	25.371	3.7790581231e-07
58	1	17	9398	24076	91	7	14	2	4	115.946	3.7790581231e-07
59	1	18	11410	16563	36	16	13	2	5	28.804	9.00947083604e-09
60	1	18	1121	16557	18	0	13	1	5	19.010	3.7790581231e-07
61	2	2	4432838	3106887	9152	2784	1148	1291	961	7737.229	0.0
62	2	3	6685	4582	18	0	0	3	1	31.516	3.7790581231e-07
63	2	3	22011	17282	72	32	23	3	5	59.111	1.22124532709e-15
64	2	5	5171	6866	11	1	2	2	3	17.750	3.7790581231e-07
65	2	5	16060	2151	35	14	1	4	1	50.315	3.7790581231e-07
66	2	7	15985	18379	86	23	42	4	5	58.445	1.84086079713e-12
67	2	10	6679	4866	36	0	2	3	2	59.112	3.7790581231e-07
68	2	12	31882	35240	108	33	32	7	11	95.420	0.0
69	2	12	1513	21872	27	0	22	1	5	29.930	3.7790581231e-07
70	2	13	6880	1476	17	6	0	3	1	22.019	3.7790581231e-07
71	2	15	22442	32202	84	29	40	3	5	45.267	1.7966662158e-08
72	2	16	15291	1683	48	8	0	4	1	56.298	3.7790581231e-07
73	2	16	61060	15052	109	87	13	8	4	73.670	0.0
74	2	17	2241	23887	32	3	25	1	4	32.715	3.7790581231e-07
75	2	18	6679	1808	18	0	0	3	1	31.646	3.7790581231e-07
76	3	1	548	9474	15	0	3	1	3	23.469	3.7790581231e-07
77	3	6	3456993	2321670	4864	2303	1137	787	598	3456.263	0.0
78	3	7	2423	18777	50	2	21	1	5	58.532	3.7790581231e-07
79	4	1	12192	6770	24	6	4	5	2	32.017	3.7790581231e-07
80	4	2	12720	21709	40	20	13	3	4	32.407	5.89594040257e-10
81	4	3	1402	17736	35	1	21	1	5	30.352	3.7790581231e-07
82	4	3	1865	17736	38	1	17	1	5	42.580	3.7790581231e-07
83	4	4	1879	27886	57	0	20	1	8	52.280	3.7790581231e-07
84	4	6	1249	4968	14	0	1	1	3	21.564	3.7790581231e-07
85	4	7	3279967	2069434	4204	2351	1255	726	558	2555.619	0.0
86	4	8	22926	19389	54	21	17	4	2	58.039	0.0
87	4	8	22935	1858	28	21	0	4	1	34.103	3.7790581231e-07
88	4	10	2640	10182	39	0	0	1	3	48.581	3.7790581231e-07
89	4	12	20269	32047	54	25	24	2	9	41.403	4.48530101949e-13
90	4	12	11879	32168	58	23	21	2	9	55.611	0.0
91	4	15	2284	32645	40	1	36	1	5	32.229	3.7790581231e-07
92	4	18	33922	16563	83	24	7	5	5	71.539	3.7790581231e-07
93	4	19	17507	9396	25	13	4	5	4	28.071	3.7790581231e-07
94	5	1	4974	2238	28	0	0	3	1	36.880	3.7790581231e-07
95	5	3	13285	23912	44	5	12	3	7	63.458	3.7790581231e-07
96	5	3	4257	4355	18	0	0	3	3	31.484	3.7790581231e-07
97	5	3	3817	6872	19	0	3	2	3	31.789	3.7790581231e-07
98	5	3	9906	7670	26	3	1	4	4	36.221	3.7790581231e-07
99	5	3	14223	24488	44	8	25	5	6	48.382	3.7790581231e-07

Continued on next page

Table ST-5 – continued from previous page

	Scaffold		Cluster size (bp)			# Unaccounted exons		# Genes found			
**#**	***S. comm.***	***A. bisp.***	***S. comm.***	***A. bisp.***	**# Hits**	***S. comm.***	***A. bisp.***	***S. comm.***	***A. bisp.***	**score**	**p-value**
100	5	3	11556	8205	22	12	0	5	4	26.552	3.7790581231e-07
101	5	3	1582	17730	30	2	21	1	5	29.546	3.7790581231e-07
102	5	3	21485	13223	16	11	5	3	3	20.989	3.7790581231e-07
103	5	3	25862	19259	53	12	2	6	5	72.732	3.7790581231e-07
104	5	3	7683	9022	12	0	4	2	3	17.495	3.7790581231e-07
105	5	3	26663	12083	49	10	0	7	7	67.561	3.7790581231e-07
106	5	3	7500	17733	39	17	16	2	5	35.600	9.69224700498e-13
107	5	3	9486	12979	35	0	4	5	5	47.572	3.7790581231e-07
108	5	3	8365	12836	27	2	7	4	4	38.153	3.7790581231e-07
109	5	3	13475	57953	53	4	47	4	5	47.869	3.7790581231e-07
110	5	3	65992	58329	63	35	24	8	10	54.893	0.0
111	5	3	4777	4032	17	0	1	3	2	25.579	3.7790581231e-07
112	5	3	13320	11814	15	12	2	4	4	19.491	3.7790581231e-07
113	5	3	12030	12308	19	5	2	3	4	30.636	3.7790581231e-07
114	5	3	7368	13495	29	1	8	4	5	35.348	3.7790581231e-07
115	5	4	6423	4576	30	0	1	3	2	44.033	3.7790581231e-07
116	5	7	2505	18720	57	2	14	1	5	70.552	3.7790581231e-07
117	5	7	2426	18747	44	3	27	1	5	60.313	3.7790581231e-07
118	5	7	2474	18723	45	2	26	1	5	62.765	3.7790581231e-07
119	5	12	7370	35065	50	16	30	2	9	29.548	4.18003388569e-08
120	5	12	1009	9962	16	0	13	1	4	19.836	3.7790581231e-07
121	5	12	1725	31644	44	2	28	1	8	41.803	3.7790581231e-07
122	5	17	2539	24507	49	0	15	1	4	52.166	3.7790581231e-07
123	6	2	6172	21563	18	2	13	3	2	19.336	3.7790581231e-07
124	6	2	9143	47333	54	10	41	2	7	59.684	3.7790581231e-07
125	6	2	10794	33959	68	20	36	2	6	49.564	3.42059713887e-13
126	6	2	865	10270	10	0	1	1	5	19.371	3.7790581231e-07
127	6	3	4641	17733	48	7	21	2	5	46.211	3.7790581231e-07
128	6	10	10945	3023	11	11	0	3	3	18.915	3.7790581231e-07
129	6	10	8006	7614	26	2	1	3	3	38.955	3.7790581231e-07
130	6	10	34995	43255	50	20	25	5	6	51.968	0.0
131	6	10	13482	9515	26	14	1	4	4	29.609	3.7790581231e-07
132	6	10	17179	13949	37	9	2	4	4	54.152	3.7790581231e-07
133	6	10	15564	7536	20	13	3	3	3	20.984	3.7790581231e-07
134	6	10	24813	9108	44	34	0	3	4	33.872	3.7790581231e-07
135	6	10	27562	20748	30	22	4	7	6	31.159	3.7790581231e-07
136	6	10	12515	7498	24	6	0	3	2	35.401	3.7790581231e-07
137	6	10	8655	6921	28	9	0	2	4	40.850	3.7790581231e-07
138	6	12	24282	31966	89	30	30	3	8	65.020	1.09956488359e-12
139	6	13	22915	21927	38	12	7	5	4	50.647	3.7790581231e-07
140	6	13	20265	11406	31	11	0	5	4	36.637	3.7790581231e-07
141	6	13	9890	42636	94	0	53	3	3	84.465	3.7790581231e-07
142	6	15	9803	8613	16	4	2	2	3	22.476	3.7790581231e-07
143	6	15	10664	5787	25	5	0	3	2	34.504	3.7790581231e-07
144	6	15	39119	5356	50	35	0	5	2	52.542	3.7790581231e-07
145	6	15	6258	6049	18	1	0	2	2	25.585	3.7790581231e-07
146	7	2	1950	33962	43	0	37	1	6	49.904	3.7790581231e-07
147	7	3	1972	17985	36	2	20	1	5	43.437	3.7790581231e-07
148	7	3	1824	17736	36	0	20	1	5	38.696	3.7790581231e-07
149	7	3	24403	27292	59	18	40	4	3	34.554	3.31538574372e-10
150	7	5	5850	21808	40	1	32	3	4	42.182	3.7790581231e-07
151	7	5	221235	133839	265	195	41	42	42	258.029	0.0
152	7	5	43085	8333	35	32	0	6	4	29.628	3.7790581231e-07
153	7	5	47735	32688	49	31	17	10	10	45.621	0.0
154	7	5	8225	13807	34	0	3	4	5	51.363	3.7790581231e-07

Continued on next page

Table ST-5 – continued from previous page

	Scaffold		Cluster size (bp)			# Unaccounted exons		# Genes found			
**#**	***S. comm.***	***A. bisp.***	***S. comm.***	***A. bisp.***	**# Hits**	***S. comm.***	***A. bisp.***	***S. comm.***	***A. bisp.***	**score**	**p-value**
155	7	5	15110	9718	20	11	8	3	3	19.060	3.7790581231e-07
156	7	5	69967	46989	156	81	63	13	10	73.676	2.68451927354e-13
157	7	5	31802	43406	94	36	47	7	10	70.324	0.0
158	7	5	19778	16261	45	18	2	5	5	62.976	3.7790581231e-07
159	7	5	4826	7020	11	0	1	2	3	19.160	3.7790581231e-07
160	7	5	7122	6783	17	1	0	4	2	28.022	3.7790581231e-07
161	7	5	9096	22571	29	1	19	4	4	39.493	3.7790581231e-07
162	7	5	22780	13393	33	17	10	4	4	41.322	3.7790581231e-07
163	7	5	35456	32700	44	15	15	10	10	64.862	0.0
164	7	5	25012	24874	47	14	27	7	6	58.428	0.0
165	7	5	21469	19943	43	10	16	6	5	49.744	3.7790581231e-07
166	7	5	34275	24373	49	26	7	9	9	64.268	3.7790581231e-07
167	7	5	15610	18879	35	4	9	5	5	47.500	3.7790581231e-07
168	7	5	31774	16626	51	30	6	7	6	53.806	3.7790581231e-07
169	7	5	22957	20254	51	11	7	9	7	75.476	3.7790581231e-07
170	7	5	10556	15973	19	3	13	3	3	25.488	3.7790581231e-07
171	7	5	26572	32863	49	18	24	9	9	46.545	0.0
172	7	5	7116	6946	22	2	1	4	3	35.215	3.7790581231e-07
173	7	5	24182	9478	33	28	1	4	4	29.766	3.7790581231e-07
174	7	5	38110	24395	61	35	9	8	8	73.862	3.7790581231e-07
175	7	5	15560	15675	31	6	1	6	6	49.053	3.7790581231e-07
176	7	7	2432	18723	43	2	28	1	5	53.483	3.7790581231e-07
177	7	11	4139	4988	20	1	3	2	3	28.501	3.7790581231e-07
178	7	12	1811	32213	54	0	18	1	8	51.912	3.7790581231e-07
179	7	12	17074	31972	54	22	27	2	8	38.015	2.68849387197e-11
180	7	12	13166	32180	59	15	21	2	9	66.604	0.0
181	7	15	2115	32919	43	2	33	1	5	37.454	3.7790581231e-07
182	7	16	29228	30636	44	5	11	6	6	57.370	3.7790581231e-07
183	8	3	18407	2175	39	3	0	4	1	64.188	3.7790581231e-07
184	8	3	4631	17982	57	7	20	2	5	69.966	3.7790581231e-07
185	8	3	8725	36057	103	13	67	3	7	115.708	0.0
186	8	5	13124	10819	33	10	4	3	3	42.735	3.7790581231e-07
187	8	5	22388	10807	27	15	4	4	4	35.329	3.7790581231e-07
188	8	5	22551	21149	49	10	6	8	8	81.058	3.7790581231e-07
189	8	5	17831	19355	30	6	16	7	6	32.590	3.7790581231e-07
190	8	5	5182	5027	13	2	0	3	3	23.205	3.7790581231e-07
191	8	5	5565	8997	26	0	9	3	3	29.200	3.7790581231e-07
192	8	5	60885	36453	88	43	12	10	10	87.418	0.0
193	8	5	104210	57665	129	102	27	17	18	142.627	0.0
194	8	5	9592	4756	11	9	1	3	3	17.500	3.7790581231e-07
195	8	5	9213	7679	23	3	1	3	3	30.957	3.7790581231e-07
196	8	5	14499	7760	14	4	1	3	3	22.738	3.7790581231e-07
197	8	5	19320	20351	21	9	6	6	6	21.780	3.7790581231e-07
198	8	5	16018	13166	28	6	2	4	5	40.009	3.7790581231e-07
199	8	5	10718	11611	25	3	7	5	5	29.681	3.7790581231e-07
200	8	5	37077	13834	44	36	1	7	7	48.645	3.7790581231e-07
201	8	5	6917	15538	21	0	5	5	5	30.659	3.7790581231e-07
202	8	5	117444	66564	157	86	25	17	15	144.293	0.0
203	8	5	89424	50537	130	95	18	19	18	124.609	0.0
204	8	5	5829	4789	12	0	0	3	3	20.062	3.7790581231e-07
205	8	5	10897	11215	22	15	4	3	4	28.883	3.7790581231e-07
206	8	5	4273	3556	10	2	1	2	3	15.558	3.7790581231e-07
207	8	5	19234	9259	31	7	12	3	2	31.246	3.7790581231e-07
208	8	5	1819	2574	11	0	1	2	3	19.811	3.7790581231e-07
209	8	6	8740	2328	29	6	0	3	1	38.610	3.7790581231e-07

Continued on next page

Table ST-5 – continued from previous page

	Scaffold		Cluster size (bp)			# Unaccounted exons		# Genes found			
**#**	***S. comm.***	***A. bisp.***	***S. comm.***	***A. bisp.***	**# Hits**	***S. comm.***	***A. bisp.***	***S. comm.***	***A. bisp.***	**score**	**p-value**
210	8	7	4689	11566	20	3	15	2	3	18.586	3.7790581231e-07
211	8	7	2428	18726	44	4	27	1	5	54.882	3.7790581231e-07
212	8	9	8740	15856	49	7	15	3	2	57.478	3.7790581231e-07
213	8	10	8725	4489	38	15	3	3	2	39.839	3.7790581231e-07
214	8	10	8740	2101	24	11	1	3	1	26.685	3.7790581231e-07
215	8	12	8737	7187	50	8	4	3	2	75.933	3.7790581231e-07
216	8	12	28627	35240	146	48	26	4	9	162.062	0.0
217	8	12	8737	20933	44	10	22	3	2	43.848	3.7790581231e-07
218	8	12	5039	32168	94	4	21	2	8	106.743	3.7790581231e-07
219	8	12	1291	10031	16	2	13	1	4	19.329	3.7790581231e-07
220	8	15	28978	5619	49	24	0	7	2	68.664	3.7790581231e-07
221	8	15	8731	32931	104	11	38	3	5	110.060	0.0
222	8	15	13302	43594	74	11	49	3	7	62.615	0.0
223	8	16	8737	29094	81	12	58	3	4	61.562	0.0
224	8	16	26882	15692	39	30	3	3	3	35.417	3.7790581231e-07
225	8	16	7269	12409	25	1	11	3	3	28.526	3.7790581231e-07
226	8	17	2328	24082	42	1	16	1	4	53.220	3.7790581231e-07
227	9	2	818	10267	9	0	2	1	5	16.678	3.7790581231e-07
228	9	3	1458	17376	31	1	21	1	5	28.408	3.7790581231e-07
229	9	3	1828	17531	28	2	23	1	5	32.278	3.7790581231e-07
230	9	3	15003	36054	90	18	63	3	7	80.350	0.0
231	9	3	1981	17985	37	3	18	1	5	32.056	3.7790581231e-07
232	9	6	15262	2328	22	13	1	3	1	26.660	3.7790581231e-07
233	9	8	63446	16802	43	19	6	7	3	55.595	3.7790581231e-07
234	9	8	33156	34010	48	27	16	7	6	34.391	4.61944926755e-10
235	9	8	14262	25920	34	9	25	4	4	38.249	3.7790581231e-07
236	9	8	37661	34567	119	40	26	6	7	75.003	2.70013867931e-08
237	9	8	7740	15904	18	0	7	3	3	24.461	3.7790581231e-07
238	9	8	8144	5390	21	3	0	2	3	27.943	3.7790581231e-07
239	9	9	24320	7187	40	31	5	3	2	30.656	3.7790581231e-07
240	9	9	11743	29810	21	9	11	2	3	27.336	3.7790581231e-07
241	9	9	10248	13597	23	1	8	3	3	33.984	3.7790581231e-07
242	9	9	12759	15701	28	12	6	4	5	43.188	3.7790581231e-07
243	9	9	9408	16816	31	0	7	4	3	37.866	3.7790581231e-07
244	9	9	12111	4980	19	13	0	3	2	27.283	3.7790581231e-07
245	9	9	16413	12404	37	16	5	5	5	37.339	3.7790581231e-07
246	9	9	15005	1780	20	15	1	3	1	20.234	3.7790581231e-07
247	9	9	15006	2085	21	14	0	3	1	22.585	3.7790581231e-07
248	9	9	28188	28917	77	23	37	8	8	75.462	0.0
249	9	9	15965	28898	41	12	21	4	3	40.667	2.77555756156e-15
250	9	9	11291	7252	16	10	5	2	3	16.915	3.7790581231e-07
251	9	10	14991	4295	33	16	4	3	2	32.184	3.7790581231e-07
252	9	12	15253	7187	46	11	4	3	2	57.919	3.7790581231e-07
253	9	12	123813	57794	210	154	51	8	11	149.025	0.0
254	9	12	2234	31972	50	4	29	1	8	39.174	3.7790581231e-07
255	9	12	6505	45805	56	7	43	3	10	44.523	3.7790581231e-07
256	9	13	9774	43751	34	12	20	3	8	38.372	0.0
257	9	15	36896	37251	100	49	41	5	6	73.676	0.0
258	9	15	2114	32645	39	2	36	1	5	23.742	3.7790581231e-07
259	9	16	14976	29085	75	14	61	3	4	40.752	1.0953460361e-11
260	9	17	16440	3908	24	17	2	5	2	22.175	3.7790581231e-07
261	9	19	15253	1939	19	16	1	3	1	20.033	3.7790581231e-07
262	10	3	54094	55085	171	76	94	6	9	131.622	0.0
263	10	9	9022	15844	47	12	18	4	2	40.409	2.68436384232e-10
264	10	10	9037	1865	33	7	0	4	1	39.527	3.7790581231e-07

Continued on next page

Table ST-5 – continued from previous page

	Scaffold		Cluster size (bp)			# Unaccounted exons		# Genes found			
#	*S. comm.*	*A. bisp.*	*S. comm.*	*A. bisp.*	# Hits	*S. comm.*	*A. bisp.*	*S. comm.*	*A. bisp.*	score	p-value
265	10	11	35419	7085	24	19	0	3	3	25.210	3.7790581231e-07
266	10	11	41564	41557	68	33	20	6	6	71.756	0.0
267	10	11	23310	37147	44	9	15	7	7	49.594	3.7790581231e-07
268	10	11	13264	12915	24	2	4	2	3	32.626	3.7790581231e-07
269	10	11	3691	7074	12	2	4	2	3	17.403	3.7790581231e-07
270	10	11	5204	9795	39	0	0	2	3	59.802	3.7790581231e-07
271	10	11	12865	9110	19	10	4	2	3	22.819	3.7790581231e-07
272	10	11	8242	11608	18	2	1	3	3	28.775	3.7790581231e-07
273	10	11	12106	9001	13	3	0	4	4	23.461	3.7790581231e-07
274	10	11	13764	7103	18	9	7	3	3	20.016	3.7790581231e-07
275	10	11	17845	44596	49	4	45	4	3	29.856	3.7790581231e-07
276	10	11	59001	44921	87	33	26	11	10	100.474	0.0
277	10	12	24777	24432	58	27	27	5	3	34.038	6.68513744362e-09
278	10	12	9040	20933	53	8	21	4	3	41.766	3.7790581231e-07
279	10	12	82243	54130	173	95	62	9	11	82.234	5.01820807131e-14
280	10	12	7860	32180	39	6	29	2	9	35.956	3.7790581231e-07
281	10	15	42901	32663	143	57	35	5	5	115.694	0.0
282	10	16	9019	29061	91	14	63	4	4	65.899	0.0
283	11	2	7096	10216	19	7	1	2	5	25.635	3.7790581231e-07
284	11	2	20172	6287	25	18	4	3	2	25.821	3.7790581231e-07
285	11	3	9539	17982	39	8	18	2	5	36.381	3.7790581231e-07
286	11	4	18876	4148	25	23	0	5	4	23.577	3.7790581231e-07
287	11	8	9812	8227	20	3	0	4	4	28.703	3.7790581231e-07
288	11	8	30561	19197	36	26	1	6	5	49.050	3.7790581231e-07
289	11	8	5733	5979	15	1	1	3	3	24.747	3.7790581231e-07
290	11	8	67745	35645	49	37	8	8	8	61.895	3.7790581231e-07
291	11	8	17431	16509	28	15	1	5	5	37.799	3.7790581231e-07
292	11	8	17509	6236	25	15	7	4	2	22.477	3.7790581231e-07
293	11	12	9746	32207	48	8	20	2	8	52.696	3.7790581231e-07
294	11	15	9749	19745	38	8	23	2	4	33.013	3.7790581231e-07
295	12	2	4376	30494	44	4	8	2	5	53.261	3.7790581231e-07
296	12	3	35666	31858	59	25	22	10	11	63.713	0.0
297	12	3	9293	15939	34	5	24	2	3	28.941	3.7790581231e-07
298	12	3	15005	10905	24	3	6	3	2	32.661	3.7790581231e-07
299	12	3	35733	20712	107	29	31	14	6	104.150	0.0
300	12	3	80238	68004	192	54	43	15	14	213.461	0.0
301	13	1	16572	16853	32	4	13	6	7	51.016	3.7790581231e-07
302	13	1	79802	52160	125	46	13	20	18	160.103	0.0
303	13	1	9535	7992	21	2	3	3	3	30.600	3.7790581231e-07
304	13	1	221375	239035	417	158	226	66	64	384.664	0.0
305	13	1	9160	6574	25	2	0	3	2	35.334	3.7790581231e-07
306	13	1	10722	9124	32	1	2	3	2	43.547	3.7790581231e-07
307	13	1	23242	4281	22	13	0	4	2	24.058	3.7790581231e-07
308	13	1	27528	13887	23	21	0	4	4	33.506	3.7790581231e-07
309	13	1	42067	8603	55	14	2	7	3	72.229	3.7790581231e-07
310	13	1	4873	3921	15	1	0	3	4	24.961	3.7790581231e-07
311	13	1	33031	11293	33	27	2	5	4	41.249	3.7790581231e-07
312	13	1	27863	34948	66	9	54	7	7	55.266	3.7790581231e-07
313	13	1	34872	29466	94	14	0	9	9	135.941	3.7790581231e-07
314	13	1	92218	74285	153	41	22	20	22	197.376	0.0
315	14	9	15018	11097	47	22	10	3	4	46.151	3.7790581231e-07
316	14	9	13423	11085	29	14	10	2	4	28.344	3.7790581231e-07
317	14	12	31159	30624	42	17	17	6	4	39.877	4.06341627013e-14
318	14	17	13939	6580	16	4	1	3	3	23.421	3.7790581231e-07
319	15	10	67009	2602	25	14	1	8	2	34.173	3.7790581231e-07

Continued on next page

Table ST-5 – continued from previous page

	Scaffold		Cluster size (bp)			# Unaccounted exons		# Genes found			
**#**	***S. comm.***	***A. bisp.***	***S. comm.***	***A. bisp.***	**# Hits**	***S. comm.***	***A. bisp.***	***S. comm.***	***A. bisp.***	**score**	**p-value**
320	16	1	653	9474	15	0	3	1	3	24.661	3.7790581231e-07
321	16	1	26949	23776	30	12	10	7	4	31.298	3.7790581231e-07
322	16	1	105530	62101	103	89	14	21	16	98.072	0.0
323	16	1	4665	5803	18	1	2	3	3	30.489	3.7790581231e-07
324	16	12	1269	10001	21	0	8	1	4	28.131	3.7790581231e-07
325	16	17	22719	25047	28	17	4	4	4	31.107	3.7790581231e-07
326	16	17	17290	65708	102	25	44	5	5	81.285	0.0
327	17	1	38869	7128	27	19	3	5	2	33.437	3.7790581231e-07
328	17	1	19417	11370	33	12	1	5	4	45.186	3.7790581231e-07
329	17	3	9418	1541	21	5	1	4	1	27.002	3.7790581231e-07
330	17	6	9367	1719	25	2	1	4	1	34.161	3.7790581231e-07
331	17	12	1678	21450	32	0	19	1	5	29.174	3.7790581231e-07
332	17	12	9469	21881	104	4	19	4	5	151.651	3.7790581231e-07
333	17	15	6536	1559	20	0	0	3	1	33.829	3.7790581231e-07
334	18	4	5466	9619	13	0	3	3	3	22.468	3.7790581231e-07
335	18	4	8010	2049	16	7	0	3	1	24.100	3.7790581231e-07
336	18	4	18842	2034	22	15	0	4	1	31.423	3.7790581231e-07
337	18	4	18842	2019	22	15	0	4	1	32.604	3.7790581231e-07
338	18	4	8013	2019	16	7	0	3	1	25.041	3.7790581231e-07
339	18	13	1896	26505	25	1	23	2	8	23.805	3.7790581231e-07
340	19	6	6253	12424	30	1	1	2	4	46.496	3.7790581231e-07
341	19	12	8736	16012	11	0	6	3	2	17.388	3.7790581231e-07
342	19	12	27643	49872	63	23	13	5	7	60.241	5.21804821574e-15
343	20	3	5050	36211	85	7	58	2	7	57.662	3.7790581231e-07
344	20	4	68492	27605	108	83	9	10	10	100.639	3.7790581231e-07
345	20	12	5056	32207	92	7	19	2	8	86.391	3.7790581231e-07

Table ST-6: *Clusters discovered by i-ADHoRe for the aspergillus genomes*

	Scaffold		Cluster size (bp)			# Unaccounted exons		# Genes found			
**#**	**n402**	**CBS513.88**	**n402**	**CBS513.88**	**# Hits**	**n402**	**CBS513.88**	**n402**	**CBS513.88**	**score**	**p-value**
1	1	5	40021	122055	26	24	22	9	12	-20.4141421598	0.729051902543
2	1	5	40021	45920	26	24	22	4	4	-20.4141421598	0.729051902543
3	1	5	58072	33316	5	45	35	3	3	-56.5918208263	0.297
4	1	7	45679	61636	3	29	35	3	3	-45.7150461704	0.327
5	1	9	2217697	2163025	1904	266	367	55	55	2548.23660711	0.0
6	1	9	1136507	1103206	546	125	168	31	31	912.03184605	0.0
7	1	9	1575693	1585561	2036	276	380	445	448	2735.47356393	0.0
8	1	9	58072	319316	69	5	309	11	14	-132.17200032	0.000264410364886
9	1	9	100490	95989	37	69	55	7	7	-22.2871978916	0.0
10	2	12	86703	213817	11	94	155	22	26	-166.524685349	0.00357321696411
11	2	12	44163	47761	1	0	0	3	3	0.893815635939	0.323
12	2	14	383412	403647	320	102	140	56	57	458.420675827	0.0
13	2	14	1722871	1700162	2036	355	424	519	518	2860.15967846	0.0
14	2	14	154390	35543	27	30	10	10	8	22.0179296622	0.000264410364886
15	2	14	596364	56166	55	407	18	15	12	-225.684809437	1.31006316906e-14
16	2	14	27485	52192	5	4	1	3	3	3.35800293811	0.015
17	3	13	904504	887459	1052	152	223	259	257	1544.09057359	0.0
18	3	13	327355	64923	79	208	20	25	22	-6.27778542545	0.0
19	3	13	46017	34230	5	56	43	3	3	-76.5268809898	0.809
20	3	13	36356	57707	5	34	72	3	3	-81.4337072801	0.822
21	3	13	37643	63145	1	0	0	3	3	0.0	1.0
22	4	1	3752509	3625678	5079	579	666	1058	1065	6374.15106032	0.0

Continued on next page

Table ST-6 – continued from previous page

#	Scaffold n402	Scaffold CBS513.88	Cluster size (bp) n402	Cluster size (bp) CBS513.88	# Hits	# Unaccounted exons n402	# Unaccounted exons CBS513.88	# Genes found n402	# Genes found CBS513.88	score	p-value
23	4	1	2820518	86375	118	2656	14	28	24	-1927.67521918	8.63487141668e-05
24	4	1	2820518	2729411	3516	438	568	56	56	4743.33625399	0.0
25	4	1	98843	2882735	110	24	2824	24	26	-1985.26568124	1.65580765077e-06
26	4	1	3477528	2882735	3656	810	557	54	53	4621.33975418	0.0
27	4	1	237222	28654	23	70	10	9	7	-10.1997519811	0.000264410364886
28	4	1	46536	550878	79	10	500	13	15	-237.089697405	0.000264410364886
29	4	1	7306	7316	7	0	4	3	3	10.774717506	0.000264410364886
30	4	3	152534	23256	2	28	11	3	3	-24.7538497153	0.05
31	4	5	98843	93729	7	85	75	4	4	-105.942899785	0.0065
32	4	9	46536	67774	3	26	63	3	3	-63.1234217036	0.228
33	5	5	158315	104824	47	106	67	16	16	-42.1300835789	0.0
34	5	5	308541	308770	337	55	107	86	88	478.544311362	0.0
35	5	5	163265	163800	91	76	111	27	29	25.8897772761	0.0
36	5	6	8342	282004	3	2	2	4	7	-0.109647626721	0.235
37	5	6	8342	9060	3	2	2	3	3	-0.109647626721	0.235
38	5	12	879060	875876	914	177	235	185	186	1207.73877694	0.0
39	6	3	100003	32264	9	29	22	3	3	-36.901610158	0.861
40	6	3	78853	66971	8	61	10	4	4	-48.890721917	0.396
41	6	4	72298	44617	6	45	53	3	3	-73.1594245896	0.686
42	6	5	276374	285279	222	38	67	69	69	362.40022466	0.0
43	6	11	115240	53383	10	13	48	4	4	-41.9086046428	0.727
44	6	12	607470	596231	632	136	158	184	186	1072.71214394	0.0
45	6	12	608331	610507	635	73	141	154	160	986.907941112	0.0
46	6	12	89800	89271	144	25	40	34	34	205.387016335	0.0
47	6	16	86473	116132	12	113	103	5	5	-176.020833799	0.998808474987
48	7	12	281246	274504	245	66	102	64	63	378.047599679	0.0
49	8	3	141881	112704	10	158	140	5	5	-223.953916687	0.595
50	8	3	37547	32347	10	29	33	3	3	-41.4453865921	0.552
51	8	11	129514	163458	17	130	184	5	5	-251.750251609	0.999189624783
52	8	15	863845	878049	755	138	194	18	17	1171.32894186	0.0
53	8	15	370953	308984	291	88	75	15	15	417.967240207	0.0
54	8	15	1872314	1854343	2149	309	401	558	563	3120.94035874	0.0
55	8	15	1184300	167584	198	998	36	53	47	-498.190790861	0.0
56	8	15	1178744	1188240	1234	208	254	79	76	1921.22000486	0.0
57	8	15	349606	36957	45	51	2	11	8	37.0336616585	0.000264410364886
58	8	15	349606	324208	271	56	72	18	18	444.894897232	0.0
59	8	15	37290	726015	36	2	618	11	14	-431.04848364	0.089
60	8	18	20923	89003	7	4	47	4	4	-35.62621943	0.69
61	8	18	37290	25225	3	25	26	3	3	-38.8449379196	0.7
62	9	7	2808667	2696397	2909	456	508	24	22	4125.20094372	0.0
63	9	7	2424372	2340057	2939	455	508	727	738	4157.29052953	0.0
64	9	7	99504	1698703	116	19	1838	30	35	-1231.0375883	1.3735655946e-08
65	9	7	26501	1182775	56	5	1188	10	14	-854.616070503	0.217
66	9	7	1186305	1179186	1301	228	316	19	19	1944.47789525	0.0
67	9	7	1136507	61636	59	1090	10	18	16	-750.492807397	0.000264410364886
68	9	7	708966	27259	30	601	7	11	10	-415.455842409	0.000528820729773
69	9	7	708966	649364	599	192	195	25	26	919.490963889	0.0
70	9	11	16731	1680449	11	14	1841	6	9	-1413.86425825	0.8396645808
71	9	11	16731	12621	2	2	0	3	3	-1.30316239316	0.838
72	9	14	95605	403647	18	83	392	28	32	-335.711808773	0.0538199388762
73	9	14	95605	131183	12	87	128	4	5	-156.752134897	0.500258429049
74	9	15	22926	30510	5	24	25	3	3	-33.8059287997	0.426
75	9	17	167843	191502	12	189	213	7	8	-310.415988621	0.893775673984
76	10	2	3690350	3625572	5015	606	726	1118	1131	6516.38647148	0.0
77	10	2	107473	2167445	143	16	2274	28	33	-1537.6841322	1.24547935076e-07

Continued on next page

Table ST-6 – continued from previous page

	Scaffold		Cluster size (bp)			# Unaccounted exons		# Genes found			
**#**	**n402**	**CBS513.88**	**n402**	**CBS513.88**	**# Hits**	**n402**	**CBS513.88**	**n402**	**CBS513.88**	**score**	**p-value**
78	10	2	2420928	85010	136	2378	24	33	30	-1642.60029897	4.61963800547e-13
79	10	2	46211	2696397	43	2	2937	12	13	-2185.09396235	0.261
80	10	2	33651	2334420	76	6	2435	11	13	-1753.20070835	0.0105
81	10	3	33651	10672	8	40	13	3	3	-36.7532042612	0.624
82	10	7	46211	31166	6	0	10	3	3	-3.88121275217	0.559
83	10	8	74661	82034	4	71	78	3	3	-109.574091262	0.321
84	10	9	107473	156995	15	102	125	5	7	-169.897293485	0.825113373673
85	10	16	155559	114451	10	127	155	5	5	-184.113999448	0.000264410364886
86	11	19	80152	79401	105	14	29	29	29	178.088361579	0.0
87	12	2	2610745	39112	14	219	43	21	19	-204.652444282	0.949988271788
88	12	2	67594	39112	7	12	47	4	4	-41.3952609567	0.617
89	12	10	242185	247968	181	44	70	58	57	243.157671353	0.0
90	12	11	38726	41870	9	30	43	3	3	-51.1240590413	0.621
91	12	17	1438867	1698703	538	97	131	67	67	871.724317716	0.0
92	12	17	598451	585921	607	104	142	170	172	981.392251167	0.0
93	12	17	142745	156276	137	17	36	42	43	231.098655077	0.0
94	13	3	72626	64175	4	63	50	3	3	-76.7643638976	0.034
95	13	14	43800	56166	4	16	5	4	4	-13.5268962651	0.499
96	13	15	174756	244131	44	194	256	8	10	-328.671887343	0.926740287964
97	13	16	1436152	1127798	1194	257	315	85	86	1797.2743536	0.0
98	13	16	349153	338630	208	53	165	25	27	261.305930066	0.0
99	13	16	1739812	1451711	1804	302	425	431	431	2492.64564184	0.0
100	13	16	1072565	116132	133	612	32	34	31	-239.564082211	0.0
101	14	3	48556	61359	12	49	50	3	3	-69.440042699	0.628525531865
102	14	13	80214	57176	9	97	71	3	3	-126.017013265	0.704
103	14	14	57936	60995	6	1	12	4	4	-1.63156204986	0.029
104	14	16	659239	643535	585	138	173	185	184	925.131256928	0.0
105	15	1	651901	29007	11	302	38	13	12	-252.787775088	0.274940035005
106	15	1	72285	86375	10	78	62	4	5	-109.311825151	0.95
107	15	1	34717	29007	5	50	42	3	3	-65.6813074666	0.308
108	15	3	1582234	1609797	2279	291	381	443	450	2926.17184562	0.0
109	15	3	349153	64175	28	210	13	14	14	-120.04137005	4.02785071962e-08
110	15	3	873971	66971	71	868	21	22	19	-570.89724416	6.12938255706e-10
111	15	3	220428	61359	60	230	7	24	21	-82.4703175703	0.000264410364886
112	15	10	74159	60119	10	62	45	4	4	-78.6683871179	0.74
113	15	11	111178	92902	12	96	63	6	6	-124.990757075	0.978006636153
114	15	11	24076	41870	10	21	45	3	3	-49.5224391831	0.961
115	15	11	40339	55264	11	34	49	3	3	-58.1477432358	0.683440162805
116	15	15	109196	104980	14	105	123	6	6	-186.350385969	0.999645303392
117	15	15	32273	36957	9	35	31	3	3	-52.5812773937	0.991
118	15	15	76964	71372	6	70	74	3	3	-107.074067092	0.52
119	15	18	101513	57354	11	46	69	3	3	-81.207517521	0.540812172967
120	16	1	41638	28654	2	0	3	3	3	-1.98077737425	0.796
121	16	3	108178	129692	10	80	166	6	6	-187.637142984	0.859
122	16	4	1902589	1889324	1866	329	425	577	573	2778.92124337	0.0
123	16	4	108178	272247	86	17	72	32	36	115.501104484	0.0
124	16	4	245728	252505	191	51	70	56	56	301.382746699	0.0
125	16	4	275854	59597	49	11	8	24	22	79.1884302983	0.000264410364886
126	16	12	36855	16469	1	0	0	3	3	0.0	1.0
127	17	4	365565	350147	345	57	74	99	98	588.165393288	0.0
128	17	4	74403	44617	61	5	11	18	15	93.5929726386	0.000264410364886
129	17	13	58014	64923	4	66	87	3	3	-115.97871768	0.611
130	18	4	148863	163819	110	45	88	39	39	151.747986508	0.0
131	18	11	50357	55431	7	41	58	3	3	-70.9443088093	0.45
132	19	2	67233	98200	8	64	94	4	4	-119.745579968	0.787

Continued on next page

Table ST-6 – continued from previous page

#	Scaffold n402	Scaffold CBS513.88	Cluster size (bp) n402	Cluster size (bp) CBS513.88	# Hits	# Unaccounted exons n402	# Unaccounted exons CBS513.88	# Genes found n402	# Genes found CBS513.88	score	p-value
133	19	2	83773	76217	14	89	61	3	3	-104.447156249	0.365195523642
134	19	3	55215	40989	11	46	47	3	3	-66.1470588214	0.689423917628
135	19	3	86013	54099	11	70	75	3	3	-116.206480759	0.992989395014
136	19	6	122659	121076	7	100	102	4	4	-159.474380672	0.951
137	19	7	23716	71910	3	24	68	3	3	-70.4456016242	0.719
138	19	9	193259	207577	23	151	198	6	8	-236.777086736	0.0206579416051
139	19	10	122563	42190	6	49	16	4	4	-46.7193748661	0.544
140	19	10	39676	25143	7	25	15	3	3	-19.0263100901	0.016
141	19	11	2747326	2719184	4306	463	595	730	735	4991.51095872	0.0
142	19	11	122563	1014767	148	16	1023	21	23	-614.493781771	2.03144888133e-07
143	19	11	106279	1383921	175	26	1383	24	28	-835.610331223	2.01116900911e-12
144	19	11	637345	152586	220	418	40	37	33	-15.9630149652	0.0
145	19	11	635229	571850	603	145	144	59	57	860.487291748	0.0
146	19	11	99117	41870	47	31	12	13	11	54.0894134433	0.0
147	19	11	1191020	33365	54	847	13	15	12	-581.160555461	3.16288483337e-06
148	19	11	1191020	1159085	1469	242	308	26	26	1822.72101156	0.0
149	19	11	15280	741987	37	4	625	4	6	-430.91204616	0.028
150	19	11	1079012	41870	63	1064	10	13	10	-759.861281462	0.00396720444327
151	19	11	999190	53383	73	989	14	17	14	-682.690409999	4.53390488347e-05
152	19	11	86013	602413	132	8	531	17	19	-217.856605154	0.000264410364886
153	19	15	20917	308984	7	17	256	5	8	-216.164588402	0.989
154	19	15	20917	28362	3	17	32	3	3	-36.8425233599	0.693
155	19	16	30793	1310325	38	17	1451	6	9	-1085.34595811	0.468134374943
156	19	16	30793	7477	4	25	3	3	3	-17.2601044761	0.205
157	19	17	108812	63525	5	108	66	6	6	-140.051416505	0.972
158	19	18	17176	35243	5	14	30	3	3	-28.7576128375	0.302
159	20	2	87750	85010	17	91	106	4	5	-144.423493849	0.782939638573
160	20	7	51106	27259	4	60	22	3	3	-65.0623551856	0.879
161	20	11	43795	33365	4	34	45	3	3	-50.8707742306	0.021
162	20	12	16620	7847	2	18	3	3	3	-10.7060037932	0.012
163	20	12	14546	27090	3	13	24	3	3	-23.9614289584	0.172
164	20	14	561647	29571	8	48	32	11	9	-49.4863429883	0.037
165	20	14	90929	36050	9	48	40	5	5	-55.0794817922	0.056
166	20	18	2420928	2326117	1591	315	418	54	56	2315.23237577	0.0
167	20	18	1582788	1540116	1800	325	444	475	481	2575.27793658	0.0
168	20	18	1439993	149236	202	1341	68	51	48	-716.196780896	0.0
169	20	18	14546	1086603	13	3	270	7	9	-178.928468257	0.00264410364886
170	20	18	4647	791352	17	0	336	3	6	-222.669128035	0.00317376355462
171	20	18	3033489	57354	96	1406	23	18	16	-974.35381353	3.86867596958e-07
172	20	18	3033489	57354	96	1406	23	21	16	-974.35381353	3.86867596958e-07
173	20	18	3126376	80006	158	1408	24	19	18	-872.90308028	0.0
174	20	18	3126376	500880	709	1078	164	36	36	221.816195957	0.0
175	21	6	447034	448492	316	98	146	104	105	481.747917395	0.0
176	21	6	192210	192983	184	22	52	47	46	281.08956916	0.0
177	21	8	2577223	2562760	3195	501	628	744	748	4177.90602352	0.0
178	21	8	15559	627419	16	0	0	6	8	31.1459459459	0.000264410364886
179	21	8	642715	627419	641	134	152	10	9	986.722393083	0.0
180	21	8	33121	645798	34	3	571	10	11	-374.708522051	0.000528820729773
181	21	8	27280	878049	13	1	904	5	7	-622.751032395	0.000264410364886
182	21	8	73895	77579	68	23	28	26	26	116.712704602	0.0
183	21	11	33121	54484	7	6	11	3	3	-8.09919733555	0.439
184	21	14	15559	20288	10	12	13	3	3	-11.9966543227	0.415
185	21	15	27280	55212	6	8	50	3	3	-43.5600059026	0.854
186	21	18	67890	57354	18	50	65	4	3	-68.616380808	0.0607489944309
187	21	18	66851	80006	26	42	91	3	3	-86.6401588439	0.675160315019

Continued on next page

Table ST-6 – continued from previous page

	Scaffold		Cluster size (bp)			# Unaccounted exons		# Genes found			
**#**	**n402**	**CBS513.88**	**n402**	**CBS513.88**	**# Hits**	**n402**	**CBS513.88**	**n402**	**CBS513.88**	**score**	**p-value**
188	22	4	40194	39784	46	9	19	13	13	79.0950613153	0.000264410364886
189	24	4	13463	13464	17	1	5	7	7	32.3207320898	0.000264410364886

Table ST-7: *Clusters discovered by i-ADHoRe for the basidiomycota*

	Scaffold		Cluster size (bp)			# Unaccounted exons		# Genes found			
**#**	***S. comm.***	***A. bisp.***	***S. comm.***	***A. bisp.***	**# Hits**	***S. comm.***	***A. bisp.***	***S. comm.***	***A. bisp.***	**score**	**p-value**
1	1	1	570117	461443	658	604	421	99	90	223.407	0.0
2	1	1	691402	726715	846	802	921	105	104	-46.893	0.0
3	1	1	115804	620239	210	77	1240	32	33	-640.172	9.65041380141e-11
4	1	1	646260	164889	246	1120	175	22	20	-550.315	5.6829946804e-07
5	1	1	560811	1567687	1062	451	2754	25	28	-1088.401	0.0
6	1	1	510889	1504479	854	461	2705	17	20	-1289.840	0.0
7	1	1	557784	1676526	1243	311	2813	26	29	-732.331	0.0
8	1	1	646260	248994	301	1074	275	23	22	-522.306	4.31686908442e-11
9	1	1	4119660	233324	766	7522	89	32	26	-4533.262	1.0
10	1	1	3975004	1451285	3742	5500	1241	53	50	-1042.519	0.0
11	1	1	3975004	1445296	3714	5511	1239	55	47	-1073.331	0.0
12	1	1	3953332	2101032	4545	5071	1965	53	45	-702.302	0.0
13	1	1	5545334	1358359	4776	7506	749	36	31	-820.306	0.0
14	1	1	646260	1281143	995	610	2149	28	25	-734.872	0.0
15	1	1	4194992	279927	526	7724	329	32	30	-5048.027	1.0
16	1	1	4183744	1309130	2013	6755	1373	41	43	-4054.845	1.0
17	1	1	524142	1265248	880	515	1906	40	46	-578.480	0.0
18	1	1	3131485	1460833	2078	4835	1580	67	75	-2653.702	0.627412159127
19	1	1	1640897	1152231	1235	2416	1547	31	31	-1469.495	4.03121980241e-13
20	1	1	1478576	1017407	1793	1654	909	23	22	488.086	0.0
21	1	1	4072845	111840	221	7703	109	27	26	-5244.665	1.0
22	1	1	4117755	449153	977	7296	460	30	33	-4556.683	1.0
23	1	1	4070002	1010170	2501	6285	923	27	31	-2548.595	8.35929359067e-09
24	1	1	4038683	924254	2129	6417	866	36	32	-2917.655	0.000116418478754
25	1	1	4117755	990407	2417	6371	1003	29	32	-2779.038	2.66200046105e-05
26	1	1	4117755	941527	2200	6498	970	59	55	-3067.597	0.0292757927048
27	1	1	4043113	285781	466	7555	349	31	36	-5159.187	1.0
28	1	1	404035	162964	394	433	22	62	59	330.986	0.0
29	1	1	59100	2736376	60	70	5336	6	6	-3967.363	1.0
30	1	1	719026	842742	989	644	952	139	134	230.697	0.0
31	1	1	484454	283530	478	653	282	67	68	108.425	0.0
32	1	1	219938	205350	217	209	224	38	39	52.839	0.0
33	1	1	534006	371801	671	582	374	73	69	291.364	0.0
34	1	1	738868	798071	1011	685	861	140	136	288.009	0.0
35	1	1	442699	660116	673	479	1029	66	67	-182.191	0.0
36	1	1	409870	849004	153	752	1768	28	30	-1778.963	1.0
37	1	1	115036	90349	127	133	83	22	21	54.003	0.0
38	1	1	218813	141498	237	244	74	36	35	163.624	0.0
39	1	1	326120	92519	193	441	14	32	32	22.103	0.0
40	1	1	316877	1148759	361	339	2241	30	33	-1440.546	0.0835338996234
41	1	1	546745	618194	558	690	1015	57	55	-515.222	0.0
42	1	1	110956	79833	103	152	29	11	11	55.753	0.0
43	1	1	145614	154218	200	171	266	11	10	-76.077	2.2400636901e-11
44	1	1	325181	219365	183	532	402	34	33	-386.422	1.03553643438e-09
45	1	1	172878	209692	125	194	346	17	17	-214.074	4.68116123642e-07
46	1	1	205410	82010	117	240	114	11	11	-69.949	4.66293670343e-15

Continued on next page

Table ST-7 – continued from previous page

	Scaffold		Cluster size (bp)			# Unaccounted exons		# Genes found			
**#**	***S. comm.***	***A. bisp.***	***S. comm.***	***A. bisp.***	**# Hits**	***S. comm.***	***A. bisp.***	***S. comm.***	***A. bisp.***	**score**	**p-value**
47	1	1	197568	291470	41	379	372	9	9	-490.287	0.999935144932
48	1	1	9561	16585	31	4	15	4	4	38.976	8.72448089339e-05
49	1	1	42490	36591	21	68	64	4	4	-60.057	0.0242626906702
50	1	1	73220	57393	15	22	1	4	4	10.422	8.72448089339e-05
51	1	1	82180	42073	42	116	12	6	6	-28.877	9.80745139975e-05
52	1	1	34878	59996	48	30	100	6	6	-19.501	3.6041845064e-09
53	1	1	15422	128432	26	8	165	5	5	-66.459	8.72448089339e-05
54	1	1	14422	12468	29	9	1	3	3	39.124	8.72448089339e-05
55	1	1	3757	3056	8	2	0	3	3	13.490	8.72448089339e-05
56	1	1	10570	9653	21	2	1	3	3	33.395	8.72448089339e-05
57	1	1	9802	5787	9	6	9	3	3	3.494	0.001875
58	1	1	29731	28207	62	14	5	6	6	89.359	8.72448089339e-05
59	1	1	41862	32420	11	38	50	3	3	-38.912	0.00533006069565
60	1	1	41607	49015	48	52	88	4	4	-28.363	2.07067941793e-07
61	1	1	28609	36614	22	52	69	4	4	-52.712	0.0171349460372
62	1	1	57620	72072	3	131	101	3	3	-172.654	1.0
63	1	1	58012	86252	30	96	191	3	3	-185.029	0.996606351223
64	1	2	483782	798458	105	921	1345	26	27	-1525.102	1.0
65	1	2	122146	432093	19	247	696	7	7	-646.793	0.999981479889
66	1	3	100091	853733	22	114	1486	8	9	-1072.672	0.970093758889
67	1	3	537808	792170	151	1068	1214	23	26	-1399.551	0.99215540107
68	1	3	424625	623194	76	809	887	21	21	-1115.702	0.999999982361
69	1	3	343027	504890	70	443	720	12	12	-649.436	5.95404830844e-05
70	1	3	41296	24404	1	0	0	3	3	0.000	1.0
71	1	4	1048449	1691605	1317	1188	2476	156	172	-1011.697	0.0
72	1	4	331057	229463	505	279	107	67	67	536.533	0.0
73	1	4	149207	1186238	272	83	2038	27	28	-1182.127	0.0019820711713
74	1	4	13401	256769	23	2	554	4	7	-393.834	1.0
75	1	4	33253	272222	36	32	565	6	8	-401.674	0.998621730801
76	1	4	377008	241012	346	252	167	50	48	313.322	0.0
77	1	4	75659	126408	60	85	211	10	10	-123.001	5.04311508738e-05
78	1	4	466479	493782	586	402	638	69	73	151.805	0.0
79	1	4	971088	79452	89	1725	112	12	11	-1193.698	1.0
80	1	4	362003	718189	382	365	885	63	66	-283.766	0.0
81	1	4	335962	206714	354	261	230	43	43	199.490	0.0
82	1	4	128655	66907	123	152	45	19	18	43.984	0.0
83	1	4	209814	192628	253	227	195	30	29	84.713	0.0
84	1	4	184268	124088	166	282	170	19	19	-55.016	0.0
85	1	4	560026	798316	579	519	840	93	96	-58.899	0.0
86	1	4	79958	88743	71	83	125	13	13	-33.094	1.01141317543e-13
87	1	4	391439	260338	178	640	387	23	23	-426.625	1.00586206031e-12
88	1	4	145457	257889	62	232	221	13	14	-210.764	4.19746978707e-05
89	1	4	219134	248098	25	458	546	10	8	-730.278	1.0
90	1	4	183912	348478	203	163	272	36	35	73.293	0.0
91	1	4	50703	36430	40	66	29	7	7	11.477	0.0
92	1	4	141776	135800	133	121	149	24	24	16.033	0.0
93	1	4	70031	20488	20	131	20	6	5	-73.293	0.140756026853
94	1	4	10067	7707	13	1	3	4	4	18.766	8.72448089339e-05
95	1	4	29633	16401	34	10	0	6	6	54.757	8.72448089339e-05
96	1	4	41940	71640	16	41	115	5	6	-87.791	0.256500067702
97	1	4	103510	68544	76	69	65	15	15	31.918	0.0
98	1	4	16636	22042	40	0	20	3	3	46.729	8.72448089339e-05
99	1	4	23924	20775	14	17	29	3	3	-9.381	0.000485721130788
100	1	4	32349	15525	8	60	12	3	3	-34.726	0.138
101	1	4	14233	31771	58	1	48	3	3	54.222	8.72448089339e-05

Continued on next page

Table ST-7 – continued from previous page

	Scaffold		Cluster size (bp)			# Unaccounted exons		# Genes found			
**#**	***S. comm.***	***A. bisp.***	***S. comm.***	***A. bisp.***	**# Hits**	***S. comm.***	***A. bisp.***	***S. comm.***	***A. bisp.***	**score**	**p-value**
102	1	4	49858	52432	7	101	109	3	3	-146.658	1.0
103	1	4	42136	24150	21	83	18	3	3	-44.661	0.212944993201
104	1	4	36315	107800	56	48	101	3	4	1.074	0.0
105	1	4	123090	132672	16	31	323	3	3	-248.296	0.999678812093
106	1	5	73785	77274	4	124	106	3	3	-152.435	0.964
107	1	6	667117	1055630	312	1098	1853	37	47	-1858.534	1.0
108	1	6	1580851	550984	318	2721	924	26	25	-2372.979	1.0
109	1	6	677044	945775	246	1147	1363	40	45	-1549.680	0.999999924758
110	1	6	424895	863589	125	869	1457	26	29	-1544.997	0.999999999999
111	1	6	85874	177157	45	155	250	5	6	-233.460	0.606885215407
112	1	7	118541	161086	22	227	263	4	4	-284.701	0.0705307761644
113	1	7	8877	12935	6	8	9	3	3	0.748	0.000348979235736
114	1	8	377756	617201	78	678	897	17	19	-968.394	0.812181216371
115	1	10	1392390	158188	51	2523	287	13	10	-1938.649	1.0
116	1	10	70012	98464	6	47	27	4	4	-35.202	0.022
117	1	11	114314	931212	33	239	978	8	10	-740.185	0.0041273152463
118	1	11	181342	312624	43	360	458	6	6	-509.080	0.91487269573
119	1	12	93559	953580	99	163	984	16	16	-629.777	2.92097927168e-06
120	1	13	148854	338786	15	315	506	7	8	-530.324	0.904369579456
121	1	14	430944	1434574	96	836	1321	24	31	-1454.247	1.0
122	1	14	229560	262363	274	144	207	38	38	200.458	0.0
123	1	14	239505	251092	172	313	276	32	30	-133.135	0.0
124	1	14	340959	353091	264	449	320	46	47	-133.218	0.0
125	1	14	437166	483488	246	629	636	37	37	-528.471	4.40869563079e-13
126	1	14	267269	259162	327	152	180	43	43	339.407	0.0
127	1	14	130112	140846	62	19	4	10	9	98.574	8.72448089339e-05
128	1	14	142998	202996	81	171	245	11	10	-131.863	0.0
129	1	14	228577	362653	31	440	603	11	10	-701.255	0.999997118242
130	1	14	24242	27332	60	15	23	6	6	66.524	0.0
131	1	14	9407	8095	17	4	3	4	4	22.911	8.72448089339e-05
132	1	14	104938	100456	40	141	135	9	8	-118.910	1.60686033743e-05
133	1	14	214735	217608	28	380	265	5	5	-440.400	0.999999975968
134	1	14	50188	68486	32	78	73	4	4	-46.275	2.62915171967e-07
135	1	14	79716	52990	3	25	2	3	3	-16.713	0.834
136	1	14	82498	33041	3	113	57	3	3	-126.047	1.0
137	1	17	83856	1491118	7	173	542	15	17	-462.493	0.555
138	2	1	523136	613833	109	801	1294	18	20	-1407.499	1.0
139	2	2	2177821	580800	940	3428	518	57	57	-1729.707	0.00691971293762
140	2	2	2087983	1477679	2789	2086	1489	19	20	767.275	0.0
141	2	2	326473	197881	395	281	117	52	52	376.295	0.0
142	2	2	1617853	105049	271	2782	51	32	30	-1535.430	0.639040346683
143	2	2	665547	660448	521	734	758	50	49	-247.469	0.0
144	2	2	2358874	120682	264	4339	60	22	20	-2661.863	1.0
145	2	2	174906	424487	195	136	386	17	18	-69.350	0.0
146	2	2	55610	2458374	42	47	4481	7	7	-3249.753	1.0
147	2	2	338598	449226	466	353	637	48	47	8.140	0.0
148	2	2	1236909	259984	341	1829	349	21	21	-1032.141	6.87738528146e-05
149	2	2	89167	638243	144	80	1398	10	9	-883.392	0.340525034921
150	2	2	799401	1093638	1365	526	1199	124	124	697.318	0.0
151	2	2	367474	437738	392	311	509	55	56	102.891	0.0
152	2	2	464469	659240	744	372	637	61	65	473.021	0.0
153	2	2	299273	300861	363	238	338	36	37	137.172	0.0
154	2	2	310795	423733	413	367	670	51	51	-71.339	0.0
155	2	2	227070	201382	295	196	302	27	28	64.602	0.0
156	2	2	70456	199374	82	98	404	11	12	-243.374	0.00039931026254

Continued on next page

Table ST-7 – continued from previous page

	Scaffold		Cluster size (bp)			# Unaccounted exons		# Genes found			
**#**	***S. comm.***	***A. bisp.***	***S. comm.***	***A. bisp.***	**# Hits**	***S. comm.***	***A. bisp.***	***S. comm.***	***A. bisp.***	**score**	**p-value**
157	2	2	228393	287450	355	145	348	42	45	185.997	0.0
158	2	2	329544	165623	277	428	146	27	26	12.749	0.0
159	2	2	226439	206556	228	212	249	34	35	35.799	0.0
160	2	2	185276	262237	143	214	469	16	16	-239.685	0.0
161	2	2	92044	68441	101	92	99	14	13	55.864	0.0
162	2	2	133765	88665	182	113	85	20	20	112.313	0.0
163	2	2	165052	87861	170	139	42	24	24	153.162	0.0
164	2	2	123554	134380	111	154	192	15	15	-79.039	4.34097202628e-14
165	2	2	151194	157244	123	234	308	16	16	-187.082	1.16018306073e-13
166	2	2	119659	131407	78	164	196	14	14	-135.453	3.42330400249e-07
167	2	2	98722	90185	110	146	140	11	11	-53.093	7.88520360118e-11
168	2	2	62993	133168	75	48	221	16	17	-59.373	0.0
169	2	2	86080	75600	74	68	102	12	12	23.711	0.0
170	2	2	55374	83363	61	64	149	11	11	-57.766	5.33364574729e-09
171	2	2	67057	103493	66	52	120	9	10	-9.076	0.0
172	2	2	178810	115335	51	314	241	6	6	-278.059	4.13716831775e-05
173	2	2	173557	144609	100	157	93	12	11	1.644	0.0
174	2	2	144050	146571	88	156	121	12	12	-9.815	0.0
175	2	2	51851	68186	61	68	83	6	6	-9.279	2.95874436063e-13
176	2	2	18538	46444	25	0	21	4	4	19.518	8.72448089339e-05
177	2	2	122449	111937	41	195	175	4	4	-186.720	0.00517453671888
178	2	2	89366	54366	74	91	89	10	10	-6.120	1.11022302463e-16
179	2	2	85207	120366	21	136	193	6	6	-194.925	0.394059321361
180	2	2	235675	230305	54	330	461	7	7	-439.124	0.00232912231696
181	2	2	4941	4777	14	0	0	3	3	24.265	8.72448089339e-05
182	2	2	19362	20057	31	11	9	4	4	28.315	8.72448089339e-05
183	2	2	7241	7304	17	2	0	3	3	31.574	8.72448089339e-05
184	2	2	11106	13751	30	2	10	3	3	40.667	8.72448089339e-05
185	2	2	31549	55757	61	61	39	4	4	37.454	0.0
186	2	2	20874	13665	25	6	7	3	3	32.134	8.72448089339e-05
187	2	2	34294	36650	57	28	19	5	5	72.717	0.0
188	2	2	28261	7731	16	37	8	3	3	-10.440	0.035
189	2	2	47821	27683	12	63	37	3	3	-41.989	0.00140914265847
190	2	2	15992	51819	2	29	77	3	3	-64.763	0.255
191	2	2	26548	40600	20	37	77	3	3	-58.262	0.289816708805
192	2	2	84806	91931	26	97	59	5	5	-74.596	0.126922156862
193	2	2	52675	54299	69	34	75	8	8	26.608	1.11022302463e-16
194	2	2	44821	17025	31	88	10	3	3	-30.661	0.033
195	2	2	90822	84112	137	48	114	12	12	94.532	0.0
196	2	2	60405	81960	20	96	222	3	3	-194.345	0.58096672684
197	2	2	30648	59886	11	57	135	3	3	-120.528	0.729630956772
198	2	2	41734	99198	73	32	130	3	3	-35.239	0.000262012292656
199	2	3	585032	1187830	410	757	1970	39	36	-1543.824	0.935826247535
200	2	4	235293	244812	34	355	503	13	13	-575.427	0.999993514071
201	2	5	5332	47828	10	3	25	3	3	-4.460	0.003
202	2	7	1302616	168496	80	2448	247	12	10	-1780.346	1.0
203	2	7	498468	552000	98	743	897	16	18	-973.489	0.319294937341
204	2	7	311940	268835	28	488	396	8	8	-524.384	0.109895065624
205	2	7	2546	2681	5	2	1	3	3	3.191	0.107
206	2	9	412622	553721	101	754	712	10	11	-913.120	0.999525410381
207	2	11	520880	654061	86	796	788	23	22	-932.857	0.17429786093
208	2	13	454079	426127	77	708	651	10	9	-775.021	0.00949447652496
209	2	13	173508	392109	12	348	615	7	6	-609.372	0.452536610304
210	2	14	462864	529675	100	643	831	18	17	-864.900	0.23574705508
211	3	1	4057460	654559	529	4472	1174	34	32	-3184.338	0.970192695037

Continued on next page

Table ST-7 – continued from previous page

	Scaffold		Cluster size (bp)			# Unaccounted exons		# Genes found			
**#**	***S. comm.***	***A. bisp.***	***S. comm.***	***A. bisp.***	**# Hits**	***S. comm.***	***A. bisp.***	***S. comm.***	***A. bisp.***	**score**	**p-value**
212	3	1	677452	1130575	182	983	2197	45	47	-2101.891	1.0
213	3	4	2119010	677585	368	3143	963	17	17	-2384.237	0.999769857586
214	3	4	79246	124430	15	144	277	5	5	-305.745	0.999999993362
215	3	5	490579	804752	102	649	1523	38	41	-1413.842	0.999981364566
216	3	5	264204	297954	13	425	505	8	8	-619.455	0.999412122772
217	3	5	442774	437095	61	609	679	12	11	-773.656	0.358513520127
218	3	6	776553	720290	954	612	746	86	91	463.380	0.0
219	3	6	671982	351439	664	572	146	51	42	570.018	0.0
220	3	6	619940	239368	411	701	138	46	41	57.894	0.0
221	3	6	450334	966929	459	402	1376	27	27	-623.790	0.0
222	3	6	573494	1014955	585	544	1408	49	50	-574.370	0.0
223	3	6	2442713	374557	871	3269	239	47	45	-992.665	0.0
224	3	6	464713	633077	689	347	557	72	79	462.256	0.0
225	3	6	669652	30013	22	936	2	6	5	-513.725	0.002875
226	3	6	530847	874675	522	491	1136	62	69	-321.610	0.0
227	3	6	545824	989991	808	361	1044	94	102	239.616	0.0
228	3	6	479961	582332	478	441	740	45	50	-138.508	0.0
229	3	6	826945	901487	777	817	1132	77	80	-331.285	0.0
230	3	6	349479	380790	414	233	364	43	42	253.990	0.0
231	3	6	111701	83306	57	135	112	10	9	-56.795	0.0
232	3	6	135871	202930	84	199	182	12	11	-111.671	1.11022302463e-16
233	3	6	176972	228975	237	160	319	20	22	29.877	0.0
234	3	6	177821	103434	97	275	135	7	7	-154.786	3.69329791209e-05
235	3	6	179901	390455	125	177	409	7	9	-212.170	3.44169137634e-15
236	3	6	155323	180853	78	175	229	10	9	-156.776	1.16790686011e-08
237	3	6	86499	170270	33	147	313	6	6	-278.436	0.61495312546
238	3	6	285124	253804	122	323	361	14	14	-258.267	0.0
239	3	6	53781	82431	64	55	96	5	5	-12.743	4.98595609244e-11
240	3	6	113155	144311	72	167	160	10	9	-93.952	1.66533453694e-15
241	3	6	71666	178286	24	137	179	4	4	-167.181	0.00394023778264
242	3	6	3679	3582	13	0	1	3	3	19.058	8.72448089339e-05
243	3	6	3802	5822	11	0	11	3	3	7.993	0.000959692898273
244	3	6	22746	41752	8	13	11	3	3	0.407	8.72448089339e-05
245	3	6	62727	27110	27	28	16	3	3	-0.179	0.00652287413099
246	3	6	60968	76971	6	58	24	4	4	-56.362	0.996
247	3	6	140380	66394	1	0	0	3	3	0.058	0.674
248	3	9	85923	58972	9	5	0	3	3	6.411	0.034
249	3	10	373283	589112	30	422	599	12	11	-536.724	7.77156117238e-16
250	3	11	557829	357744	54	796	531	19	21	-776.790	0.0847825194634
251	3	13	468548	498613	70	698	709	18	16	-822.410	0.051707604336
252	3	14	630401	503416	91	977	794	26	27	-1126.369	0.999987557447
253	3	14	462057	415896	31	558	690	14	13	-757.949	0.108079402908
254	3	15	357973	437034	41	468	582	11	9	-581.997	6.43602932282e-07
255	3	18	9550	74862	19	20	96	3	3	-54.424	0.0213940172999
256	4	1	293783	183982	23	417	409	8	9	-578.471	0.999999999958
257	4	2	559641	680494	180	759	1568	26	22	-1563.993	1.0
258	4	2	194963	350549	70	263	502	7	6	-459.286	0.837866401282
259	4	3	1572886	2089589	1075	1811	2882	11	12	-2203.348	4.9309319756e-05
260	4	3	352296	363396	164	443	391	8	8	-430.341	0.716081435377
261	4	3	191278	415669	32	303	570	7	8	-552.161	0.833899922781
262	4	3	95790	114708	8	68	93	5	5	-96.498	0.414
263	4	3	49737	23848	5	30	8	3	3	-6.887	8.72448089339e-05
264	4	5	445085	334579	53	568	430	17	15	-592.686	0.484994537524
265	4	5	175717	206547	14	215	329	7	6	-322.560	0.0288147647158
266	4	7	619652	840831	989	430	979	84	89	277.916	0.0

Continued on next page

Table ST-7 – continued from previous page

#	Scaffold S. comm.	Scaffold A. bisp.	Cluster size (bp) S. comm.	Cluster size (bp) A. bisp.	# Hits	# Unaccounted exons S. comm.	# Unaccounted exons A. bisp.	# Genes found S. comm.	# Genes found A. bisp.	score	p-value
267	4	7	690993	835927	811	639	1237	69	75	-330.869	0.0
268	4	7	752896	996800	896	718	1201	85	90	-140.969	0.0
269	4	7	924653	756243	1224	788	787	103	107	433.715	0.0
270	4	7	1243410	184210	279	1615	137	30	25	-681.140	0.0
271	4	7	829545	903567	750	769	1194	92	88	-256.497	0.0
272	4	7	541447	526661	200	641	572	32	26	-462.612	0.0
273	4	7	205243	239809	124	217	303	17	16	-122.141	0.0
274	4	7	235068	232401	102	335	322	17	17	-248.761	0.0
275	4	7	206107	137331	167	249	94	20	19	53.487	0.0
276	4	7	127542	180092	130	90	199	15	14	11.786	0.0
277	4	7	65397	140222	36	62	152	5	5	-123.539	0.880954798669
278	4	7	138645	252701	46	155	328	9	9	-230.343	3.69568098346e-09
279	4	7	87936	91767	56	119	124	7	7	-79.882	9.19068725569e-08
280	4	7	19556	12935	4	22	11	3	3	-3.276	8.72448089339e-05
281	4	7	27795	15851	18	35	11	3	3	15.691	0.0
282	4	7	12185	34068	20	12	49	3	3	-13.583	0.000312816668873
283	4	7	60320	3949	9	75	1	3	3	-32.978	0.036
284	4	7	45169	8867	17	59	7	3	3	-7.275	8.72448089339e-05
285	4	8	4121	3991	5	0	0	3	3	3.497	0.351
286	4	8	42775	28736	3	64	40	3	3	-58.076	0.108
287	4	8	38025	88716	23	36	81	3	3	-13.202	0.0
288	4	9	559002	686956	51	554	937	12	13	-802.131	0.0
289	4	9	46951	72407	15	42	103	3	3	-56.388	1.00441925888e-08
290	4	10	605395	355150	82	749	515	24	24	-601.566	0.0
291	4	10	1588586	236631	205	2218	293	20	21	-1261.370	1.29993238396e-10
292	4	10	427189	225474	67	514	406	20	20	-495.703	0.000499781485129
293	4	10	207615	236631	38	348	352	8	10	-403.731	0.0712255928761
294	4	10	238168	225474	56	290	283	4	4	-297.752	0.00165748975384
295	4	17	69467	40931	2	115	41	3	3	-96.098	0.618
296	4	19	70362	13318	15	71	2	4	4	-2.542	8.72448089339e-05
297	4	19	9254	12362	19	3	21	3	3	15.269	8.72448089339e-05
298	5	1	205764	380871	53	340	762	7	7	-742.980	0.999999780079
299	5	2	720880	390169	98	1284	782	21	20	-1305.469	0.999993298802
300	5	2	370366	542155	97	557	745	16	18	-722.375	0.000657585565974
301	5	3	526035	1875416	872	427	2436	29	38	-796.913	0.0
302	5	3	546156	672251	302	606	708	37	38	-401.478	0.0
303	5	3	463584	954040	303	498	1437	25	27	-905.391	0.0
304	5	3	420416	943557	248	456	1487	32	29	-993.290	3.72940789362e-10
305	5	3	1381513	757493	701	1497	828	18	15	-449.084	0.0
306	5	3	257409	2233667	229	253	2500	16	15	-1462.932	0.0
307	5	3	938457	266951	73	1354	388	11	11	-954.837	1.80056842569e-05
308	5	3	871546	1432857	757	845	1965	11	12	-879.060	0.0
309	5	3	200321	540204	97	227	581	9	10	-388.459	3.86110365902e-10
310	5	3	186468	336337	78	135	541	8	8	-364.450	0.00488807925513
311	5	3	1103468	174220	43	1507	257	7	7	-993.727	0.000679928168429
312	5	3	814475	1379445	1005	860	1628	96	97	-362.589	0.0
313	5	3	287941	196906	114	363	354	14	15	-307.120	2.17125206703e-10
314	5	3	240963	542821	206	365	866	23	24	-507.969	0.0
315	5	3	1747996	303846	301	2560	391	15	15	-1366.684	0.0
316	5	3	315262	405387	254	397	557	31	28	-232.410	0.0
317	5	3	289471	537509	123	455	780	13	12	-633.269	9.51827561213e-09
318	5	3	102125	82240	26	148	127	7	7	-165.885	0.904727935601
319	5	3	6083	6807	16	1	0	3	3	25.164	8.72448089339e-05
320	5	3	9496	12987	35	0	4	5	5	47.572	8.72448089339e-05
321	5	3	6852	11436	21	1	7	3	3	30.265	8.72448089339e-05

Continued on next page

Table ST-7 – continued from previous page

	Scaffold		Cluster size (bp)			# Unaccounted exons		# Genes found			
**#**	***S. comm.***	***A. bisp.***	***S. comm.***	***A. bisp.***	**# Hits**	***S. comm.***	***A. bisp.***	***S. comm.***	***A. bisp.***	**score**	**p-value**
322	5	3	22348	26653	20	27	36	3	3	-20.117	0.0331052240159
323	5	3	35778	53496	18	63	118	3	3	-93.548	0.0192006605445
324	5	3	21525	35904	9	36	48	3	3	-41.926	0.057
325	5	3	38569	65893	18	54	60	3	3	-54.301	0.0788536219878
326	5	3	41967	60167	15	54	71	3	3	-62.149	0.0447855066358
327	5	3	59778	32279	15	86	7	3	3	-48.606	0.656
328	5	4	250328	390037	30	254	522	7	8	-429.081	4.66215097639e-07
329	5	4	153698	158601	23	274	355	5	5	-423.179	0.999942597189
330	5	4	83173	16853	10	118	10	3	3	-53.953	0.000174489617868
331	5	6	404267	705027	164	669	1293	22	30	-1232.255	0.999974961381
332	5	9	156496	57913	12	331	50	4	4	-228.567	0.737389223558
333	5	10	542053	169259	112	746	220	15	15	-485.517	0.00284714479539
334	5	10	327265	525116	41	544	382	10	9	-512.559	0.000216794213956
335	5	13	256735	231660	30	247	346	8	9	-315.448	1.62264872483e-06
336	5	15	971088	679726	178	1278	284	13	14	-725.305	3.5527136788e-12
337	5	15	73888	158998	41	64	128	4	4	-74.747	0.000174824660121
338	5	18	28915	59446	9	54	85	3	3	-74.341	0.029
339	6	2	132624	149659	12	130	195	5	5	-188.109	0.0388847989019
340	6	2	219648	176041	22	238	219	4	5	-303.350	0.999716124226
341	6	4	40712	33550	2	33	32	3	3	-37.916	0.256
342	6	5	143758	336874	65	222	594	6	7	-514.146	0.988336021752
343	6	5	75988	95269	9	109	131	4	4	-148.623	0.61
344	6	6	245595	511835	45	325	940	11	14	-844.027	0.999925531531
345	6	8	719708	176493	53	1035	268	15	15	-736.446	0.0244377549486
346	6	8	271233	397728	49	401	642	10	10	-625.818	0.18131634286
347	6	8	40712	301324	6	0	231	5	6	-154.361	0.777
348	6	8	379319	765230	78	563	751	13	13	-697.016	7.82547360245e-11
349	6	8	87548	92804	10	113	156	4	4 -165.011	0.488	
350	6	10	4077721	1515892	377	2288	457	44	39	-1165.055	0.0
351	6	10	4702261	1486541	450	3097	421	47	37	-1557.634	0.0
352	6	10	789147	1020699	851	602	1094	91	93	111.692	0.0
353	6	10	605395	338032	198	735	367	15	18	-411.944	0.0
354	6	10	460430	2347681	381	386	1550	8	9	-671.701	0.0
355	6	10	240687	1388881	217	211	1092	24	30	-458.694	0.0
356	6	10	261667	170733	80	280	192	15	15	-179.215	8.83149109399e-12
357	6	10	427901	52644	74	540	37	7	6	-183.028	0.0
358	6	10	436626	678454	446	388	829	47	49	-171.018	0.0
359	6	10	348946	416493	333	337	347	33	34	54.649	0.0
360	6	10	307698	394735	309	310	339	31	33	42.494	0.0
361	6	10	145316	86317	84	141	60	10	10	9.494	0.0
362	6	10	121157	213145	5	87	303	4	4	-222.000	0.000174489617868
363	6	10	31312	37223	29	46	43	3	3	-13.866	3.99549328622e-07
364	6	10	35399	52644	15	33	81	3	3	-48.233	0.000214757523817
365	6	13	167314	314226	76	187	416	23	23	-267.117	5.60884672041e-13
366	6	13	223467	305502	102	240	397	13	14	-210.932	0.0
367	6	13	24663	32748	12	0	6	4	4	14.185	8.72448089339e-05
368	6	13	77234	104724	90	65	124	8	8	5.619	0.0
369	6	13	23966	30553	26	31	31	4	4	-6.686	0.000101649974161
370	6	13	22918	21932	38	12	7	4	4	50.647	8.72448089339e-05
371	6	14	49144	44783	1	0	0	3	3	0.000	1.0
372	6	15	498853	561686	380	529	617	30	32	-226.437	0.0
373	6	15	58497	66313	14	83	66	4	4	-50.369	4.98456831366e-12
374	6	17	45324	52335	12	33	13	3	3	-10.111	0.000668226838402
375	6	17	91087	121959	6	22	135	4	4	-78.525	8.72448089339e-05
376	7	1	42801	25492	12	0	0	3	3	16.770	0.000174489617868

Continued on next page

Table ST-7 – continued from previous page

	Scaffold		Cluster size (bp)			# Unaccounted exons		# Genes found			
#	*S. comm.*	*A. bisp.*	*S. comm.*	*A. bisp.*	# Hits	*S. comm.*	*A. bisp.*	*S. comm.*	*A. bisp.*	score	p-value
377	7	2	501538	229983	29	891	504	18	16	-924.607	0.999999913854
378	7	2	185870	229983	13	311	516	7	7	-569.564	0.999996227174
379	7	4	40222	49077	27	44	59	3	3	-12.627	2.0061730055e-13
380	7	4	53595	93152	3	53	214	3	3	-189.574	0.997
381	7	5	191427	133856	256	168	49	31	31	256.694	0.0
382	7	5	290417	464993	320	345	721	36	34	-234.550	0.0
383	7	5	415519	451540	370	572	666	45	39	-280.259	0.0
384	7	5	1195827	133271	111	1996	188	19	17	-1252.346	0.566181138572
385	7	5	375323	89626	222	584	60	9	9	-55.022	0.0
386	7	5	323960	356221	307	396	526	30	30	-150.424	0.0
387	7	5	278361	399047	478	267	425	38	38	164.473	0.0
388	7	5	181530	247348	208	201	269	19	20	3.132	0.0
389	7	5	39013	254791	39	72	294	6	7	-216.423	0.588614704038
390	7	5	194896	306453	116	288	530	19	19	-429.754	0.0314790360963
391	7	5	99171	254451	56	126	347	12	12	-240.777	0.000383540995772
392	7	5	39161	44189	52	63	61	7	7	3.930	4.4408920985e-16
393	7	5	89098	78135	112	110	93	10	10	29.634	0.0
394	7	5	101226	113577	113	109	146	13	14	13.376	0.0
395	7	5	122669	83794	65	115	137	6	6	-51.036	0.0
396	7	5	132565	160314	65	221	323	9	7	-300.352	0.258817362771
397	7	5	64242	148060	45	92	190	9	9	-106.852	1.74074310522e-10
398	7	5	236592	303304	161	268	334	16	16	-144.046	0.0
399	7	5	131310	137510	138	134	160	9	9	22.052	0.0
400	7	5	41108	174936	54	20	238	9	9	-94.472	5.38504674186e-09
401	7	5	35349	89626	60	34	159	6	6	-29.524	0.0
402	7	5	123600	39640	38	181	45	6	6	-76.296	1.31023664141e-08
403	7	5	118325	121318	80	156	217	9	9	-151.634	3.31987199825e-05
404	7	5	20895	15197	32	18	4	6	6	35.321	8.72448089339e-05
405	7	5	27820	45079	19	30	71	3	3	-32.127	1.71921616465e-06
406	7	5	17542	57666	9	19	113	3	3	-89.002	0.967
407	7	5	116222	37559	14	1	0	3	3	22.533	8.72448089339e-05
408	7	7	163912	312382	6	254	324	5	5	-325.767	8.72448089339e-05
409	7	9	3848	18333	4	0	16	3	3	1.300	8.72448089339e-05
410	7	10	115887	152955	18	180	122	5	5	-188.404	0.938989816507
411	7	12	404663	417265	47	582	477	15	12	-676.143	0.999090017055
412	7	12	52191	50894	6	80	84	3	3	-103.545	0.762
413	7	16	289704	531054	141	473	589	19	22	-531.453	3.81395460668e-05
414	7	16	278650	542514	147	445	623	20	22	-526.440	4.60676789416e-06
415	7	16	216316	400202	61	287	447	12	12	-379.805	1.35281402691e-06
416	7	16	134132	160766	29	238	271	3	3	-283.518	0.00823303057071
417	7	16	104427	79905	7	212	142	3	3	-237.529	0.998
418	7	19	3848	4556	5	0	0	3	3	5.367	0.065
419	8	1	567454	460401	69	887	1019	19	21	-1274.670	0.999999999946
420	8	2	866486	176462	67	1415	224	10	8	-954.446	0.524942617139
421	8	2	866486	1520196	397	1242	2895	17	17	-2620.447	1.0
422	8	2	80277	153718	6	165	252	3	3	-270.285	0.838
423	8	3	88542	260705	12	179	261	5	5	-245.332	0.000176703578806
424	8	3	16966	46733	5	3	9	3	3	-6.529	0.826
425	8	4	110518	113183	10	163	204	5	5	-257.646	1.0
426	8	5	274397	248745	225	308	328	37	35	-101.925	0.0
427	8	5	114494	208032	149	92	348	10	10	-81.826	0.0
428	8	5	99316	262906	136	74	288	11	12	-47.373	0.0
429	8	5	460641	351834	380	573	518	45	47	-183.835	0.0
430	8	5	185497	51354	126	186	27	9	9	78.187	0.0
431	8	5	745741	650173	279	1219	935	47	46	-1101.959	8.73653477345e-05

Continued on next page

Table ST-7 – continued from previous page

	Scaffold		Cluster size (bp)			# Unaccounted exons		# Genes found			
**#**	***S. comm.***	***A. bisp.***	***S. comm.***	***A. bisp.***	**# Hits**	***S. comm.***	***A. bisp.***	***S. comm.***	***A. bisp.***	**score**	**p-value**
432	8	5	316166	193495	161	444	224	20	17	-189.841	0.0
433	8	5	211135	211348	184	208	272	18	19	-39.374	0.0
434	8	5	179659	145714	108	289	184	13	14	-165.553	1.17685838852e-09
435	8	5	288461	129667	303	287	56	27	26	244.659	0.0
436	8	5	52392	35100	47	71	45	8	8	-4.548	4.54162263352e-11
437	8	5	160044	69483	28	186	80	7	8	-128.982	0.00455731553416
438	8	5	222604	219132	76	280	471	12	14	-414.461	0.0441663845666
439	8	5	25746	24489	17	42	33	4	4	-35.372	0.397156212661
440	8	5	88936	59977	29	152	120	4	4	-145.379	0.145589333891
441	8	5	5366	5111	13	2	0	3	3	23.205	8.72448089339e-05
442	8	5	21298	23837	29	17	32	3	3	19.280	1.59872115546e-14
443	8	5	18625	41570	18	1	4	3	3	27.105	8.72448089339e-05
444	8	5	12428	22576	20	0	0	3	3	32.354	8.72448089339e-05
445	8	5	49083	71019	31	70	57	5	5	-32.360	1.39073479066e-07
446	8	5	99316	129142	29	149	100	7	7	-141.316	0.710232707816
447	8	7	293220	492480	47	504	963	11	13	-933.051	0.904222173161
448	8	12	139094	184874	20	226	236	4	6	-269.113	0.0958832930119
449	8	13	309026	698431	106	479	1006	16	18	-815.784	5.20766676337e-07
450	8	14	282737	232520	17	405	402	11	9	-542.742	0.999973519784
451	9	2	909351	995563	269	1152	1837	42	44	-1898.183	0.999999999996
452	9	2	364632	248082	36	520	361	13	13	-555.140	0.988051009383
453	9	2	106332	197473	20	155	300	6	6	-267.535	0.0611151738484
454	9	3	72115	44692	11	106	78	3	3	-112.640	0.778441187696
455	9	5	94085	207420	8	145	465	6	5	-445.814	1.0
456	9	8	477858	1137708	295	494	956	33	38	-423.413	0.0
457	9	8	375652	687766	203	422	811	25	30	-474.213	0.0
458	9	8	101481	293781	178	110	306	11	13	-31.579	0.0
459	9	8	10397	20724	10	24	39	3	3	-19.472	8.72448089339e-05
460	9	8	131840	77964	33	110	81	5	5	-54.385	4.83058038014e-13
461	9	9	1375122	184044	365	1706	117	20	20	-605.030	0.0
462	9	9	74020	382625	83	88	425	11	14	-194.793	0.0
463	9	9	85254	250568	43	101	279	9	9	-175.879	4.65452614229e-08
464	9	9	105630	104786	79	125	86	10	10	-11.763	0.0
465	9	9	101259	133600	33	136	240	6	7	-190.716	7.64192046234e-05
466	9	9	25204	31915	31	34	23	5	5	22.234	0.0
467	9	9	85254	116543	28	110	136	6	6	-115.728	0.000237339496258
468	9	9	94507	58154	50	131	54	10	10	-28.709	5.55111512313e-16
469	9	9	77219	153405	86	73	168	8	9	-12.241	0.0
470	9	9	175770	54604	56	234	76	6	6	-105.849	4.59959847987e-10
471	9	12	1221270	327091	579	1593	390	8	9	-473.381	0.0
472	9	12	50867	194859	3	53	256	4	5	-199.873	0.472
473	9	13	383956	623778	157	516	856	19	20	-657.104	1.11022302463e-15
474	9	13	116477	476188	37	200	603	9	13	-453.327	1.60689774276e-05
475	9	13	130410	314249	24	210	279	18	17	-269.238	0.000744092601468
476	9	13	52303	161081	4	103	251	4	4	-232.088	0.793
477	9	17	149830	152191	6	198	257	3	4	-226.062	8.72448089339e-05
478	9	19	72254	115062	1	0	0	3	3	0.000	1.0
479	10	3	103140	87751	25	73	88	4	4	-32.541	0.0
480	10	5	164897	31103	16	248	60	5	5	-162.997	0.0145550927019
481	10	9	66476	114897	14	88	123	5	5	-94.822	3.97178947509e-08
482	10	11	951233	1268914	933	661	1131	104	100	152.097	0.0
483	10	11	380818	978734	288	323	1109	39	42	-429.297	0.0
484	10	11	114396	263650	71	107	297	9	10	-143.945	2.22044604925e-16
485	10	11	517572	580539	171	464	777	28	26	-472.214	0.0
486	10	11	1095305	395230	337	1130	389	40	31	-450.100	0.0

Continued on next page

Table ST-7 – continued from previous page

	Scaffold		Cluster size (bp)			# Unaccounted exons		# Genes found			
#	*S. comm.*	*A. bisp.*	*S. comm.*	*A. bisp.*	# Hits	*S. comm.*	*A. bisp.*	*S. comm.*	*A. bisp.*	score	p-value
487	10	11	577109	196223	83	627	266	15	14	-439.997	6.94398598844e-07
488	10	11	200307	196223	72	225	273	10	10	-242.163	0.00137601209736
489	10	11	302580	255655	104	382	247	12	12	-229.759	0.0
490	10	11	272453	282182	97	277	326	11	12	-225.005	0.0
491	10	11	35583	10931	5	34	9	3	3	-13.499	0.000697958471471
492	10	14	431878	453402	61	564	623	13	13	-661.239	3.96584789374e-05
493	10	14	119529	95172	2	61	131	4	4	-104.804	0.000523468853603
494	11	4	516109	305839	44	688	641	15	14	-838.631	0.975044012341
495	11	7	46415	738480	7	86	1051	10	12	-740.372	0.27
496	11	8	1238706	1050315	1083	997	822	113	117	415.471	0.0
497	11	8	825007	926767	746	819	796	87	89	40.468	0.0
498	11	8	335248	89420	20	315	138	6	5	-254.610	0.0565395557603
499	11	8	419425	335114	310	420	295	37	37	38.734	0.0
500	11	8	495497	297594	232	449	334	21	19	-80.105	0.0
501	11	8	201838	280977	145	233	399	17	17	-198.867	0.0
502	11	8	180809	195104	37	210	210	9	9	-212.769	0.000217398404539
503	11	8	64682	101061	49	94	138	5	5	-66.423	3.73701070089e-13
504	11	8	79541	64462	19	128	100	5	5	-117.015	0.0104693948648
505	11	12	77096	64184	5	120	140	3	3	-170.921	0.937
506	12	3	630605	727072	443	726	1186	40	40	-691.187	0.0
507	12	3	70022	119083	45	96	166	9	9	-119.506	0.00254028948375
508	12	3	320982	303402	68	466	365	11	13	-386.000	1.22124532709e-15
509	12	3	153361	214016	182	142	261	11	11	36.138	0.0
510	12	3	23750	11438	5	23	11	3	3	-13.743	0.059
511	12	3	106462	128905	31	108	205	5	4	-152.263	2.62229886359e-05
512	12	3	130582	174220	36	162	267	4	4	-246.590	0.247186234016
513	12	9	176136	189359	19	190	206	5	4	-220.732	0.00900767342298
514	12	11	299595	215843	52	382	283	9	9	-338.742	6.6093101474e-06
515	12	14	217563	148862	26	313	213	5	5	-312.890	0.499652156429
516	12	19	81311	37183	8	0	0	3	3	11.848	0.000261734426802
517	13	1	4115307	979737	723	630	1574	35	33	-585.732	0.0
518	13	1	4177275	752408	524	789	1303	29	28	-781.106	0.0
519	13	1	3598405	988232	458	254	1777	42	41	-841.361	0.0
520	13	1	210562	189135	406	147	120	49	48	465.472	0.0
521	13	1	440831	274973	270	620	329	44	41	-256.176	0.0
522	13	1	410003	340798	387	395	433	41	41	20.261	0.0
523	13	1	99033	136062	129	90	148	18	18	49.322	0.0
524	13	1	355728	340488	197	529	599	31	29	-509.189	2.35115158453e-08
525	13	1	126611	61339	132	116	27	18	18	130.513	0.0
526	13	1	118620	120630	73	106	181	12	13	-105.184	2.90352010494e-05
527	13	1	285165	171402	118	279	228	23	21	-175.323	6.16990902813e-11
528	13	2	84361	39470	7	133	74	4	4	-149.683	1.0
529	13	5	597451	272271	33	1087	522	14	12	-1171.243	1.0
530	13	6	42705	80636	3	30	41	3	3	-38.928	0.091
531	13	9	170113	265494	11	254	400	5	5	-446.814	0.999891554015
532	13	10	28792	77369	19	26	20	3	3	-7.059	0.00412935212094
533	13	11	760147	464849	147	1211	651	20	17	-1160.032	0.999999948514
534	13	11	76228	72686	4	97	64	3	3	-95.432	0.254
535	13	16	4016405	853175	28	881	362	15	17	-819.495	0.999999851108
536	14	2	92577	171796	8	82	269	3	3	-209.686	0.019
537	14	3	329061	303846	22	418	507	7	7	-531.375	8.76060315417e-05
538	14	5	121349	135251	20	181	265	4	4	-288.946	0.978593571675
539	14	12	152258	161047	59	183	176	8	8	-137.593	4.00461885874e-10
540	14	12	23157	30633	32	10	17	4	4	30.223	8.72448089339e-05
541	14	17	43724	9850	18	64	1	4	4	-16.598	0.006

Continued on next page

Table ST-7 – continued from previous page

	Scaffold		Cluster size (bp)			# Unaccounted exons		# Genes found			
**#**	***S. comm.***	***A. bisp.***	***S. comm.***	***A. bisp.***	**# Hits**	***S. comm.***	***A. bisp.***	***S. comm.***	***A. bisp.***	**score**	**p-value**
542	15	4	48137	46628	0	0	0	3	3	0.000	0.00533006069565
543	15	7	234109	12935	6	8	9	6	3	-0.190	0.00174504842509
544	15	7	378602	12935	13	446	8	9	3	-217.428	8.72448089339e-05
545	15	7	8853	12935	6	8	9	3	3	-0.190	0.00174504842509
546	15	7	11406	12935	5	9	10	3	3	-0.832	0.000348979235736
547	15	10	118250	64937	5	178	48	3	3	-126.510	0.044
548	16	1	133722	49761	71	120	48	10	9	-11.951	3.34163807736e-11
549	16	1	160792	149770	111	108	195	12	12	-19.820	0.0
550	16	1	93138	66045	57	113	69	4	4	-32.157	2.39945840974e-11
551	16	1	141981	61780	76	179	62	9	9	-58.843	9.52416341349e-08
552	16	1	22412	11469	11	34	4	3	3	-4.252	0.000174489617868
553	16	1	37712	81900	22	29	117	4	4	-61.441	6.64684707841e-05
554	16	17	42503	46606	34	39	45	3	3	-5.776	8.93858879136e-08
555	17	1	560944	448808	233	535	500	23	24	-304.919	0.0
556	17	1	308301	274641	115	286	292	15	16	-184.095	0.0
557	17	1	99900	163001	22	151	220	3	3	-199.713	0.000985232288565
558	17	4	379320	448201	37	459	818	12	11	-771.525	0.030237424988
559	18	1	5648514	1411383	142	524	3039	39	33	-2495.575	1.0
560	18	4	144183	175514	76	167	286	10	12	-177.807	3.96671584468e-12
561	18	4	21323	13175	23	15	5	4	4	18.379	8.72448089339e-05
562	18	4	11017	39684	11	4	52	3	3	-8.682	8.72448089339e-05
563	18	6	463455	450592	34	596	733	12	11	-806.295	0.0998411010122
564	18	7	70675	199600	6	77	153	5	5	-109.941	8.72448089339e-05
565	18	11	215467	241003	12	265	215	8	8	-276.811	0.0174502838535
566	18	18	56992	101678	13	73	137	3	3	-113.880	0.0115471515673
567	19	3	67174	209634	15	44	52	3	3	-24.714	3.14396508827e-10
568	19	12	111038	144474	35	151	144	11	11	-130.934	2.51427775699e-06
569	19	12	59070	61986	22	75	30	5	5	-31.679	3.15128846506e-05
570	19	12	119143	152040	45	184	226	4	4	-207.740	0.00183002280801
571	20	7	52396	15209	35	47	3	4	4	26.517	8.72448089339e-05
572	20	7	2546	2681	5	2	1	3	3	3.191	0.107
573	20	10	60569	123759	4	66	206	4	4	-162.701	0.034

Genome A	Genome B	Proteny			i-ADHoRe			
		# Clusters	Mean score	Score std.	# Clusters	Mean score	Score std.	q-value
C. glabrata	*E. gossypii*	110	0.923127	0.166954	135	0.781223	0.185239	1.246377e-14
C. glabrata	*K. lactis*	130	0.735160	0.271611	182	0.750885	0.219614	2.540659e+00
C. glabrata	*L. kluyveri*	133	0.914146	0.148330	180	0.698997	0.224363	6.520996e-30
C. glabrata	*L. thermotolerans*	158	0.799953	0.340221	175	0.711038	0.229115	3.564941e-21
C. glabrata	*L. waltii*	80	0.907625	0.159899	149	0.617764	0.171961	2.548147e-28
C. glabrata	*S. cerevisiae*	211	0.902394	0.215193	233	0.761431	0.254924	1.469852e-26
C. glabrata	*Z. rouxii*	106	0.848399	0.264894	176	0.739260	0.213115	9.938580e-16
E. gossypii	*K. lactis*	162	0.913077	0.137812	139	0.937993	0.107177	6.098682e+00
E. gossypii	*L. kluyveri*	128	0.890786	0.180468	104	0.936843	0.073490	1.884650e+01
E. gossypii	*L. thermotolerans*	145	0.883277	0.211791	117	0.906331	0.126042	1.400519e+01
E. gossypii	*L. waltii*	136	0.875535	0.188300	26	0.666164	0.092586	1.907556e-11
E. gossypii	*S. cerevisiae*	137	0.930417	0.130435	4	0.750408	0.116641	1.179527e-01
E. gossypii	*Z. rouxii*	141	0.912207	0.169651	135	0.927892	0.098276	1.392342e+00
K. lactis	*L. kluyveri*	150	0.873408	0.196178	118	0.904400	0.166237	5.194397e+00
K. lactis	*L. thermotolerans*	170	0.835376	0.287032	311	0.911257	0.148861	4.837099e+00
K. lactis	*L. waltii*	156	0.894872	0.174856	322	0.659217	0.157747	4.116991e-54
K. lactis	*S. cerevisiae*	140	0.926267	0.137344	558	0.604940	0.304815	8.874461e-52
K. lactis	*Z. rouxii*	165	0.890555	0.206644	363	0.907668	0.161675	2.624822e+01
L. kluyveri	*L. thermotolerans*	74	0.659957	0.365502	88	0.890102	0.169950	2.366942e-03
L. kluyveri	*L. waltii*	80	0.811876	0.236020	123	0.619473	0.144880	2.181274e-23
L. kluyveri	*S. cerevisiae*	130	0.899256	0.183383	322	0.486799	0.312124	9.865438e-51
L. kluyveri	*Z. rouxii*	111	0.831319	0.221694	203	0.887558	0.171537	5.585031e+00
L. thermotolerans	*L. waltii*	40	0.437323	0.352406	50	0.565737	0.214099	1.654537e-02
L. thermotolerans	*S. cerevisiae*	157	0.835134	0.301288	395	0.517876	0.317562	3.904145e-50
L. thermotolerans	*Z. rouxii*	133	0.752922	0.337711	236	0.893811	0.151223	8.469869e-02
L. waltii	*S. cerevisiae*	103	0.903161	0.149766	417	0.408249	0.229220	2.210527e-56
L. waltii	*Z. rouxii*	126	0.805062	0.282641	258	0.658332	0.142452	1.721120e-34
S. cerevisiae	*Z. rouxii*	156	0.873448	0.248383	477	0.554868	0.312281	3.884058e-55

Table ST-8. The number of clusters found, the mean and standard deviation of the relaxed score for the clusters found by Proteny and i-ADHoRe. We also report the corrected p-value (q-value) of the kolmogorov-smirnov test between the two epirical distributions of scores. Experiments in which there is no statistically significant difference between the score distributions of Proteny and i-ADHoRe are highlighted in red.

Genome A	Genome B	Proteny		i-ADHoRe	
		A	B	A	B
C. glabrata	*E. gossypii*	2136	2506	1425	2862
C. glabrata	*K. lactis*	3174	3116	1834	5106
C. glabrata	*L. kluyveri*	3282	3445	5300	2762
C. glabrata	*L. thermotolerans*	3033	3059	5137	2401
C. glabrata	*L. waltii*	1219	1307	2816	2110
C. glabrata	*S. cerevisiae*	3486	3502	4991	5773
C. glabrata	*Z. rouxii*	2236	2362	2109	5228
E. gossypii	*K. lactis*	4023	4196	1503	1462
E. gossypii	*L. kluyveri*	4582	5054	1790	1960
E. gossypii	*L. thermotolerans*	4407	4414	1983	2111
E. gossypii	*L. waltii*	4358	5153	278	551
E. gossypii	*S. cerevisiae*	2953	3028	39	28
E. gossypii	*Z. rouxii*	3969	4226	1939	2010
K. lactis	*L. kluyveri*	4851	4888	2457	2155
K. lactis	*L. thermotolerans*	4523	4367	4770	4720
K. lactis	*L. waltii*	4590	5357	4563	8624
K. lactis	*S. cerevisiae*	3056	3100	4518	6972
K. lactis	*Z. rouxii*	4091	4217	4548	4384
L. kluyveri	*L. thermotolerans*	5289	5153	5172	5504
L. kluyveri	*L. waltii*	5222	5848	9870	5394
L. kluyveri	*S. cerevisiae*	4545	4669	6961	5382
L. kluyveri	*Z. rouxii*	5139	4981	5272	5518
L. thermotolerans	*L. waltii*	5190	6064	10,597	5271
L. thermotolerans	*S. cerevisiae*	4080	4336	6961	4941
L. thermotolerans	*Z. rouxii*	4825	4766	4838	4908
L. waltii	*S. cerevisiae*	2890	2701	8834	6942
L. waltii	*Z. rouxii*	5538	4870	4603	8681
S. cerevisiae	*Z. rouxii*	4171	4053	5018	6971

Table ST-9. The number of genes found by Proteny and i-ADHoRe for each experiment.
